# Biologically anchored knowledge expansion approach uncovers *KLF4* as a novel insulin signaling regulator

**DOI:** 10.1371/journal.pone.0204100

**Published:** 2018-09-21

**Authors:** Annamalai Muthiah, Morgan S. Angulo, Natalie N. Walker, Susanna R. Keller, Jae K. Lee

**Affiliations:** 1 Department of Systems and Information Engineering, University of Virginia, Charlottesville, Virginia, United States of America; 2 Department of Surgery, University of Virginia Medical Center, University of Virginia, Charlottesville, Virginia, United States of America; 3 Department of Medicine, Division of Endocrinology and Metabolism, University of Virginia, Charlottesville, Virginia, United States of America; 4 Department of Biostatistics and Bioinformatics, Moffitt Cancer Center, Tampa, Florida, United States of America; 5 Department of Public Health Sciences, University of Virginia School of Medicine, University of Virginia, Charlottesville, Virginia, United States of America; University of Kentucky, UNITED STATES

## Abstract

One of the biggest challenges in analyzing high throughput omics data in biological studies is extracting information that is relevant to specific biological mechanisms of interest while simultaneously restricting the number of false positive findings. Due to random chances with numerous candidate targets and mechanisms, computational approaches often yield a large number of false positives that cannot easily be discerned from relevant biological findings without costly, and often infeasible, biological experiments. We here introduce and apply an integrative bioinformatics approach, Biologically Anchored Knowledge Expansion (BAKE), which uses sequential statistical analysis and literature mining to identify highly relevant network genes and effectively removes false positive findings. Applying BAKE to genomic expression data collected from mouse (*Mus musculus*) adipocytes during insulin resistance progression, we uncovered the transcription factor Krueppel-like Factor 4 (KLF4) as a regulator of early insulin signaling. We experimentally confirmed that KLF4 controls the expression of two key insulin signaling molecules, the Insulin Receptor Substrate 2 (IRS2) and Tuberous Sclerosis Complex 2 (TSC2).

## Introduction

High-throughput profiling techniques are widely used to decipher biological and human disease mechanisms with genomics, transcriptomics, proteomics, epigenomics, metabolomics, and other omics approaches [1–4]. Challenging computational investigations on these large data sets have drawn major attention from systems engineering and computational science communities [5, 6]. *In silico* network reconstruction techniques have been built upon quantitative measurements of relationships such as mutual information, pair-wise correlations, and conditional probabilities among interacting molecules [7–12]. Mutual information (MI) between two genes is obtained by evaluating how much information of one gene is contained in the other after estimating the conditional entropy across their different states of function [8]. These include ARACNE for minimizing indirect and redundant edge identification using Data Processing Inequality [9], time delayed ARACNE for time dependent expression data [10], and three-way MI for complex regulatory gene interactions [7, 11–14]. Partial correlation coefficients have also been used to discover novel gene networks by minimizing redundant edges in the network [15, 16]. Conditional probability-based network inference algorithms have been implemented with dynamic Bayesian network models [17] and approximation of posterior probability computation [18]. Some other widely used network inference approaches are regression based algorithms for known sets of transcription factors and target genes [3, 19], shrinkage techniques [20], and Network Component Analysis [21]. Community-based consensus approaches such as ‘wisdom of crowds’ methods attempt to combine the strengths of various algorithms [22, 23]. Some widely used gene network inference methods are currently available as user friendly modules in the GP-DREAM software [24], such as the Correlation approach that deduces high confidence transcription factor-target gene pairs [22]. While these computational network reconstruction techniques have proven useful in many studies, their limitations have also been well recognized. In particular, the majority of reconstruction techniques result in a large number of false networks that can only be identified by performing costly and laborious biological experiments. Subjective expert knowledge then determines a small number of candidate networks for further investigation.

Therefore, it is critical to significantly reduce the numerous false positive findings from computational analyses and make biological follow up investigations and applications more practical. It is also desirable to more systematically incorporate biological knowledge into novel network inference approaches and thus better enable the discovery of context-dependent molecular mechanisms of interest [3, 25]. To improve upon these shortcomings, we have developed a novel network inference approach, Biologically Anchored Knowledge Expansion (BAKE), which incorporates known biological information of a specific disease condition of interest for gradual network expansion to novel gene interactions. BAKE consists of five sequential analysis steps: 1) biological context-dependent novel network gene discovery, 2) literature mining of known gene networks, 3) identification and integration of novel genes associated with known network genes, 4) expansion of network interactions around known network genes, and 5) *in silico* reverse-confirmation of novel network genes. While these analysis steps have been separately used in previous studies, BAKE explicitly makes alternating sequential use of computational analysis and established knowledge association; *in silico* search → literature mining → integration and association of *in silico* search and literature mining → *in silico* network expansion around known network genes → *in silico* reverse-confirmation and use of literature and data resources for confirmation. This converts an error-prone global (NP-hard) computational network search problem into a manageable local biologically anchored search with a very low rate of false positives.

We have applied BAKE to genomic expression data collected from mouse adipocytes during insulin resistance progression. To replicate genetic predisposition and dietary factors that both contribute to the development and progression of insulin resistance [26], we used a well-established mouse model with double heterozygous deletions of two key early insulin signaling pathway intermediates, the insulin receptor (IR) and insulin receptor substrate 1 (IRS1) [27, 28] and fed them a high fat Western diet. Using BAKE on genomic expression data collected from adipocytes of these mice at different time points, we uncovered novel pathway genes that could contribute to insulin resistance progression in response to a high fat diet. In particular, we identified and experimentally confirmed the transcription factor Krueppel-like Factor 4 (KLF4) as a regulator of the expression of two key insulin signaling molecules, Insulin Receptor Substrate 2 (IRS2) and Tuberous Sclerosis Complex 2 (TSC2).

## Materials and methods

### Animal model of insulin resistance

**Fig 1:**
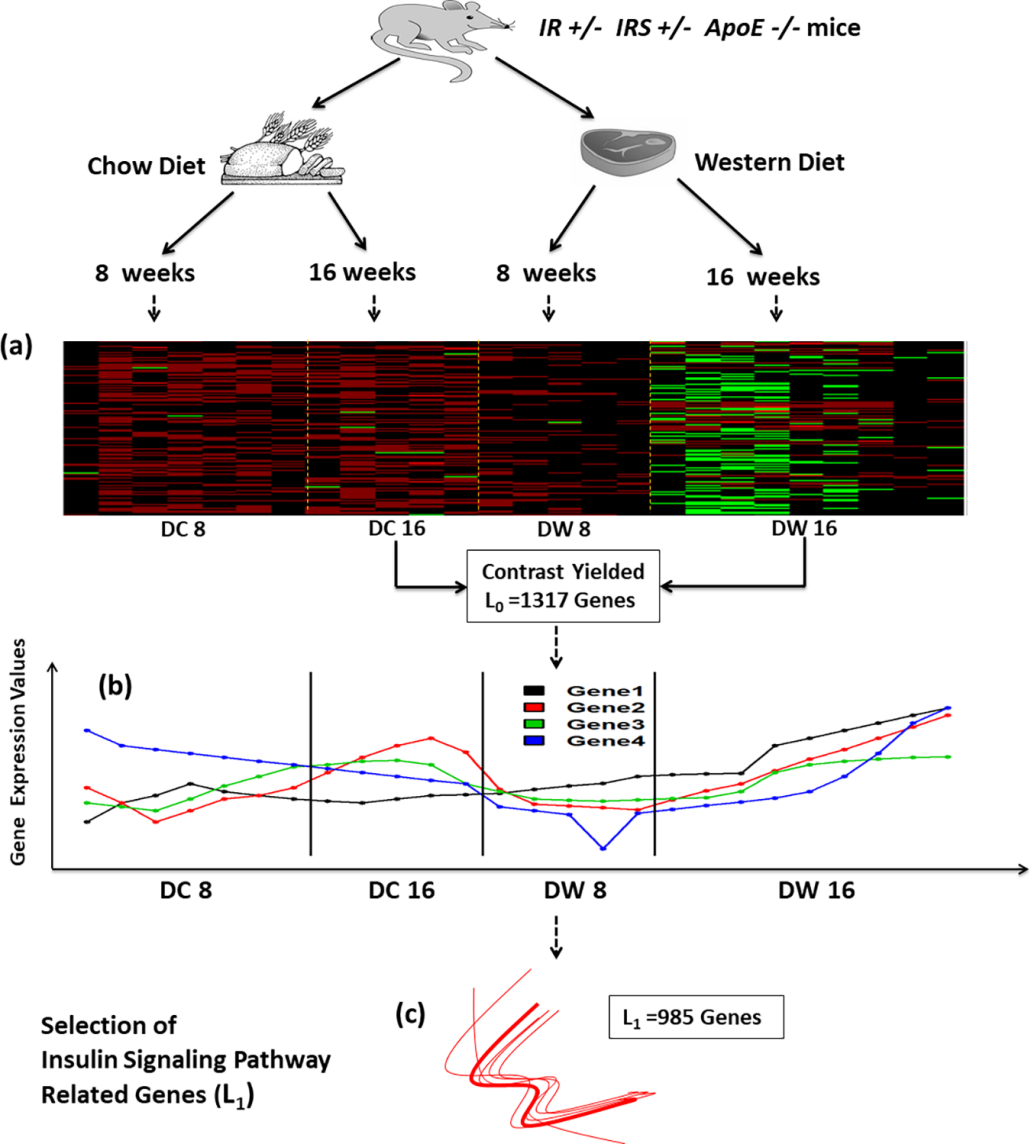
Experimental design and demonstration of BAKE steps 1 and 3. *IR*^*+/-*^*IRS1*^*+/-*^*ApoE*^*-/-*^ mice were fed either a Western (W) or Chow (C) diet. Microarrays were obtained from adipocyte RNA isolated from these mice after 8 and 16 weeks on the diets (DC8, DC16, DW8 and DW16). (a) A subset of gene expression data from the microarrays is shown as heat map where rows represent different genes and columns data from different mice. Differentially expressed genes (L_0_ = 1317 genes represented by 1531 Affimetrix probes) were extracted comparing DW16 (n = 9) and DC16 (n = 5). (b) Complete expression profiles of all genes in L_0_ under all four conditions for each mouse, DC8 (n = 7), DC16 (n = 5), DW8 (n = 5) and DW16 (n = 9), were obtained. Examples are shown for four genes (Genes 1–4). (c) The complete expression profiles of insulin signaling pathway genes (L_path_) were used to extract genes from L_0_ that had similarly shaped expression profiles as pathway genes yielding L_1_.

Female *C57BL/6* mice heterozygous for deletions of the insulin receptor (IR) and the insulin receptor substrate 1 (IRS1) on an *ApoE* null background (*IR*^*+/-*^ *IRS1*^*+/-*^ *ApoE*^*-/-*^; referred to as *D*) [28] were fed a Chow (DC) (7912 Teklad LM-485, Harlan Laboratories) or a Western diet (DW) (88137 Harlan-Teklad composed of (in % by weight) protein 17.3%, carbohydrate 48.5%, fat 21%) for 8 or 16 weeks starting at 8 weeks of age (Fig 1). *ApoE* null mice (*ApoE*^*-/-*^) were also fed either Western diet (EW) or Chow diet (EC) for 8 or 16 weeks starting at 8 weeks of age. Random-fed mice were then euthanized between 8–10 AM. Blood glucose was determined in a drop of tail vein blood before euthanasia. For the determination of circulating insulin levels, blood was obtained by cardiac puncture after euthanasia and serum used in an insulin radioimmune assay (Linco Research, Cat. No. SRI 13K). All animal procedures were approved by the University of Virginia Animal Care and Use Committee. Mice were euthanized by cervical dislocation after induction of deep anesthesia using 100% CO2 inhalation. This method is consistent with the recommendations of the Panel of the American Veterinary Medical Association.

### Adipocyte gene expression profiling during insulin resistance progression

Adipocytes were isolated from parametrial and periovarian adipose tissues of the mice as described in [29], and total RNA was extracted from the adipocytes using TRIzol (Life Technologies Corporation) according to manufacturer’s instructions. cDNA was synthesized from the total RNA (two step protocol) and used to prepare biotinylated cRNA for subsequent genome-wide gene expression profiling using Affymetrix MG430-2.0 GeneChips™. Microarray data were obtained from adipocytes from 45 mice under different diet conditions and disease-progression time points: DW8 (n = 5), DW16 (n = 9), DC8 (n = 7), DC16 (n = 5), EW8 (n = 6), EW16 (n = 8) and EC16 (n = 5). Each microarray chip covered ~ 45,000 mouse gene transcripts. The raw intensity values of hybridization were scanned and stored as .CEL files using Affymetrix Microarray suite software with global scaling option. Gene expression values were then obtained by reading and normalizing the .CEL files using Robust Multi Average (RMA) express software. The microarray data sets (one from each mouse) have been uploaded to the GEO database (accession number GSE76428).

### Discovery of novel genes associated with insulin resistance progression and literature mining for known insulin signaling pathway genes (Steps 1 & 2)

The initial pool of genes for network expansion (L_0_) was obtained by comparing gene expression data between DC16 (n = 5) and DW16 (n = 9). This comparison showed the largest number of differentially expressed genes when contrasting different time points using a combination of algorithms that were developed for small samples of microarray data, Linear Models and Empirical Bayes (LIMMA) [30] and Significance Analysis of Microarrays (SAM) [31]. Two sets of genes (represented by Affimetrix probes) were identified to be differentially expressed between the two conditions, DW16 and DC16, at 1% False Discovery Rate (FDR), by the two methods, LIMMA and SAM. We compared the two lists and selected the genes/Affimetrix probes common between them to obtain L_0_. LIMMA and SAM were implemented in statistical software R using packages “limma” and “samr”. We next identified known genes in the insulin signaling pathway from the KEGG (Kyoto Encyclopedia of Genes and Genomes) database and the literature to obtain L_path_.

### Identification of novel genes associated with known insulin signaling network genes (Step 3)

We used an integration and association analysis for our bioinformatics findings and known insulin signaling network genes as follows. L_1_, representing genes associated with the pathway/network of interest (L_path_), was obtained by correlating expression levels of genes in L_0_ with expression levels of genes in the insulin signaling pathway (L_path_, 42 known genes represented by 80 Affymetrix probes) across the four conditions DC8, DW8, DC16, DW16 using Spearman’s rank correlation. The threshold above which the degree of correlation between genes in L_0_ and L_path_ could be considered significant and a gene in L_0_ classified as pathway/network associated gene was determined based on a resampling-based method as follows. A list of non-network/pathway genes (L_pathrandom_) was generated by excluding genes from the full genome (of 45,037 Affymetrix probes) that were differentially expressed in every possible contrast between microarrays representing the four experimental conditions, and then randomly sampling an identical number of probes (L_pathrandom_, 80 Affimetrix probes) as used for pathway probes (L_path_) from the remaining pool of ~ 35,000 genes/probes (L_random_). The degrees of correlation between genes in L_0_ and L_path_, and between L_0_ and L_pathrandom_ were compared using the top fifth percentile population values for each gene in L_0_ (since the statistical level of significance (α) we decided to use in estimating the correlation cut-off for selecting pathway/network associated genes from L_0_ was 5%) using Response Operator Characteristics (ROC) curves for different threshold values. This ROC comparison was performed 100 times by repeatedly sampling L_pathrandom_ from L_random_ (S1 Fig). The optimal correlation threshold to select pathway specific genes from L_0_ was determined based on Youden’s J index derived from the ROC curves (S1 Fig). Genes from L_0_ that exceeded this correlation threshold in their degree of correlation to L_path_ genes were considered novel pathway/network associated genes (L_1_).

### Expansion of networks around known network genes (Step 4)

We used a systematic network expansion analysis for novel and known network genes as follows. Anchor genes (L_anchor_, 15 genes represented by 20 Affimetrix probes), serving as focal points for network expansion, were selected from pathway genes (L_path_) with the criterion that they were differentially expressed between DW16 and DC16 at a 1% p-value cutoff using LIMMA and SAM algorithms. To expand the network around an anchor gene, we identified the top 20 genes from L_1_ that were most highly correlated to the anchor gene across the four conditions DC8, DW8, DC16, DW16 (L_neighbor_). To decipher the relationship between the novel genes in L_neighbor_ and the 15 anchor genes (20 probes) in the pathway, a 40 x 40 symmetric distance matrix ([X]_real_) consisting of correlation distances between the 40 probes (L_total_ = L_anchor_ + L_neighbor_) was generated. The degrees of network interactions were then inferred by the Super Paramagnetic Clustering (SPC) algorithm [32]. SPC can show gene interaction strengths along a temperature gradient from T = 0 (coldest) to 1 (hottest) (at intervals of 0.01) with the number of nearest neighbors for clustering set at K = 10. As the temperature of simulation was increased, only edges between genes that were strongly correlated in terms of their expression profiles survived. Novel network genes were then assigned to the most tightly clustered candidate neighbors. The number of novel genes introduced into the network as neighbors depended on the number of anchor gene probes. We recommend the total number of objects to be clustered by SPC algorithm, L_total_ (L_anchor_ + L_neighbor_) to be 40. Based on our experience with the SPC algorithm, performing clustering on 40 objects at K = 10 produced stable clusters of 3 to 5 genes, ideally sized to study interactions between anchor and novel genes. Therefore, the number of novel gene probes introduced in L_neighbor_ was adjusted based on the number of anchor gene probes, and we suggest to keep L_anchor_ at 20.

**Fig 2:**
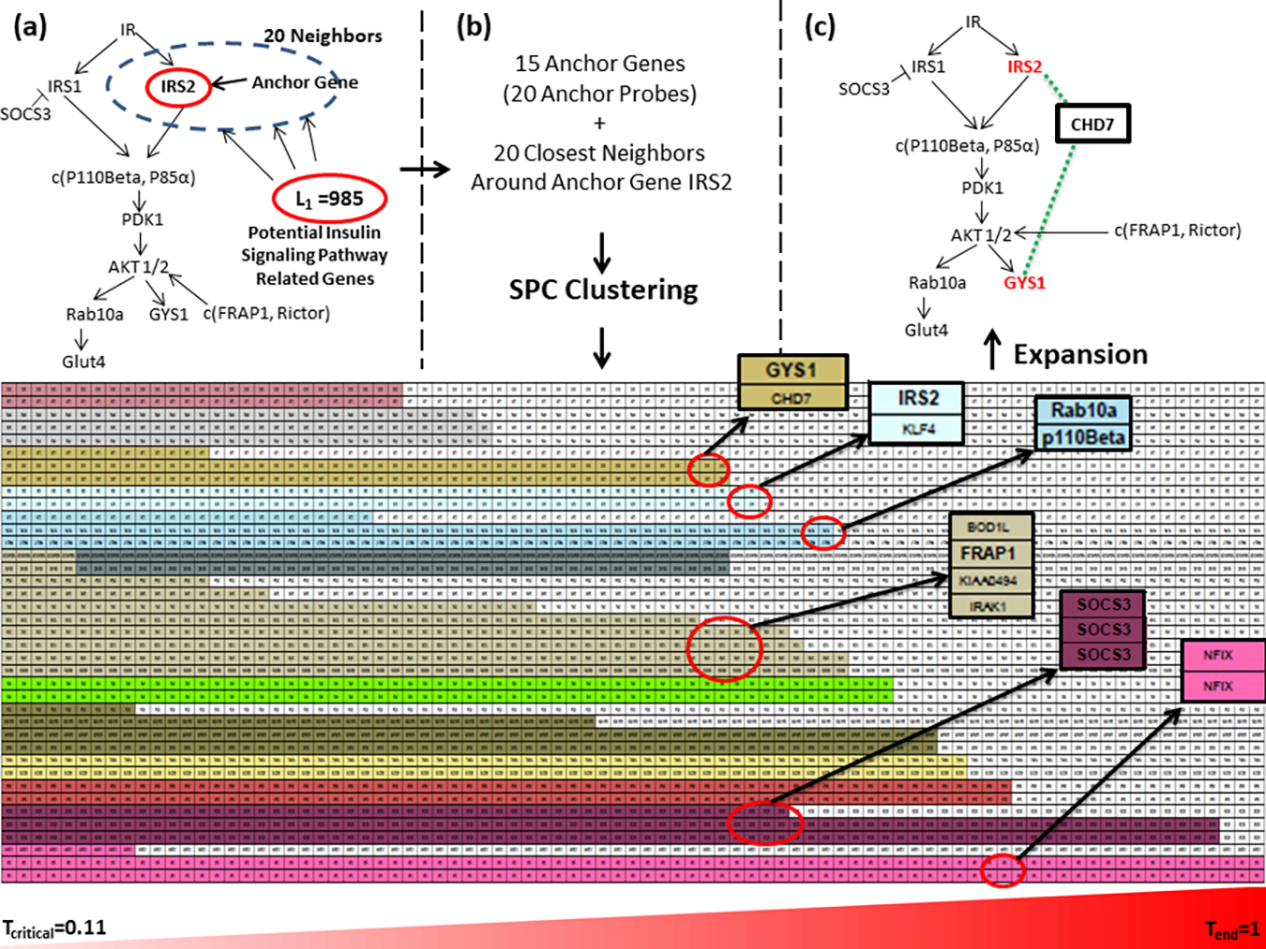
Knowledge/network expansion around insulin signaling pathway genes (BAKE Step 4). Network expansion is demonstrated using the anchor gene IRS2 as an example. (a) The top 20 genes (neighbors) in L_1_ whose expression most highly correlated with IRS2 expression across all four conditions (DC8, DC16, DW8 and DW16) were selected. (b) Super Paramagnetic Clustering (SPC) in the presence of a rising temperature gradient was performed to uncover relationships between the 20 neighbors of IRS2 and the 15 anchor genes in the insulin signaling pathway for a total of 35 genes represented by 40 Affimetrix probes. The relative strengths of associations between the novel network genes and anchor genes is reflected in the extent clusters survived when exposed to increasing temperatures. Temperature profiles of a selection of clusters (marked by different colors) are shown in the bottom panel (the same profile is shown at greater resolution in S2 Fig). The temperature gradient ranging from T_critical_ (0.11) to T_end_ (1.00) with incremental temperature steps of 0.01 is shown as an ascending triangle at the bottom of the temperature profiles. T_critical_ was the lowest temperature at which cluster formation patterns could be distinguished upon analysis of observed and random gene expression data (S4 Fig and Materials and methods). Several stable gene clusters identified by SPC algorithm are circled and magnified in insets. Anchor genes within clusters are in bold. Tight clustering of different probes from the same gene, as shown for SOCS3 and NFIX genes, validated that SPC clustering identified stable gene relationships at higher temperatures. (c) Network was expanded around insulin signaling pathway genes (anchor genes) based on stable clusters identified by SPC.

We conducted SPC clustering on two sets of genes: 1) [X]_observed_, observed correlation distance matrix generated from observed expression profiles of 40 probes (a combination of the 15 anchor genes represented by 20 Affimetrix probes (L_anchor_) and 20 novel probes (L_neighbor_)), and 2) [X]_i,random_ (i = 1 to 100), random distance matrices generated from random gene expression profiles. Random gene expression profiles were generated by permuting the observed gene expression profiles (L_anchor_ and L_neighbor_ genes) and repeating it 100 times. The reason for using permuted random gene expression profiles was to obtain a biologically relevant critical/threshold temperature (T_critical_) at which real gene associations can be distinguished from random cluster formation. While the input for SPC clustering is the distance matrix, output is a temperature profile of clusters in the form of a matrix ([Y]) that provides the cluster membership of each gene at each temperature and chronicles the history of gene clusters from T = 0 to T_end_. At T = 0 all the genes form one cluster. While the temperature is raised the large cluster gradually falls apart into smaller more stable sub-clusters until at T_end_ all clusters fall apart. The average (and standard deviation) of maximum cluster sizes at each temperature was estimated (from the output matrix Y, temperature profile of clusters) and compared between observed and random cases. T_critical_ was determined as the lowest temperature at which the maximum cluster sizes for observed was significantly higher than for random cases (S4 Fig). Starting at T_critical_ the temperature profile of clusters, generated as output of SPC algorithm ([Y]), was converted to a "visual representation" of clusters by assigning a color to each cluster. A gradual shrinkage of clusters with rising clustering temperature can then be observed. This allowed us to identify strong associations between novel genes and anchor genes. Temperature profiles of clusters generated for anchor gene IRS2 are shown in Fig 2B and S2 Fig.

### Confirmation experiment of IRS2 and TSC2 regulation by KLF4

Tamoxifen-inducible KLF4-deficient mice (*ERT-Cre*^*+/-*^*/Klf4*^*loxP/loxP*^) were generated as described in [33]. For conditional deletion of KLF4, female mice heterozygous (*ERT-Cre*^*+/-*^*/Klf4*^*loxP/+*^) and *wild type* (*ERT-Cre*^*+/-*^*/Klf4*^*+/+*^) for the conditional allele were treated with tamoxifen (62.5 mg/kg body weight)(Sigma-Aldrich Co, Cat. No. T5648) in peanut oil (Sigma-Aldrich Co, Cat. No. P2144) by intraperitoneal injection starting at 6 weeks of age. Five days of treatment were followed by 2 days of rest and another 5 days of tamoxifen injections. At 12 weeks of age mice were euthanized. Parametrial and periovarian white adipose tissues were dissected, frozen in liquid nitrogen and stored at –80°C until RNA isolation. Total RNA was prepared from adipose tissues using TRIzol Reagent (Life Technologies Corporation) according to manufacturer’s instructions. cDNA was synthesized from 2 microgram of total RNA using 1 micromolar oligo-dT (Life Technologies Corporation) and M-MLV reverse transcriptase (Life Technologies) in 20 microliter total volume following manufacturer’s instructions. To the completed reaction 80 microliter H_2_O was added to obtain 100 microliter of diluted cDNA. Real time PCR was then performed on 6 microliter of the diluted cDNA (1X) using the qSTAR Expression Detection System (OriGene Technologies Inc) with IRS2 primers (Cat. No. MP206573), TSC2 primers (Cat. No. MP217683), KLF4 primers (Cat. No. MP207225) and MicroTubule-Associated Protein 1B primers (MTAP1B, Cat. No. MP208171), and the SensiMix SYBR Master Mix. MTAP1B was used as a negative control as its expression was not expected to be affected by changes in KLF4 expression. For PCR amplification the following conditions were used: initial denaturation and enzyme activation step at 95°C for 10 min followed by 42 cycles with denaturation at 95°C for 15 sec, annealing/extension/collection of data at 60°C for 60 sec. A melt curve analysis was performed for each reaction to confirm the amplification of single products. A standard curve for each primer set was obtained using different amounts of total cDNA input (1X, 1/2X and 1/4X) for one of the wild type samples and plotting the threshold cycles versus the log of cDNA input. Relative gene expression levels of IRS2, TSC2, KLF4, and MTAP1B in each sample were then derived from the standard curve.

## Results

### Progression of insulin resistance in the animal model

Female *IR*^*+/-*^ *IRS1*^*+/-*^ *ApoE*^*-/-*^ mice were fed a Western (high-fat) diet for 8 (DW8, n = 5) and 16 weeks (DW16, n = 9) starting at 8 weeks of age or a regular chow diet (DC8, n = 7; DC16, n = 5) (Fig 1). Body weights, pooled parametrial and periovarian adipose tissue weights, blood glucose, and plasma insulin levels were determined at the time of euthanasia (S1 Table). Consistent with higher fat intake adipose tissue weights (relative to body weights) were higher, by 21% (p = 0.06) and 40% (p = 0.08), respectively, for DW8 and DW16 mice when compared to DC8 and DC16 mice. Body weights were significantly higher in DW8 mice than DC8 mice (p = 0.015), but they were similar for DC16 and DW16 mice. No significant differences for blood glucose and serum insulin levels between the different groups were found. The small and non-significant differences in body weights are most likely due to the fact that female *C57BL/6* mice demonstrate only minor body weight gains after 16 weeks on a Western diet [34]. The lack of increases in glucose and insulin levels in response to a high fat diet in our mice on an *ApoE*^*-/-*^ background is consistent with an earlier study that showed resistance to diet-induced metabolic changes in *ApoE*-deficient mice [35].

### Discovery of novel genes associated with known insulin signaling pathway genes

We analyzed genome-wide gene expression data to computationally discover genes that were differentially expressed in adipocytes of western and chow diet-fed mice. For this we simultaneously used two widely used statistical test procedures for microarray data analysis; LIMMA and SAM adjusted for multiple comparisons [30, 31]. While the comparison between DW8 and DC8 showed only 51 (represented by 52 Affymetrix probes) differentially expressed genes, a much larger number of genes were differentially expressed between DW16 and DC16 at a false discovery rate (FDR) <0.01 (1,317 genes represented by 1531 Affymetrix probes, denoted as L_0_ genes) (Fig 1A, S2 Table). Analysis of potential molecular and cellular functions of genes in L_0_ using QIAGEN’s Ingenuity Pathway Analysis tool (IPA^®^, QIAGEN Redwood City, CA, http://www.qiagen.com/ingenuity) revealed that 33% played roles in cellular growth and proliferation and 31% in cell death and survival. Network enrichment analysis based on functional annotation of genes revealed genes belonging to gene networks associated with cellular assembly and organization, molecular transport, RNA trafficking, cell cycle, and inflammatory and infectious diseases.

We next identified 42 known genes (represented by 80 Affymetrix probes) in the insulin signaling pathway from the KEGG (Kyoto Encyclopedia of Genes and Genomes) database and the literature [36] (L_path_, Suppl. Table S3)(Step 2: Literature mining of known gene networks). To uncover novel genes linked to impaired insulin signaling, we then extracted genes from L_0_ that were strongly associated with the known insulin signaling pathway genes in L_path_ (Step 3: Identification of novel genes associated with known network genes). Specifically, we correlated adipocyte gene expression patterns of L_0_ and L_path_ for all the 26 animals in the different groups (DC8, DW8, DC16 and DW16) to capture possible functional associations across the different conditions representing type and duration of the diet (Fig 1B). L_0_ genes that were significantly correlated (Spearman’s rank correlation ρ≥0.72) with known insulin signaling pathway genes were considered candidate novel pathway genes (L_1_ = 985 genes represented by 1,111 Affymetrix probes, Fig 1C and S2 Table)). The Spearman’s rank correlation threshold ρ≥0.72 was established as the optimal cutoff after maximizing the Youden’s J index (= sensitivity+specificity-1) from an ROC (Response Operator Characteristic) analysis comparing the correlation coefficients of L_0_ genes with insulin signaling pathway genes and those of non-insulin signaling pathway genes (S1 Fig).

### Expansion of network interactions around known network genes (Step 4)

We selected 15 anchor genes (represented by 20 Affimetrix probes), L_anchor_, among the known insulin signaling pathway genes (L_path_) that showed significant expression changes between DW16 and DC16 with SAM test p-value <0.01 (S3 Table). Novel network genes that interacted with anchor genes across all four conditions were then identified. We first expanded the gene network around IRS2, a well-characterized early mediator in the insulin signaling pathway, by identifying the top 20 genes among L_1_ genes that were most highly correlated with IRS2 as candidate novel network genes (L_IRS2_) (Fig 2A; S4 Table). Potential network relationships and proximity between the anchor genes and the 20 novel candidate genes were then further explored by Super Paramagnetic Clustering (SPC). SPC is a temperature annealing-based clustering technique that allows gradual selection of gene clusters based on degrees of correlations of expression patterns [32] (see Materials and Methods for more details). We applied unsupervised SPC analysis to 35 genes; the 20 novel genes around IRS2, and the 15 anchor genes (L_anchor_) (top panel in Fig 2B). While increasing the temperature parameter, SPC gradually finds clusters with more stable relationships between IRS2, other anchor genes and novel genes in an unsupervised manner. Examples for temperature profiles of clusters are shown in Fig 2B (bottom panel; magnified view in S2 Fig) with stable clusters highlighted. Validating our approach, the most stable clusters were identified between different Affymetrix probes derived from the same gene, SOCS3 and NFIX. Since our goal was to expand our known network of genes, we excluded clusters with only novel genes and focused on the tightest clusters containing one novel gene and one or more anchor genes for further analysis. Examples of such are IRS2-KLF4 and GYS1-CHD7 clusters. CHD7 was initially discovered as one of the 20 neighbor genes around anchor gene IRS2. SPC clustering revealing a tight interaction of CHD7 also with the anchor gene GYS1 thus allowed to infer novel network relationships between IRS2, CHD7 and GYS1 (Fig 2C).

**Fig 3:**
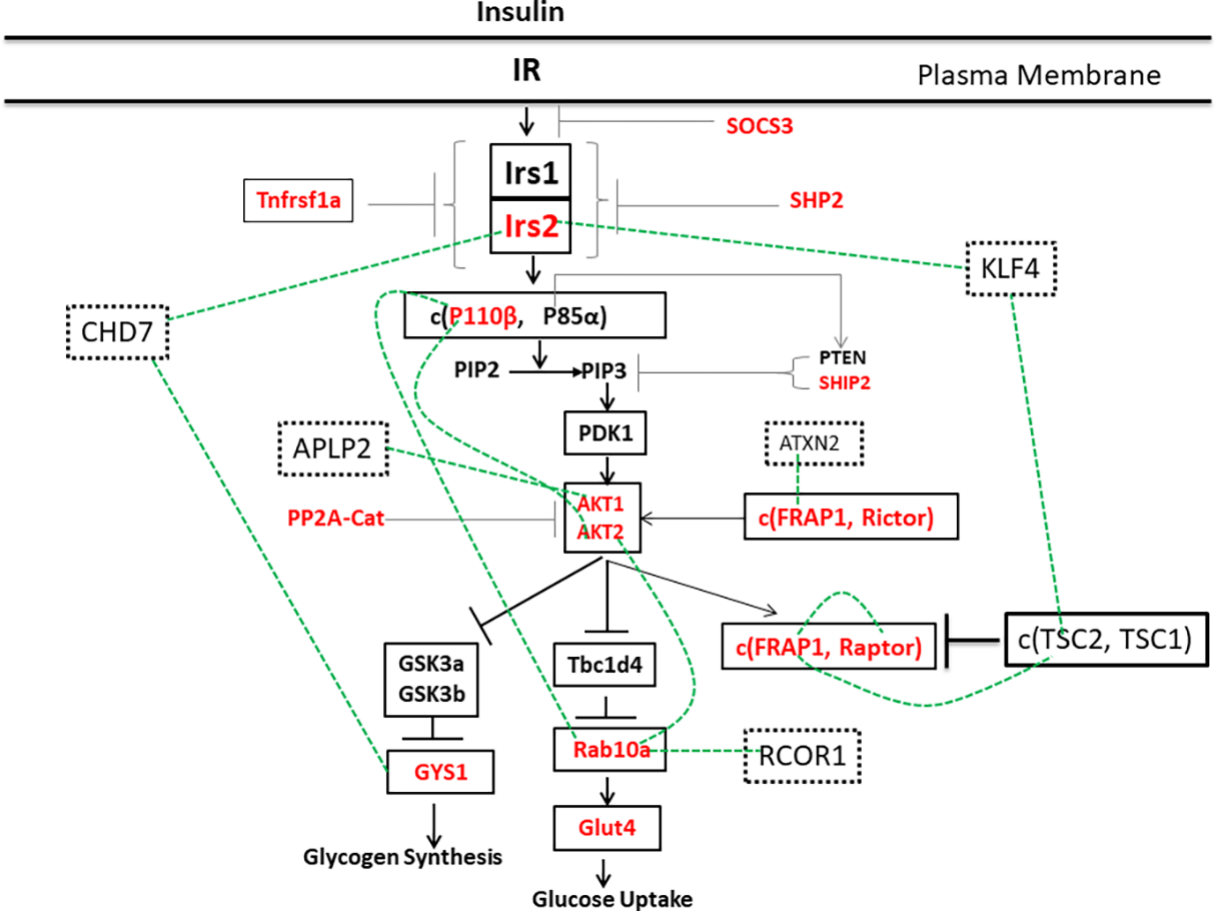
Network expansion around anchor genes in the insulin signaling pathway. A simple representation of key intermediates and modulators of the insulin signaling pathway is shown. Anchor genes in the pathway are marked in red and novel genes introduced into the network by BAKE are given in dashed boxes. Relationships between novel genes and anchor genes and between different anchor genes identified by BAKE are indicated by green broken lines. Gene associations were derived from the following BAKE clusters that were obtained using the 15 anchor genes together with the 20 closest neighbors of different anchor genes (anchor gene used given in parentheses, anchor genes forming clusters given in bold): KLF4-**IRS2**-TSC2, CHD7-**GYS1** (IRS2); ATXN2-**FRAP1**, RCOR1-**Rab10** (P110ß), ALPL2-**AKT1** (AKT2), **P110ß-AKT2-Rab10** (AKT1) and TSC2-**FRAP1-Raptor** (FRAP1).

To further expand the insulin signaling pathway network, we determined the 20 closest neighbors around other anchor genes and performed SPC clustering with those and the 15 anchor genes (20 probes). We again examined tight clusters containing one or more anchor genes but not more than one novel gene. This analysis added novel genes to the network around anchor genes but also identified significant relationships between anchor genes (Fig 3). Among anchor genes, strong cluster formation was observed between FRAP1, Raptor and TSC2. FRAP1 and Raptor form the mTORC1 complex whose activity is controlled by the extent of TSC2 phosphorylation [37]. Coordinated feedback regulation may thus simultaneously affect FRAP1, Raptor and TSC2 gene expression. Other tight associations identified for anchor genes were between the catalytic subunit of phosphatidylinositol-3 kinase (p110β) and Akt2, p110β and Rab10a, and Akt2 and Rab10a (Fig 3). The proteins encoded by these genes are key components of the insulin signaling pathway that regulates cell surface localization of the glucose transporter GLUT4 and hence glucose uptake (Fig 3) [38].

Based on their interactions with different anchor genes five novel genes were introduced into the network: Chromodomain Helicase DNA binding protein 7 (CHD7), Amyloid beta (A4) Precursor-Like Protein 2 (APLP2), Ataxin-2 (ATXN2), and Kruppel-Like Factor 4 (KLF4) and REST CoRepressor 1 (RCOR1) (Fig 3). The inferred interactions are supported by the literature or databases as following. Interaction between the APLP family of molecules and AKT1 has been observed before [39]. Ataxin-2 modifies the abundance of several translation factors while mTOR (FRAP1) associated with Raptor regulates translation through S6 kinase [40]. Rab10 was determined as a potential transcriptional target of RCOR1 using ChiP-seq datasets by the Encyclopedia of DNA Elements (ENCODE) project [41, 42].

### *In silico* reverse confirmation of novel network genes (Step 5)

**Fig 4:**
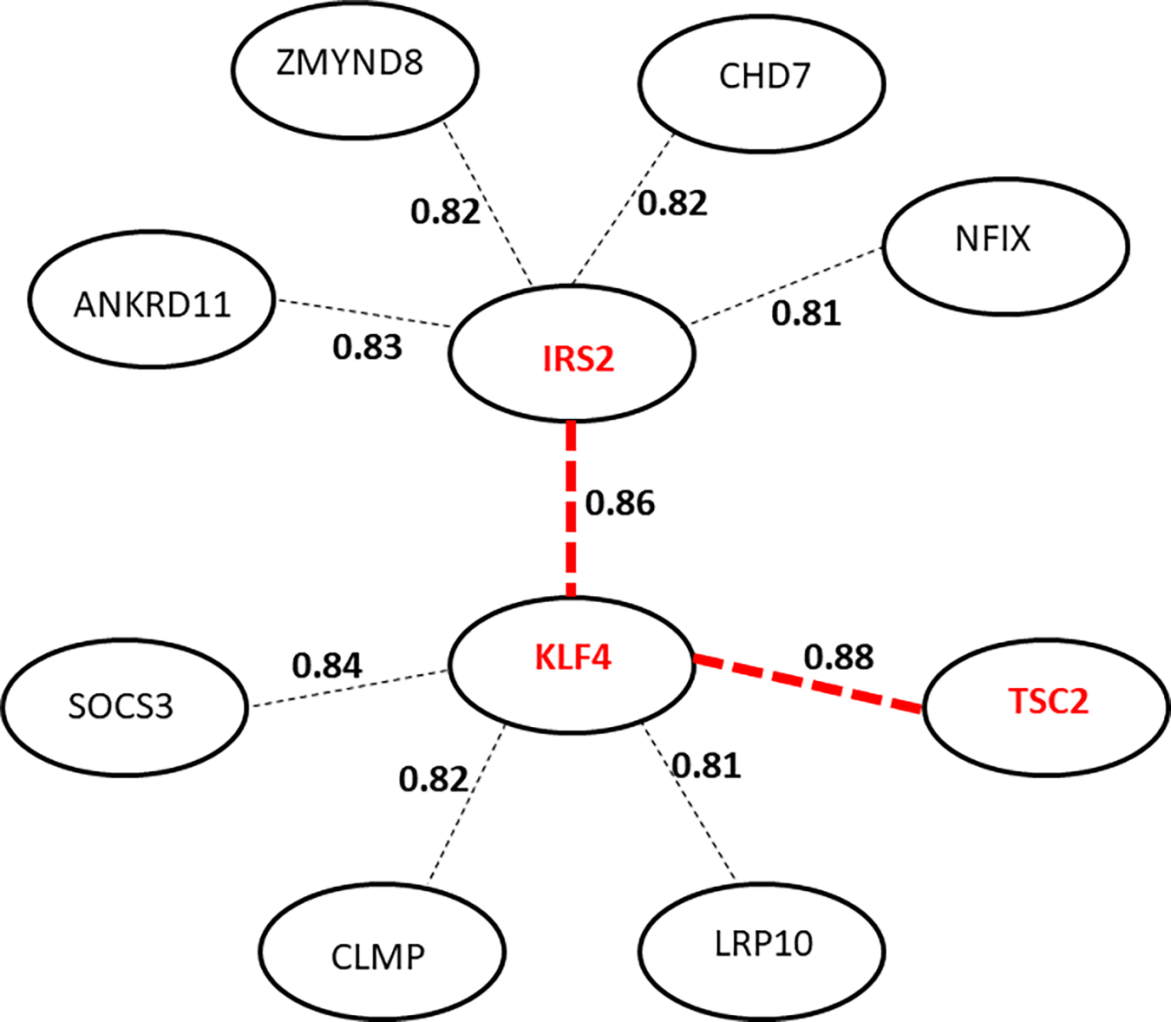
Reverse application of BAKE. Network expansion around IRS2 identified 5 neighbors (connected to IRS2 through dotted edges) that are transcriptional regulators. The degree of correlation between the gene expression profiles across the four conditions (DC8, DC16, DW8 and DW16) are shown as numbers on the edges connecting the genes. The network around KLF4, the strongest neighbor of IRS2, was similarly expanded as for IRS2 to rediscover IRS2 as one of KLF4’s neighbor along with TSC2, KLF4’s strongest neighbor. The edges connecting KLF4, IRS2 and TSC2, are highlighted in red to mark their strong associations. Note that the correlation coefficient between the expression profiles of TSC2 and IRS2 was also remarkable with 0.76 (not shown).

Among the top 20 genes most highly correlated with IRS2 we extracted five genes that a literature search identified as transcriptional regulators: KLF4, ANKRD11, ZMYND8, CHD7 and NFIX (Fig 4 and S4 Table). Among these, transcription factor KLF4 expression most highly correlated with IRS2 expression (correlation coefficient 0.86) (Fig 4). In support of this observation a strong association of KLF4 with IRS2 expression had previously been observed [43]. Furthermore, using the Ensemble database we found binding motifs for KLF4 ({G/A}{G/A}GG{C/T}G{C/T}) in the promotor sequence of IRS2 (S5 Table). Two of these were highly conserved across human, mouse and rat IRS2 promotors.

To reversely validate this novel gene’s network interaction, we next expanded the network around KLF4 using the same strategy as described above for IRS2. From this reverse BAKE application, several known insulin signaling pathway genes including IRS2 were identified as close neighbors of KLF4 (L_KLF4_; S6 Table). In particular, re-identification of IRS2 as one of the closest neighbors of KLF4 (correlation coefficient 0.86) confirmed the relationship between the two. Also among the 20 closest neighbors of KLF4 were TSC2 (correlation coefficient 0.88) and Suppressor of Cytokine Signaling 3 (SOCS3) (correlation coefficient 0.84) (Fig 4). TSC2 is also a well-known intermediate in the insulin signaling pathway [37], while SOCS3 modulates insulin signaling [44]. When analyzing the TSC2 promoter using the Ensemble database, we found one KLF4 binding motif that was reasonably conserved between human, mouse and rat (S7 Table). Upon further expansion to the 50 closest neighbors of KLF4, Coxsackie- and adenovirus receptor-Like Membrane Protein (CLMP, rank 35), a known KLF4 target gene in mouse TM4 Sertoli cells [45] was identified.

### Experimental validation of IRS2 and TSC2 regulation by KLF4

**Fig 5:**
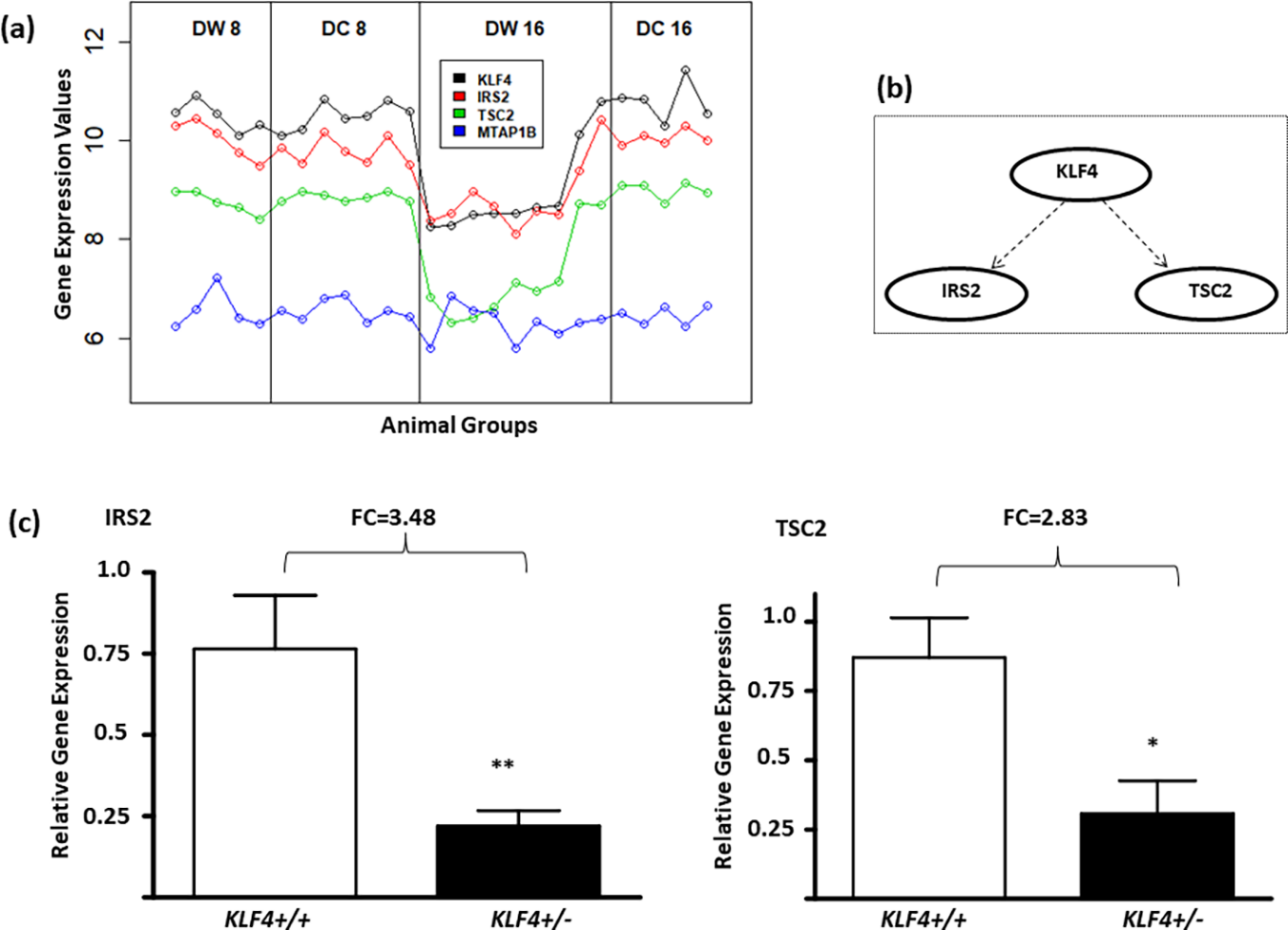
Regulation of IRS2 and TSC2 by KLF4. (a) Strong correlation of expression patterns of KLF4, IRS2 and TSC2 across different conditions (DC8, DW8, DC16, DW16), but little correlation with expression of the negative control gene, MTAP1B, across the different conditions. (b) Since KLF4 is a known transcriptional regulator and KLF4 binding motifs are present in IRS2 and TSC2 gene promoters, we conjectured that KLF4 regulates the expression of the two genes. (c) Relative mRNA expression levels (shown as means +/- SEM) of IRS2 (left panel) and TSC2 (right panel) in adipose tissues of KLF4-deficient (*KLF4*^*+/-*^, N = 4) and KLF4 *wild type* (*KLF4*^*+/+*^, N = 4) adipose tissues with fold changes (FC) as indicated. * p = 0.06 and ** p <0.05. The relative expression levels of MTAP1B mRNA were not different between *KLF4*^*+/-*^ and *KLF4*^*+/+*^ adipose tissues.

The above BAKE analysis enabled us to computationally identify KLF4 as a network gene closely correlated with IRS2 and TSC2 in the insulin signaling pathway (Fig 5A). As binding motifs for KLF4 were present in IRS2 and TSC2 promoters, we conjectured that KLF4 was a transcriptional regulator of IRS2 and TSC2 [3, 46] (Fig 5B). To experimentally corroborate this relationship, we used a conditional knockout mouse model of KLF4 (*KLF4*^*+/-*^) [33]. We found that IRS2 and TSC2 mRNAs were, in fact, downregulated 3.5 (p = 0.04) and 2.8 fold (p = 0.057), respectively, in adipose tissues of *KLF4*^*+/-*^ mice compared to KLF4 *wild-type* mice (Fig 5C). Thus, this corroborated the concept that KLF4 regulates IRS2 and TSC2 expression.

### Assessment and comparison of overall network inference accuracy by BAKE

The results from the above analysis suggested a high prediction accuracy of BAKE for biological network inference. However, it was uncertain what degree of novel real network discovery could be achieved by the BAKE approach overall. Therefore, to evaluate overall accuracy of novel gene discovery and network relationships inferred by BAKE, we reconstructed an established adipogenesis regulatory network containing 84 pathway genes represented by 225 Affimetrix probes from the literature [47–51] and Qiagen’s IPA® network analysis tool (S3 Fig). Among the pathway genes 52 genes (represented by 73 Affimetrix probes) were differentially expressed between DW16 and DC16 adipocytes at p = 0.01 (L_path adipog_ listed in S8 Table and shown in either green or red in S3 Fig). Among these 52 genes we randomly selected 20 as anchor genes (represented by 20 Affimetrix probes) (L_anchor adipog_) (shown in red in S3 Fig) and the remaining 32 genes/probes as hidden genes (L_hidden_, shown in green in S3 Fig) for our evaluation of BAKE network discovery. We then used BAKE’s sequential analysis steps to identify relationships of anchor genes with candidate genes in L_0_ (L_0_ = 1317 genes, S2 Table). To strengthen the biological information for adipogenesis network construction, we added adipocyte gene expression data from *ApoE*^*-/-*^ mice fed a Western diet for 8 and 16 weeks (EW8 (n = 6), EW16 (n = 8) and EC16 (n = 5)) to the data from *IR*^*+/-*^ *IRS*^*+/-*^ *ApoE*^*-/-*^ mice (DC8, DW8, DC16 and DW16) when determining Spearman’s rank correlation between gene expression profiles. Genes that were identified as novel after the application of the BAKE steps described above are listed in S9 Table. Six of these were hidden genes (L_hidden_, shown in bold in S9 Table) for which relationships to anchor genes were correctly inferred. We thus designated these as True Positives (TP). The remainder were designated False Positives (FP). Sensitivity, specificity and positive predictive values (PPV) for discovering relevant novel genes were then calculated (Table 1).

**Table 1:** Comparison of performance of BAKE and GP-DREAM in adipogenesis network construction.

Performance Measure	Discovery of Novel Genes ^a^	
	**BAKE**	**GP-DREAM**
Sensitivity^b^	11%	34%
Specificity^b^	98%	50%
PPV^b^	16%	2%

^a^ Novel genes present in the hidden gene list (L_hidden_) were classified as True Positives (TP) while those that were not among the hidden genes were designated False Positives (FP). False Negatives (FNs) were those hidden genes that were not successfully discovered and True Negatives (TNs) were those genes correctly not discovered because they were not in the hidden gene list.

^b^Sensitivity = TP/(TP+FN), Specificity = TN/(TN+FP) and Positive Predictive Value (PPV) = TP/(TP+FP).

Note that the performance values in Table 1 are conservative estimates of performance as some of the novel genes discovered or gene interactions classified as false positives may be relevant to the network but are not yet supported by the literature. For example, we further examined 45 false positive gene relationships between two anchor genes and a novel gene that were discovered by BAKE (S10 Table). Literature evidence and molecular interaction databases were used to classify the interactions as True Positives (TPs, regulatory interactions between genes supported by the literature, 22 gene interactions), False Positives (FPs, regulatory interactions not supported by literature, 23 gene interactions) (S10 Table). The accuracy in terms of PPV of BAKE in inferring gene relationships could then be improved to 49% or higher (= 22/45).

We then also compared the performance of our adipogenesis network reconstruction inferred by BAKE with the most widely used and highly cited Network Inference (NI) approaches, the Gene Pattern-Dialogue on Reverse Engineering Assessment and Methods (GP-DREAM) [22, 24]. GP-DREAM is a public platform that was formerly freely available for application of network inference methods on gene expression data and is maintained by DREAM challenge organizers and Gene Patten team at the Broad Institute. GP-DREAM utilizes assembled information from multiple NI techniques including the Context Likelihood of Relatedness (CLR, ranking gene interactions by estimating Mutual Information (MI) between genes, [5]), Correlation (ranking gene relationships based on the degree of correlation in their expression patterns, [22]), and GEne Network Inference with Ensemble of trees (GENIE3, expression profile of each novel gene is predicted from the expression profiles of anchor genes using a tree based ensemble method and the gene relationships are ranked based on the importance of anchor genes for the prediction (19)). When applying GP-DREAM to the 84-gene adipogenesis network, ranks for candidate gene relationships from these methods were integrated by averaging them for further analysis. Unlike BAKE, GP-DREAM did not directly provide a specific number of candidate networks for further investigation. When all candidate networks identified by the GP-DREAM were considered, it showed a very high false positive error rate of 50% (compared to 2% by BAKE) and very low PPV of 2% (compared to 16% by BAKE). The performance of GP-DREAM after combining the three NI approaches is also summarized in Table 1. As examined with additional literature information for BAKE, if PPV of gene relationship inference by GP-DREAM was assessed similarly with the top 30, 40 and 50 candidate networks, it yielded PPV of 40%, 35%, and 42%, respectively. Therefore, the BAKE approach with PPV>49% still outperformed the ensembled network inference approach by GP-DREAM with a significantly lower rate of false positives.

## Discussion

Different computational network mining and modeling approaches have been developed in recent years to reconstruct complex biological networks from high throughput molecular data in genomics, proteomics, metabolomics and other omics-based studies. In *silico* network mining techniques, however, often identify numerous false network interactions that can only be conclusively uncovered by performing extensive biological experiments. Some recent computational network inference techniques may yield as high as 98% false positives when identifying novel gene interactions in an adipogenesis network (**Table 1**). To overcome this critical shortcoming, we have developed BAKE, a new bioinformatics investigation strategy that is based on interactive sequential integration of known biological information and computational *in silico* analysis. Specifically, we have formalized an approach that researchers often already use to effectively investigate and validate their omics data-based discovery as five sequential BAKE analysis steps: *in silico* search → literature mining → integration and association of *in silico* search and literature mining → *in silico* network expansion around known network genes → *in silico* reverse-confirmation and use of literature and data resources for confirmation. Note that BAKE is an omics-based network inference approach rather than a fixed algorithm or software. Nevertheless, the full use and order of the five BAKE interactive and integrative analysis steps, combining computational discovery and known information, is important. In earlier BAKE attempts, we omitted some of these analysis steps and found that performance was significantly worse. For instance, gene clustering before biological knowledge anchoring resulted in numerous false positive gene clusters.

As we demonstrated, the implementation of the sequential BAKE analysis steps dramatically increased the likelihood of identifying true positives. When applying BAKE to an experimental animal model of insulin resistance progression, we discovered a novel regulatory transcription factor KLF4 to interact with IRS2 and TSC2, two key intermediates in the insulin signaling pathway. We were then able to experimentally corroborate a role for KLF4 in regulating IRS2 and TSC2 expression using KLF4 knockout mice. These investigations suggest that reduced expression of KLF4 triggered by a high-fat diet in our mouse model (*IR*^*+/-*^*IRS1*^*+/-*^*ApoE*^*-/-*^) may mediate down-regulation of IRS2 and TSC2 expression thereby impairing insulin signaling in adipocytes and promoting insulin resistance. To our knowledge, this is the first study to report the expression regulation of KLF4 on the key intermediates in the insulin signaling pathway, so a further investigation will be of high interest for its biological mechanisms and regulation.

One of the key features of BAKE was the use of SPC algorithm. The ability of SPC algorithm to naturally deduce gene relationships as well as their strength and closeness through the algorithm’s temperature gradient without much intervention from the user was an attractive feature that led us to use it in BAKE. One of the questions that arose while implementing SPC algorithm in BAKE (Step 4, Expansion of networks around known network genes) was to decide on the number of anchor and novel genes to be clustered by SPC. While it is difficult to define an exact number of anchor genes, we empirically found that our SPC and other algorithms we used perform well when the total number of both anchor and novel genes was around 40 (that is, L_neighbor_ + L_anchor_ = 40). Therefore, the number of novel genes (L_neighbor_) that can be effectively introduced and computationally investigated with the known network genes is dependent on the number of anchor genes. For example, SPC with ~40 genes at K (number of nearest neighbors for clustering) = 10 generally produced stable clusters of 3 to 5 genes, which is an ideal size to study interactions between anchor and novel genes. If the total number was larger or smaller, the number or size of clusters tended to be too large or too small. BAKE/SPC algorithm is not directly sensitive to the number of genes in L_anchor_ but we used this empirical guideline for defining the number of novel genes to be investigated and suggest to keep L_anchor_ = L_neighbor_ = 20. This helps to generate more clusters involving anchor and novel genes, ideal for network expansion around known network genes in Step 4 of BAKE.

BAKE also has limitations in the current form. We assumed that two genes were functionally associated if their expressions were correlated across different conditions. Since we removed overall mean differences among different conditions by quantile normalization, remaining associations would be largely due to biological causes rather than experimental artifacts. However, this may not completely remove indirect biological associations between genes such as epiphenomenal effects. Also, some BAKE steps depend on researchers’ decisions, including the choice of relevant high throughput molecular data representing biological conditions of interest and the selection of prior knowledge in pathways of interest. Prior knowledge in turn is dependent on the reliability of information in the literature and public genomic databases used. Nevertheless, to make omics-based biological investigations practical, these may generally need to be restricted by subjective directions of interest. Our BAKE approach, which we frame as “objective use of subjective information,” enables a systematic search for novel network genes using prior knowledge of biological mechanisms of interest. Another limitation of BAKE is that it uses static gene expression data, and thus cannot resolve direction of edges in the network. We plan to use dynamic information from high throughput molecular data in future gene network inference. Finally, BAKE cannot infer complex gene regulatory interactions such as feedback loops and synergistic gene regulations as it relies on a one-dimensional association search for novel network genes. Further improvements will be required to overcome such limitations.

## Supporting information

S1 Fig Determination of the optimal correlation threshold for selection of L_1_ from L_0_. Comparison of correlation coefficients of L_0_ genes with insulin signaling pathway genes (L_path_) and non-pathway genes (L_pathrandom_) was repeated 100 times (with L_pathrandom_ also randomly sampled 100 times) using Response Operator Characteristics (ROC) curves. Youden’s J Index was estimated for the 100 comparisons in the ROC curve and the median correlation threshold for maximum Youden’s J Index was estimated as 0.72. A more detailed description is provided under step 3 of BAKE in Materials and Methods.
(PDF)

S2 Fig Temperature profile of clusters generated by BAKE at higher resolution. The central part of the temperature profile shown in Fig 2B bottom panel was magnified. The circled clusters (same as in Fig 2B) are the most stable clusters surviving higher temperatures. Anchor genes within the clusters are highlighted in bold.
(PDF)

S3 Fig Adipogenesis network. The adipogenesis network was constructed based on literature [47–51] and Qiagen’s IPA^®^ network analysis tool. Significant pathway genes (L_path adipog_, 52 genes (73 probes)) are shown in green or red. Anchor genes (L_anchor adipog_ = 20 genes (20 probes)) are shown in red and hidden genes (L_hidden_ = 40 genes (53 probes)) in green. Eight genes were represented by different probes present in both the anchor and hidden gene list.
(PDF)

S4 Fig Determination of T_critical_ for cluster selection using anchor gene IRS2. Maximum cluster sizes at each temperature (shown as mean +/- SD estimated from N = 100 simulations of SPC clustering) were compared between temperature profiles of clusters obtained with observed gene expression data (red) and random gene expression data (green). T_critical_ (0.11 for the data shown) was defined as the lowest temperature at which maximum of cluster sizes was significantly higher for observed over random expression data or, in other words, the temperature beyond which the clustering pattern between random and observed gene expression data was distinct.
(PDF)

S1 Table Mouse phenotype data. Parameters were determined in random-fed mice before or after euthanasia as described in Materials and Methods. Data are given as means +/- SEM.
(PDF)

S2 Table L_0_ and L_1_ genes. L_0_ represents genes that were differentially expressed between DW16 and DC16 adipocytes. L_1_ represents genes in L_0_ for which expression profiles significantly correlated with expression of insulin signaling pathway genes (L_path_) in adipocytes using data for all four conditions DC8, DW8, DC16 and DW16 (marked L_1_ in table).
(PDF)

S3 Table Insulin signaling pathway (L_path_) genes and anchor genes (L_anchor_). L_anchor_ represents genes in L_path_ that were differentially expressed in adipocytes between DW16 and DC16 (marked L_anchor_ in table). Fold Changes (FC) in gene expression between DW16 and DC16 are given in logarithmic scale (base 2).
(PDF)

S4 Table Twenty novel neighbor genes around anchor gene IRS2 (L_IRS2_). Fold Changes (FC) in expression of neighbor genes between DW16 and DC16 are given in logarithmic scale (base 2).
(PDF)

S5 Table IRS2 promoter analysis. Nucleotide sequences 10 kb upstream of initiation start site ATG were scanned for KLF4 binding motifs ({G/A}{G/A}GG{C/T}G{C/T}) and the positions of motifs compared between human, mouse and rat promoters. Motifs shown in red were considered conserved based on the following criteria. They were found in the promoter of all three species, and located no more than 100 bases of each other across the different species and no more than ~ 1000 bases from the start site. Motifs that were less conserved across the three species due to single base variations are shown in green. Positions in promoters are given relative to translation start sites.
(PDF)

S6 Table Twenty novel neighbor genes around anchor gene KLF4 (L_KLF4_). Fold Changes (FC) in expression of neighbor genes between DW16 and DC16 are given in logarithmic scale (base 2).
(PDF)

S7 Table TSC2 promoter analysis. Promoter regions of human, mouse and rat TSC2 were analyzed as described in legend to Suppl. Table S5. Positions in promoters are given relative to translation start sites.
(PDF)

S8 Table List of differentially expressed adipogenesis network and anchor genes. List of differentially expressed adipogenesis network genes (L_path adipog_, 52 genes represented by 73 Affymetrix probes) between DW16 and DC16 (p<0.01) are shown with fold changes in expression (FC in logarithmic scale to base 2). Among these, 20 genes (represented by 20 Affymetrix probes) were randomly selected as anchor genes (L_anchor adipog_). The remaining genes formed the list of hidden genes (L_hidden_) to be discovered by BAKE through network expansion around anchor genes.
(PDF)

S9 Table Novel genes discovered by BAKE during adipogenesis gene network expansion. Genes are listed in alphabetical order. Hidden adipogenesis network genes discovered among novel genes are in bold.
(PDF)

S10 Table Gene interactions inferred by BAKE during adipogenesis network expansion. Anchor gene-anchor gene and anchor gene-hidden gene interactions inferred by BAKE are shown along with their classification as True Positives (TPs) and False Positives (FPs). Literature evidence for TP gene interactions were obtained from the adipogenesis network shown in S3 Fig or the network analysis tool Ingenuity Pathway Analysis (IPA^®^). In IPA gene interactions are derived and curated from a variety of databases such as Ingenuity Expert Information, microRNA-mRNA interaction database (miRecords), protein-protein interaction databases (including BIND, Cognia, MIPS), BioGRID, Gene Ontology (GO), Online Mendelian Inheritance in Man (OMIM) and Mouse Genome Database (MGD).
(PDF)
