# Correction: Biologically anchored knowledge expansion approach uncovers *KLF4* as a novel insulin signaling regulator

**DOI:** 10.1371/journal.pone.0207325

**Published:** 2018-11-07

**Authors:** Annamalai Muthiah, Morgan S. Angulo, Natalie N. Walker, Susanna R. Keller, Jae K. Lee

S2 Table is incomplete. The bottom part of S2 Table is missing. The full S2 Table can be viewed below.

## Supporting information

S2 Table L_0_ and L_1_ genes. L_0_ represents genes that were differentially expressed between DW16 and DC16 adipocytes. L_1_ represents genes in L_0_ for which expression profiles significantly correlated with expression of insulin signaling pathway genes (L_path_) in adipocytes using data for all four conditions DC8, DW8, DC16 and DW16 (marked L_1_ in table).
(PDF)
