# Supplementary figures and images for: Biologically anchored knowledge expansion approach uncovers KLF4 as a novel insulin signaling regulator
Source: PLoS One. 2018 Sep 21;13(9):e0204100. doi: 10.1371/journal.pone.0204100 (PMC6150497; doi:10.1371/journal.pone.0204100)

**S1 Fig**

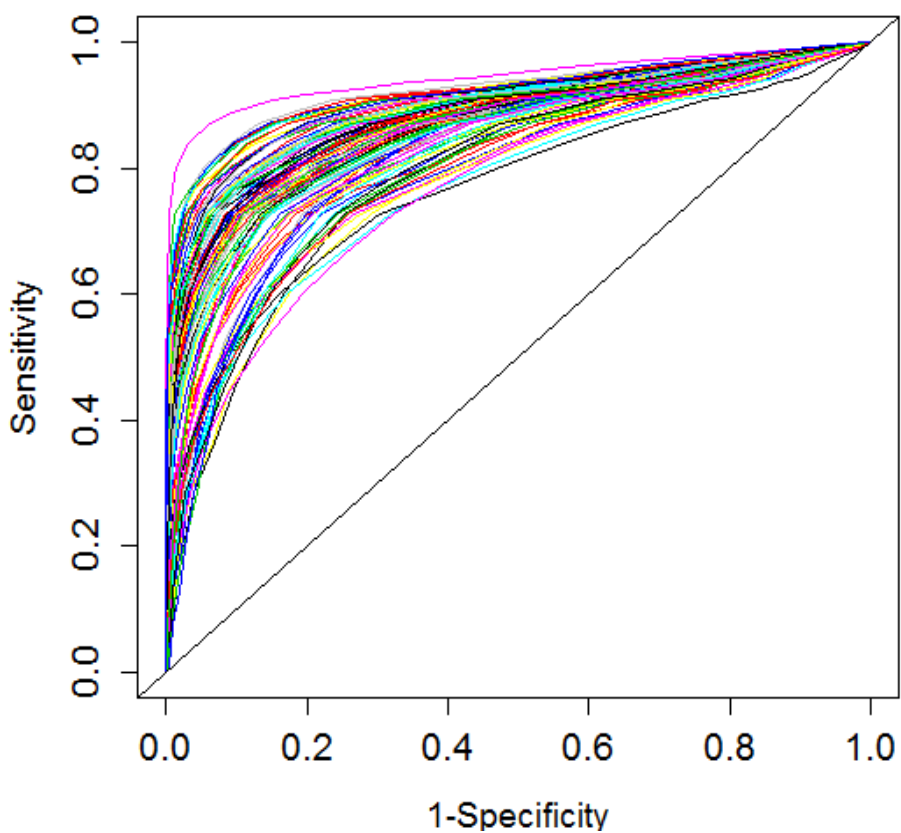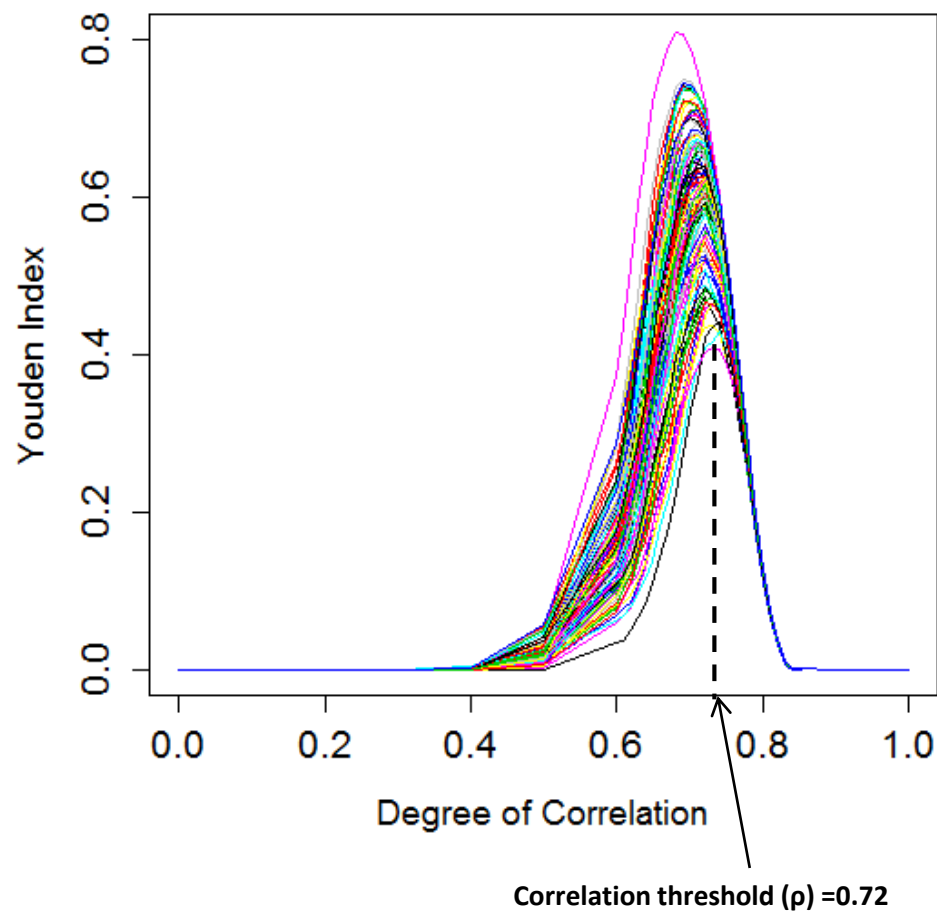

Supplement: S1 Fig — Comparison of correlation coefficients of L0 genes with insulin signaling pathway genes (Lpath) and non-pathway genes (Lpathrandom) was repeated 100 times (with Lpathrandom also randomly sampled 100 times) using Response Operator Characteristics (ROC) curves. Youden’s J Index was estimated for the 100 comparisons in the ROC curve and the median correlation threshold for maximum Youden’s J Index was estimated as 0.72. A more detailed description is provided under step 3 of BAKE in Materials and Methods. (PDF) [file pone.0204100.s001.pdf]

S3 Fig

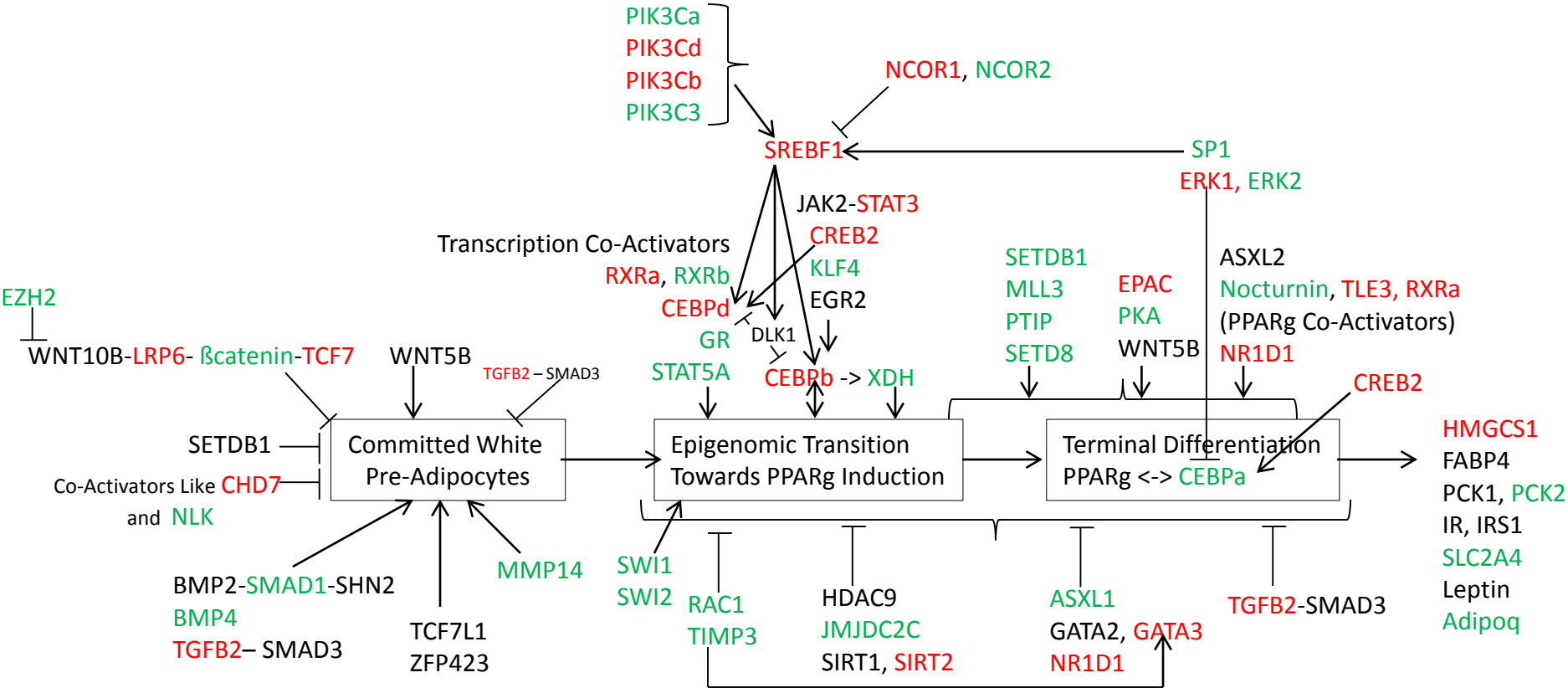

Supplement: S3 Fig — The adipogenesis network was constructed based on literature [47–51] and Qiagen’s IPA® network analysis tool. Significant pathway genes (Lpath adipog, 52 genes (73 probes)) are shown in green or red. Anchor genes (Lanchor adipog = 20 genes (20 probes)) are shown in red and hidden genes (Lhidden = 40 genes (53 probes)) in green. Eight genes were represented by different probes present in both the anchor and hidden gene list. (PDF) [file pone.0204100.s003.pdf]

S4 Fig

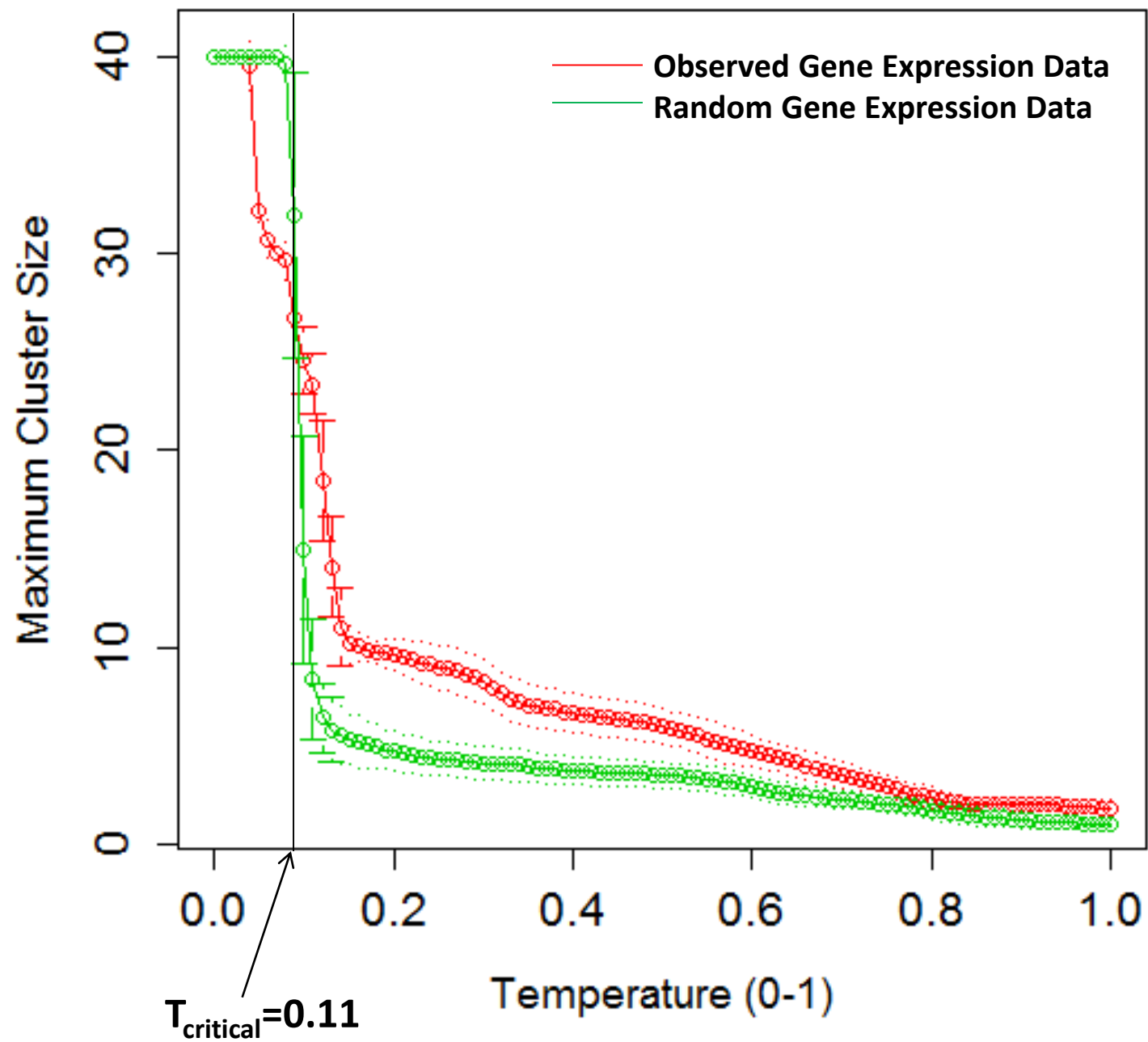

Supplement: S4 Fig — Maximum cluster sizes at each temperature (shown as mean +/- SD estimated from N = 100 simulations of SPC clustering) were compared between temperature profiles of clusters obtained with observed gene expression data (red) and random gene expression data (green). Tcritical (0.11 for the data shown) was defined as the lowest temperature at which maximum of cluster sizes was significantly higher for observed over random expression data or, in other words, the temperature beyond which the clustering pattern between random and observed gene expression data was distinct. (PDF) [file pone.0204100.s004.pdf]
