# Supplementary material for: Biologically anchored knowledge expansion approach uncovers KLF4 as a novel insulin signaling regulator
Source: PLoS One. 2018 Sep 21;13(9):e0204100. doi: 10.1371/journal.pone.0204100 (PMC6150497; doi:10.1371/journal.pone.0204100)
Supplement: S2 Fig — The central part of the temperature profile shown in Fig 2B bottom panel was magnified. The circled clusters (same as in Fig 2B) are the most stable clusters surviving higher temperatures. Anchor genes within the clusters are highlighted in bold. (PDF) [file pone.0204100.s002.pdf]

S2 Fig

GYS1	GYS1	GYS1	GYS1	GYS1	GYS1	GYS1	GYS1	GYS1	GYS1	GYS1	GYS1	GYS1	GYS1	GYS1	GYS1	GYS1	GYS1	GYS1	GYS1
AKT1	AKT1	AKT1	AKT1	AKT1	AKT1	AKT1	AKT1	AKT1	AKT1	AKT1	AKT1	AKT1	AKT1	AKT1	AKT1	AKT1	AKT1	AKT1	AKT1
Raptor	Raptor	Raptor	Raptor	Raptor	Raptor	Raptor	Raptor	Raptor	Raptor	Raptor	Raptor	Raptor	Raptor	Raptor	Raptor	Raptor	Raptor	Raptor	Raptor
SHP2	SHP2	SHP2	SHP2	SHP2	SHP2	SHP2	SHP2	SHP2	SHP2	SHP2	SHP2	SHP2	SHP2	SHP2	SHP2	SHP2	SHP2	SHP2	SHP2
Rictor	Rictor	Rictor	Rictor	Rictor	Rictor	Rictor	Rictor	Rictor	Rictor	Rictor	Rictor	Rictor	Rictor	Rictor	Rictor	Rictor	Rictor	Rictor	Rictor
AKT1	AKT1	AKT1	AKT1	AKT1	AKT1	AKT1	AKT1	AKT1	AKT1	AKT1	AKT1	AKT1	AKT1	AKT1	AKT1	AKT1	AKT1	AKT1	AKT1
GYS1	GYS1	GYS1	GYS1	GYS1	GYS1	GYS1	GYS1	GYS1	GYS1	GYS1	GYS1	GYS1	GYS1	GYS1	GYS1	GYS1	GYS1	GYS1	GYS1
CHD7	CHD7	CHD7	CHD7	CHD7	CHD7	CHD7	CHD7	CHD7	CHD7	CHD7	CHD7	CHD7	CHD7	CHD7	CHD7	CHD7	CHD7	CHD7	CHD7
IRS2	IRS2	IRS2	IRS2	IRS2	IRS2	IRS2	IRS2	IRS2	IRS2	IRS2	IRS2	IRS2	IRS2	IRS2	IRS2	IRS2	IRS2	IRS2	IRS2
KLF4	KLF4	KLF4	KLF4	KLF4	KLF4	KLF4	KLF4	KLF4	KLF4	KLF4	KLF4	KLF4	KLF4	KLF4	KLF4	KLF4	KLF4	KLF4	KLF4
AKT2	AKT2	AKT2	AKT2	AKT2	AKT2	AKT2	AKT2	AKT2	AKT2	AKT2	AKT2	AKT2	AKT2	AKT2	AKT2	AKT2	AKT2	AKT2	AKT2
Rab10a	Rab10a	Rab10a	Rab10a	Rab10a	Rab10a	Rab10a	Rab10a	Rab10a	Rab10a	Rab10a	Rab10a	Rab10a	Rab10a	Rab10a	Rab10a	Rab10a	Rab10a	Rab10a	Rab10a
p110Beta	p110Beta	p110Beta	p110Beta	p110Beta	p110Beta	p110Beta	p110Beta	p110Beta	p110Beta	p110Beta	p110Beta	p110Beta	p110Beta	p110Beta	p110Beta	p110Beta	p110Beta	p110Beta	p110Beta
4921504P05Rik	4921504P05Rik	4921504P05Rik	4921504P05Rik	4921504P05Rik	4921504P05Rik	4921504P05Rik	4921504P05Rik	4921504P05Rik	4921504P05Rik	4921504P05Rik	4921504P05Rik	4921504P05Rik	4921504P05Rik	4921504P05Rik	4921504P05Rik	4921504P05Rik	4921504P05Rik	4921504P05Rik	4921504P05Rik
ZMYND8	ZMYND8	ZMYND8	ZMYND8	ZMYND8	ZMYND8	ZMYND8	ZMYND8	ZMYND8	ZMYND8	ZMYND8	ZMYND8	ZMYND8	ZMYND8	ZMYND8	ZMYND8	ZMYND8	ZMYND8	ZMYND8	ZMYND8
PP2AC	PP2AC	PP2AC	PP2AC	PP2AC	PP2AC	PP2AC	PP2AC	PP2AC	PP2AC	PP2AC	PP2AC	PP2AC	PP2AC	PP2AC	PP2AC	PP2AC	PP2AC	PP2AC	PP2AC
SHIP2	SHIP2	SHIP2	SHIP2	SHIP2	SHIP2	SHIP2	SHIP2	SHIP2	SHIP2	SHIP2	SHIP2	SHIP2	SHIP2	SHIP2	SHIP2	SHIP2	SHIP2	SHIP2	SHIP2
TNK2	TNK2	TNK2	TNK2	TNK2	TNK2	TNK2	TNK2	TNK2	TNK2	TNK2	TNK2	TNK2	TNK2	TNK2	TNK2	TNK2	TNK2	TNK2	TNK2
SKIV2L	SKIV2L	SKIV2L	SKIV2L	SKIV2L	SKIV2L	SKIV2L	SKIV2L	SKIV2L	SKIV2L	SKIV2L	SKIV2L	SKIV2L	SKIV2L	SKIV2L	SKIV2L	SKIV2L	SKIV2L	SKIV2L	SKIV2L
IRAK1	IRAK1	IRAK1	IRAK1	IRAK1	IRAK1	IRAK1	IRAK1	IRAK1	IRAK1	IRAK1	IRAK1	IRAK1	IRAK1	IRAK1	IRAK1	IRAK1	IRAK1	IRAK1	IRAK1
BOD1L	BOD1L	BOD1L	BOD1L	BOD1L	BOD1L	BOD1L	BOD1L	BOD1L	BOD1L	BOD1L	BOD1L	BOD1L	BOD1L	BOD1L	BOD1L	BOD1L	BOD1L	BOD1L	BOD1L
FRAP1	FRAP1	FRAP1	FRAP1	FRAP1	FRAP1	FRAP1	FRAP1	FRAP1	FRAP1	FRAP1	FRAP1	FRAP1	FRAP1	FRAP1	FRAP1	FRAP1	FRAP1	FRAP1	FRAP1
KIAA0494	KIAA0494	KIAA0494	KIAA0494	KIAA0494	KIAA0494	KIAA0494	KIAA0494	KIAA0494	KIAA0494	KIAA0494	KIAA0494	KIAA0494	KIAA0494	KIAA0494	KIAA0494	KIAA0494	KIAA0494	KIAA0494	KIAA0494
Glut4	Glut4	Glut4	Glut4	Glut4	Glut4	Glut4	Glut4	Glut4	Glut4	Glut4	Glut4	Glut4	Glut4	Glut4	Glut4	Glut4	Glut4	Glut4	Glut4
Glut4	Glut4	Glut4	Glut4	Glut4	Glut4	Glut4	Glut4	Glut4	Glut4	Glut4	Glut4	Glut4	Glut4	Glut4	Glut4	Glut4	Glut4	Glut4	Glut4
PCNX	PCNX	PCNX	PCNX	PCNX	PCNX	PCNX	PCNX	PCNX	PCNX	PCNX	PCNX	PCNX	PCNX	PCNX	PCNX	PCNX	PCNX	PCNX	PCNX
RAB11FIP3	RAB11FIP3	RAB11FIP3	RAB11FIP3	RAB11FIP3	RAB11FIP3	RAB11FIP3	RAB11FIP3	RAB11FIP3	RAB11FIP3	RAB11FIP3	RAB11FIP3	RAB11FIP3	RAB11FIP3	RAB11FIP3	RAB11FIP3	RAB11FIP3	RAB11FIP3	RAB11FIP3	RAB11FIP3
MAPK8IP3	MAPK8IP3	MAPK8IP3	MAPK8IP3	MAPK8IP3	MAPK8IP3	MAPK8IP3	MAPK8IP3	MAPK8IP3	MAPK8IP3	MAPK8IP3	MAPK8IP3	MAPK8IP3	MAPK8IP3	MAPK8IP3	MAPK8IP3	MAPK8IP3	MAPK8IP3	MAPK8IP3	MAPK8IP3
ZNF281	ZNF281	ZNF281	ZNF281	ZNF281	ZNF281	ZNF281	ZNF281	ZNF281	ZNF281	ZNF281	ZNF281	ZNF281	ZNF281	ZNF281	ZNF281	ZNF281	ZNF281	ZNF281	ZNF281
Tnfrsf1a	Tnfrsf1a	Tnfrsf1a	Tnfrsf1a	Tnfrsf1a	Tnfrsf1a	Tnfrsf1a	Tnfrsf1a	Tnfrsf1a	Tnfrsf1a	Tnfrsf1a	Tnfrsf1a	Tnfrsf1a	Tnfrsf1a	Tnfrsf1a	Tnfrsf1a	Tnfrsf1a	Tnfrsf1a	Tnfrsf1a	Tnfrsf1a
SLCO2B1	SLCO2B1	SLCO2B1	SLCO2B1	SLCO2B1	SLCO2B1	SLCO2B1	SLCO2B1	SLCO2B1	SLCO2B1	SLCO2B1	SLCO2B1	SLCO2B1	SLCO2B1	SLCO2B1	SLCO2B1	SLCO2B1	SLCO2B1	SLCO2B1	SLCO2B1
SPSB1	SPSB1	SPSB1	SPSB1	SPSB1	SPSB1	SPSB1	SPSB1	SPSB1	SPSB1	SPSB1	SPSB1	SPSB1	SPSB1	SPSB1	SPSB1	SPSB1	SPSB1	SPSB1	SPSB1
HIPK3	HIPK3	HIPK3	HIPK3	HIPK3	HIPK3	HIPK3	HIPK3	HIPK3	HIPK3	HIPK3	HIPK3	HIPK3	HIPK3	HIPK3	HIPK3	HIPK3	HIPK3	HIPK3	HIPK3
SOCS3	SOCS3	SOCS3	SOCS3	SOCS3	SOCS3	SOCS3	SOCS3	SOCS3	SOCS3	SOCS3	SOCS3	SOCS3	SOCS3	SOCS3	SOCS3	SOCS3	SOCS3	SOCS3	SOCS3
SOCS3	SOCS3	SOCS3	SOCS3	SOCS3	SOCS3	SOCS3	SOCS3	SOCS3	SOCS3	SOCS3	SOCS3	SOCS3	SOCS3	SOCS3	SOCS3	SOCS3	SOCS3	SOCS3	SOCS3
SOCS3	SOCS3	SOCS3	SOCS3	SOCS3	SOCS3	SOCS3	SOCS3	SOCS3	SOCS3	SOCS3	SOCS3	SOCS3	SOCS3	SOCS3	SOCS3	SOCS3	SOCS3	SOCS3	SOCS3
ANKRD11	ANKRD11	ANKRD11	ANKRD11	ANKRD11	ANKRD11	ANKRD11	ANKRD11	ANKRD11	ANKRD11	ANKRD11	ANKRD11	ANKRD11	ANKRD11	ANKRD11	ANKRD11	ANKRD11	ANKRD11	ANKRD11	ANKRD11
NFIX	NFIX	NFIX	NFIX	NFIX	NFIX	NFIX	NFIX	NFIX	NFIX	NFIX	NFIX	NFIX	NFIX	NFIX	NFIX	NFIX	NFIX	NFIX	NFIX
NFIX	NFIX	NFIX	NFIX	NFIX	NFIX	NFIX	NFIX	NFIX	NFIX	NFIX	NFIX	NFIX	NFIX	NFIX	NFIX	NFIX	NFIX	NFIX	NFIX
