# Supplementary material for: Biologically anchored knowledge expansion approach uncovers KLF4 as a novel insulin signaling regulator
Source: PLoS One. 2018 Sep 21;13(9):e0204100. doi: 10.1371/journal.pone.0204100 (PMC6150497; doi:10.1371/journal.pone.0204100)
Supplement: S1 Table — Parameters were determined in random-fed mice before or after euthanasia as described in Materials and Methods. Data are given as means +/- SEM. (PDF) [file pone.0204100.s005.pdf]

S1 Table. Mouse Phenotype Data

Animal Group	Blood Glucose (mg/dL)	Serum Insulin (ng/mL)	Adipose Tissue Weight/Body Weight (%)	Body Weight (g)
DW8 (n=5)	181 +/- 6.9	3.25 +/- 0.65	1.57 +/- 0.12	20.9 +/- 0.37
DC8 (n=7)	178 +/- 4.8	3.03 +/- 0.63	1.3 +/- 0.07	19.1 +/- 0.44
DW16 (n=9)	187 +/- 10.2	2.36 +/- 0.77	1.63 +/- 0.17	21.9 +/- 0.67
DC16 (n=5)	172+/- 12.1	1.96 +/- 0.39	1.16 +/- 0.08	21.0 +/- 0.84
