# Supplementary material for: Biologically anchored knowledge expansion approach uncovers KLF4 as a novel insulin signaling regulator
Source: PLoS One. 2018 Sep 21;13(9):e0204100. doi: 10.1371/journal.pone.0204100 (PMC6150497; doi:10.1371/journal.pone.0204100)
Supplement: S3 Table — Lanchor represents genes in Lpath that were differentially expressed in adipocytes between DW16 and DC16 (marked Lanchor in table). Fold Changes (FC) in gene expression between DW16 and DC16 are given in logarithmic scale (base 2). (PDF) [file pone.0204100.s007.pdf]

S3 Table. Insulin Signaling Pathway Genes (L_{path}) and Anchor Genes (L_{anchor}).

L_{path} Genes			
Probe Set ID	Gene Symbol	Log_2FC	L_{anchor} Genes
1416657_at	Akt1	-1.44408	L_{anchor}
1425711_a_at	Akt1	-1.3978	L_{anchor}
1424480_s_at	Akt2	-1.60568	L_{anchor}
1421324_a_at	Akt2	-0.56584	
1455703_at	Akt2	-0.07078	
1426239_s_at	Arrb2	-0.38043	
1451987_at	Arrb2	-0.74716	
1417592_at	FRAP1	-0.76872	L_{anchor}
1436267_a_at	FRAP1	-0.38295	
1415958_at	glut4	-1.52396	L_{anchor}
1415959_at	glut4	-2.30059	L_{anchor}
1435638_at	Gsk3a	0.042993	
1434439_at	Gsk3b	0.425375	
1437001_at	Gsk3b	0.039469	
1451020_at	Gsk3b	0.063825	
1454958_at	Gsk3b	-0.18383	
1450196_s_at	GYS1	-0.88146	L_{anchor}
1416737_at	GYS1	-1.28798	L_{anchor}
1422447_at	Ins1	0.027214	
1422446_x_at	Ins2	0.195037	
1434446_at	IR A/B	-0.00117	
1423104_at	IRS1	-0.86892	
1443969_at	IRS2	-1.43486	L_{anchor}
1437672_at	IRS3	-0.24064	
1420931_at	JNK1	-0.19766	
1421876_at	JNK2	-1.41937	
1417273_at	pdk4	0.061052	
1416501_at	Pdpk1 (PDK1)	-0.40082	
1415729_at	Pdpk1(PDK1)	0.97709	
1426994_at	Phlpp1	-0.33884	
1444593_at	PHLPP2	0.2107	
1435601_at	PHLPP2	0.026679	
1453069_at	Pik3cb(P110 beta)	-1.02557	L_{anchor}
1425514_at	Pik3r1	0.175572	
1438682_at	Pik3r1	0.149941	

1451737_at	Pik3r1	-0.34413	
1418463_at	Pik3r2	-0.67342	
1426044_a_at	PKC-theta	0.221952	
1417367_at	pp2ac	-1.01022	L_{anchor}
1456390_at	pp2ac	-0.34157	
1422553_at	Pten	-0.31217	
1450655_at	Pten	-1.18513	
1454722_at	Pten	-0.6018	
1455728_at	Pten	0.068324	
1457493_at	Pten	0.249687	
1417068_a_at	PTP 1B	-0.35068	
1438670_at	PTP 1B	-0.30576	
1453095_at	Rab10	-1.19224	L_{anchor}
1422664_at	Rab10	-0.31038	
1429296_at	Rab10	-0.86471	
1457624_at	Rab8a	0.038289	
1440620_at	Rab8a	0.687193	
1434619_at	Raptor	-1.0083	L_{anchor}
1430784_a_at	Raptor	-0.09409	
1458472_at	Raptor	0.344551	
1449182_at	Retn	-0.10437	
1427583_at	Rictor	0.413408	L_{anchor}
1435698_at	Rictor	0.20103	
1441753_at	Rictor	-0.49329	
1453775_at	Rictor	-0.01628	
1429810_at	Rictor	1.335436	
1460394_a_at	SHIP2	-1.17949	L_{anchor}
1427699_a_at	SHP2 (ptpn11)	1.692462	L_{anchor}
1451225_at	SHP2(ptpn11)	-0.40662	
1421196_at	SHP2(ptpn11)	-0.00081	
1440047_at	Socs1	-0.19449	
1450446_a_at	Socs1	-0.43768	
1416576_at	Socs3	-2.42518	L_{anchor}
1455899_x_at	Socs3	-1.248	L_{anchor}
1456212_x_at	Socs3	-1.5813	L_{anchor}
1419448_at	Tbc1d1	0.382683	
1447016_at	Tbc1d1	0.604145	
1435292_at	Tbc1d4	-1.36206	
1455903_at	Tbc1d4	-0.56784	
1419607_at	Tnfalpha	-0.58929	

1417291_at	Tnfrsf1a	-1.24859	L_{anchor}
1448951_at	Tnfrsf1b	0.225151	
1437001_at	Gsk3b	0.039469	
1451020_at	Gsk3b	0.063825	
1454958_at	Gsk3b	-0.18383	
