# Supplementary material for: Biologically anchored knowledge expansion approach uncovers KLF4 as a novel insulin signaling regulator
Source: PLoS One. 2018 Sep 21;13(9):e0204100. doi: 10.1371/journal.pone.0204100 (PMC6150497; doi:10.1371/journal.pone.0204100)
Supplement: S4 Table — Fold Changes (FC) in expression of neighbor genes between DW16 and DC16 are given in logarithmic scale (base 2). (PDF) [file pone.0204100.s008.pdf]

S4 Table. Twenty Novel Neighbor Genes Around Anchor Gene IRS2 (L_{IRS2})

Probe Set ID	Gene Symbol	Gene Title	Log_2FC
1417395_at	KLF4	Kruppel-like factor 4 (gut)	-2.29942
1439859_at	C12orf5	chromosome 12 open reading frame 5	0.776905
1448668_a_at	IRAK1	interleukin-1 receptor-associated kinase 1	-2.08696
1452045_at	ZNF281	zinc finger protein 281	-1.05731
1437633_at	ANKRD11	ankyrin repeat domain 11	-1.62611
1419048_at	PCNX	pecanex homolog (Drosophila)	-0.9939
1460005_at	BOD1L	biorientation of chromosomes in cell division 1-like	-0.92322
1425975_a_at	MAPK8IP3	mitogen-activated protein kinase 8 interacting protein 3	-1.32734
1429415_at	ZMYND8	zinc finger, MYND-type containing 8	-1.00064
1419191_at	HIPK3	homeodomain interacting protein kinase 3	-1.06855
1437745_at	CHD7	chromodomain helicase DNA binding protein 7	-1.44179
1436363_a_at	NFIX	nuclear factor I/X (CCAAT-binding transcription factor)	-1.47594

1438022_at	RAB11FIP3	RAB11 family interacting protein 3 (class II)	-0.72432
1428472_at	SPSB1	splA/ryanodine receptor domain and SOCS box containing 1	-1.0807
1427978_at	KIAA0494	KIAA0494	-1.43792
1433933_s_at	SLCO2B1	solute carrier organic anion transporter family, member 2B1	-0.83896
1436364_x_at	NFIX	nuclear factor I/X (CCAAT-binding transcription factor)	-1.55931
1448298_at	TNK2	tyrosine kinase, non-receptor, 2	-1.52558
1433142_at	4921504P05Rik	RIKEN cDNA 4921504P05 gene	0.399526
1422584_at	SKIV2L	superkiller viralicidic activity 2-like (S. cerevisiae)	-1.51695
