# Supplementary material for: Biologically anchored knowledge expansion approach uncovers KLF4 as a novel insulin signaling regulator
Source: PLoS One. 2018 Sep 21;13(9):e0204100. doi: 10.1371/journal.pone.0204100 (PMC6150497; doi:10.1371/journal.pone.0204100)
Supplement: S5 Table — Nucleotide sequences 10 kb upstream of initiation start site ATG were scanned for KLF4 binding motifs ({G/A}{G/A}GG{C/T}G{C/T}) and the positions of motifs compared between human, mouse and rat promoters. Motifs shown in red were considered conserved based on the following criteria. They were found in the promoter of all three species, and located no more than 100 bases of each other across the different species and no more than ~ 1000 bases from the start site. Motifs that were less conserved across the three species due to single base variations are shown in green. Positions in promoters are given relative to translation start sites. (PDF) [file pone.0204100.s009.pdf]

S5 Table. IRS2 Promoter Analysis

Binding Site	Position in Mouse Promoter	Position in Human Promoter	Position in Rat Promoter
1. GGGGCGC	1366, 1094	1679, 1129, 706, 646	1356, 1327, 1078
2. GGGGCGT	1337, 580		
3. GGGGTGC		1878, 1582	1379
4. GGGGTGT			
5. GAGGCGC			
6. GAGGCGT			
7. GAGGTGC			
8. GAGGTGT			
9. AGGGCGC	1344	1722, 1702, 1268,198	1334, 845
10. AGGGCGT			
11. AGGGTGC	9765	7188	
12. AGGGTGT	2138, 1875		2128
13. AAGGCGC			
14. AAGGCGT	4467		
15. AAGGTGC	5984		9029, 4776
16. AAGGTGT	6300	6218, 3383	5896
