# Supplementary material for: Biologically anchored knowledge expansion approach uncovers KLF4 as a novel insulin signaling regulator
Source: PLoS One. 2018 Sep 21;13(9):e0204100. doi: 10.1371/journal.pone.0204100 (PMC6150497; doi:10.1371/journal.pone.0204100)
Supplement: S6 Table — Fold Changes (FC) in expression of neighbor genes between DW16 and DC16 are given in logarithmic scale (base 2). (PDF) [file pone.0204100.s010.pdf]

S6 Table. Twenty Novel Neighbor Genes Around Anchor Gene KLF4 (L_{KLF4})

Probe Set ID	Gene Symbol	Gene Title	Log_2(FC)
1452105_a_at	Tsc2	tuberous sclerosis 2	-2.126079
1421861_at	Clstn1	calsyntenin 1	-1.361094
1448189_a_at	Flii	flightless I homolog (Drosophila)	-1.334569
1422521_at	Dctn1	dynactin 1	-1.903575
1428596_at	Tbc1d9b	TBC1 domain family, member 9B	-0.965517
1438022_at	Rab11fip3	RAB11 family interacting protein 3 (class II)	-0.724315
1454645_at	Mgrn1	mahogunin, ring finger 1	-1.43729
1427287_s_at	Itpr2	inositol 1,4,5-triphosphate receptor 2	-1.181218
1456880_at	---	---	-0.881965
1423622_a_at	Ccnl1	cyclin L1	-1.141852
1443969_at	Irs2	insulin receptor substrate 2	-1.434855
1450406_a_at	St3gal3	ST3 beta-galactoside alpha-2,3-sialyltransferase 3	-1.119139
1429415_at	Zmynd8	zinc finger, MYND-type containing 8	-1.000644
1422453_at	Prpf8	pre-mRNA processing factor 8	-1.831634
1418467_at	Smarcd3	SWI/SNF related, matrix associated, actin dependent regulator of chromatin, subfamily d, member 3	-0.831901

1433937_at	Trp53bp2	transformation related protein 53 binding protein 2	-1.143585
1416576_at	Socs3	suppressor of cytokine signaling 3	-2.425183
1460005_at	Bod1l	biorientation of chromosomes in cell division 1-like	-0.923224
1452045_at	Zfp281	zinc finger protein 281	-1.057306
1451521_x_at	Eif4h	eukaryotic translation initiation factor 4H	-1.03088
