# Supplementary material for: Biologically anchored knowledge expansion approach uncovers KLF4 as a novel insulin signaling regulator
Source: PLoS One. 2018 Sep 21;13(9):e0204100. doi: 10.1371/journal.pone.0204100 (PMC6150497; doi:10.1371/journal.pone.0204100)
Supplement: S7 Table — Promoter regions of human, mouse and rat TSC2 were analyzed as described in legend to Suppl. Table S5. Positions in promoters are given relative to translation start sites. (PDF) [file pone.0204100.s011.pdf]

S7 Table. TSC2 Promoter Analysis

Binding Site	Position in Mouse Promoter	Position in Human Promoter	Position in Rat Promoter
1. GGGGCGC		10056	
2. GGGGCGT			
3. GGGGTGC		8179, 2195, 2124, 605	
4. GGGGTGT	512, 64		4906, 4882, 532
5. GAGGCGC			
6. GAGGCGT			
7. GAGGTGC	5301, 2970	8211, 7217	5890, 4607, 4307, 1580
8. GAGGTGT	2097	72	
9. AGGGCGC			
10. AGGGCGT			6697
11. AGGGTGC	1790	8638, 2577	4003
12. AGGGTGT		7443	9761, 3358
13. AAGGCGC	469	1070	489
14. AAGGCGT			3550
15. AAGGTGC	4438	2596, 2019	875
16. AAGGTGT		7453	
