# Supplementary material for: Biologically anchored knowledge expansion approach uncovers KLF4 as a novel insulin signaling regulator
Source: PLoS One. 2018 Sep 21;13(9):e0204100. doi: 10.1371/journal.pone.0204100 (PMC6150497; doi:10.1371/journal.pone.0204100)
Supplement: S8 Table — List of differentially expressed adipogenesis network genes (Lpath adipog, 52 genes represented by 73 Affymetrix probes) between DW16 and DC16 (p<0.01) are shown with fold changes in expression (FC in logarithmic scale to base 2). Among these, 20 genes (represented by 20 Affymetrix probes) were randomly selected as anchor genes (Lanchor adipog). The remaining genes formed the list of hidden genes (Lhidden) to be discovered by BAKE through network expansion around anchor genes. (PDF) [file pone.0204100.s012.pdf]

S8 Table. List of Differentially Expressed Adipogenesis Network and Anchor Genes.

$L_{path\ adipog}$ Genes				
Probe Set ID	Gene Symbol	Gene Title	Log_2FC	$L_{anchor\ adipog}$ Genes
1422651_at	Adipoq	adiponectin, C1Q and collagen domain containing	-0.421347	
1435077_at	ASXL1	additional sex combs like 1	-0.542604	
1422912_at	BMP4	bone morphogenetic protein 4	-1.195104	
1418982_at	CEBPa	CCAAT/enhancer binding protein (C/EBP), alpha	-1.055289	
1427844_a_at	CEBPb	CCAAT/enhancer binding protein (C/EBP), beta	-1.416855	L_{anchor}
1418901_at	CEBPb	CCAAT/enhancer binding protein (C/EBP), beta	-0.727956	
1423233_at	CEBPd	CCAAT/enhancer binding protein (C/EBP), delta	-1.336919	
1456605_at	CEBPd	CCAAT/enhancer binding protein (C/EBP), delta	-0.516927	L_{anchor}
1437745_at	CHD7	chromodomain helicase DNA binding protein 7	-1.441792	L_{anchor}
1438992_x_at	CREB2	activating transcription factor 4	0.514875	
1448135_at	CREB2	activating transcription factor 4	-0.441271	L_{anchor}
1424471_at	EPAC	Rap guanine nucleotide exchange factor (GEF) 3	-0.974207	L_{anchor}
1427060_at	ERK1	mitogen-activated protein kinase 3	-0.883972	L_{anchor}
1426585_s_at	ERK2	mitogen-activated protein kinase 1	-1.202173	
1416544_at	EZH2	enhancer of zeste homolog 2 (Drosophila)	-0.497959	
1448886_at	GATA3	GATA binding protein 3	-0.418089	L_{anchor}
1421866_at	GR	nuclear receptor subfamily 3, group C, member 1	-0.788955	
1421867_at	GR	nuclear receptor subfamily 3, group C, member 1	-1.375725	
1433444_at	HMGCS1	3-hydroxy-3-methylglutaryl-Coenzyme A synthase 1	-0.797982	
1433445_x_at	HMGCS1	3-hydroxy-3-methylglutaryl-Coenzyme A synthase 1	-0.609106	L_{anchor}
1424458_at	JMJDC2C	lysine (K)-specific demethylase 4C	-0.868275	
1417395_at	KLF4	Kruppel-like factor 4 (gut)	-2.299423	
1451022_at	LRP6	low density lipoprotein receptor-related protein 6	0.746302	L_{anchor}
1427150_at	MLL3	lysine (K)-specific methyltransferase 2C	-0.518829	
1416572_at	MMP14	matrix metallopeptidase 14 (membrane-inserted)	-0.48744	

1448383_at	MMP14	matrix metallopeptidase 14 (membrane-inserted)	-1.078622	
1423202_a_at	NCOR1	nuclear receptor co-repressor 1	-0.722665	L_{anchor}
1448893_at	NCOR2	nuclear receptor co-repressor 2	-1.35313	
1456467_s_at	NLK	nemo like kinase	0.742247	
1425837_a_at	Nocturnin	CCR4 carbon catabolite repression 4-like (S. cerevisiae)	-0.806877	
1426464_at	NR1D1	nuclear receptor subfamily 1, group D, member 1	-1.475204	L_{anchor}
1425615_a_at	PCK2	phosphoenolpyruvate carboxykinase 2 (mitochondrial)	-0.925597	
1425580_a_at	PIK3C3	phosphoinositide-3-kinase, class 3	-0.779372	
1429434_at	PIK3Ca	phosphatidylinositol 3-kinase, catalytic, alpha polypeptide	0.813153	
1429435_x_at	PIK3Ca	phosphatidylinositol 3-kinase, catalytic, alpha polypeptide	0.677044	
1460326_at	PIK3Ca	phosphatidylinositol 3-kinase, catalytic, alpha polypeptide	-0.837878	
1453069_at	PIK3Cb	phosphatidylinositol 3-kinase, catalytic, beta polypeptide	-1.025567	L_{anchor}
1443798_at	PIK3Cd	phosphatidylinositol 3-kinase catalytic delta polypeptide	2.096801	L_{anchor}
1420611_at	PKA	protein kinase, cAMP dependent, catalytic, beta	-0.948384	
1450519_a_at	PKA	protein kinase, cAMP dependent, catalytic, alpha	-1.385787	
1423302_a_at	PTIP	PAX interacting (with transcription-activation domain) protein 1	-0.622208	
1423303_at	PTIP	PAX interacting (with transcription-activation domain) protein 1	-1.382188	
1454943_a_at	PTIP	PAX interacting (with transcription-activation domain) protein 1	-0.423649	
1423734_at	RAC1	RAS-related C3 botulinum substrate 1	-0.736388	
1425762_a_at	RXRa	retinoid X receptor alpha	-0.5068	L_{anchor}
1454773_at	RXRa	retinoid X receptor alpha	-1.106345	
1416990_at	RXRb	retinoid X receptor beta	-0.675337	
1426406_at	SETD8	SET domain containing (lysine methyltransferase) 8	-0.902194	
1416670_at	SETDB1	SET domain, bifurcated 1	0.615873	
1451833_a_at	SETDB1	SET domain, bifurcated 1	-0.446396	
1423507_a_at	SIRT2	sirtuin 2	-0.733882	L_{anchor}
1415958_at	SLC2A4	solute carrier family 2 (facilitated glucose transporter), member 4	-1.523955	

1415959_at	SLC2A4	solute carrier family 2 (facilitated glucose transporter), member 4	-2.300591	
1459843_s_at	SMAD1	SMAD family member 1	0.881402	
1448994_at	SP1	trans-acting transcription factor 1	0.472075	
1426690_a_at	SREBF1	sterol regulatory element binding transcription factor 1	-1.385623	L_{anchor}
1450008_a_at	ßcatenin	catenin (cadherin associated protein), beta 1	-2.198538	
1420811_a_at	ßcatenin	catenin (cadherin associated protein), beta 1	-0.817598	
1426587_a_at	STAT3	signal transducer and activator of transcription 3	-1.599634	L_{anchor}
1459961_a_at	STAT3	signal transducer and activator of transcription 3	-1.049479	
1460700_at	STAT3	signal transducer and activator of transcription 3	-1.144656	
1421469_a_at	STAT5A	signal transducer and activator of transcription 5A	-1.101546	
1417440_at	SWI1	AT rich interactive domain 1A (SWI-like)	-1.505176	
1455979_at	SWI1	AT rich interactive domain 1B (SWI-like)	0.518932	
1426804_at	SWI2	SWI/SNF related, matrix associated, actin dependent regulator of chromatin, subfamily a, member 4	-1.263507	
1426805_at	SWI2	SWI/SNF related, matrix associated, actin dependent regulator of chromatin, subfamily a, member 4	-1.912802	
1450461_at	TCF7	transcription factor 7, T cell specific	0.461626	
1433471_at	TCF7	transcription factor 7, T cell specific	-0.295828	L_{anchor}
1423250_a_at	TGFB2	transforming growth factor, beta 2	-0.318399	L_{anchor}
1450922_a_at	TGFB2	transforming growth factor, beta 2	-0.711545	
1419089_at	TIMP3	tissue inhibitor of metalloproteinase 3	-1.475497	
1458512_at	TLE3	transducin-like enhancer of split 3, homolog of Drosophila E(spl)	-0.911589	L_{anchor}
1451006_at	XDH	xanthine dehydrogenase	-1.057305	
