# Supplementary material for: Biologically anchored knowledge expansion approach uncovers KLF4 as a novel insulin signaling regulator
Source: PLoS One. 2018 Sep 21;13(9):e0204100. doi: 10.1371/journal.pone.0204100 (PMC6150497; doi:10.1371/journal.pone.0204100)
Supplement: S9 Table — Genes are listed in alphabetical order. Hidden adipogenesis network genes discovered among novel genes are in bold. (PDF) [file pone.0204100.s013.pdf]

S9 Table. Novel Genes Discovered by BAKE during Adipogenesis Network Expansion.

**Novel Genes**
4933407C03Rik
A930012O16Rik
APLP2
ARF4
ATP11A
ATRX
CCL11
CDKN1C
**CEBPb***
CHUK
COPS7A
CSNK1D
CXCL12
D5ERTD579E
DDX60
GCLM
GNA11
GPC6
MRPL17
NSMCE4A
NUP98
**PCK2**
PPP1R12C

PTPRB
RUSC2
SAP30I
**SETDB1**
SGP1
SNX6
SPSB1
SS18
**TGFB2***
ThAP4
**TIMP3**
TOR1AP1
**XDH**
ZXSCAN17

* Genes TGFB2 and CEBPb had multiple probes that appeared both among the list of anchor genes ($L_{anchor\ adipog}$) and hidden genes (L_{hidden}).
