# Supplementary material for: Biologically anchored knowledge expansion approach uncovers KLF4 as a novel insulin signaling regulator
Source: PLoS One. 2018 Sep 21;13(9):e0204100. doi: 10.1371/journal.pone.0204100 (PMC6150497; doi:10.1371/journal.pone.0204100)
Supplement: S10 Table — Anchor gene-anchor gene and anchor gene-hidden gene interactions inferred by BAKE are shown along with their classification as True Positives (TPs) and False Positives (FPs). Literature evidence for TP gene interactions were obtained from the adipogenesis network shown in S3 Fig or the network analysis tool Ingenuity Pathway Analysis (IPA®). In IPA gene interactions are derived and curated from a variety of databases such as Ingenuity Expert Information, microRNA-mRNA interaction database (miRecords), protein-protein interaction databases (including BIND, Cognia, MIPS), BioGRID, Gene Ontology (GO), Online Mendelian Inheritance in Man (OMIM) and Mouse Genome Database (MGD). (PDF) [file pone.0204100.s014.pdf]

S10 Table. Gene Interactions inferred by BAKE during Adipogenesis Network Expansion.

Gene Interaction #	Gene 1	Gene 2	TP/FP	Evidence
1	CEBPb	CEBPb	TP	Different probes derived from the same gene
2	CEBPd	PCK2	TP	Regulation of PCK2 by CEBPd is a long distance interaction (Fig. S3)
3	STAT3	XDH	TP	Regulation of XDH by STAT3 is shown in Fig. S3
4	CEBPb	TGFB2	TP	Regulation of XDH by CEBPb is shown in Fig. S3. In homozygous Xor [Xdh] gene knockout mice expression of mouse TGF-ß proteins is increased in cytosol of kidney tubular cells (IPA®).
5	GATA3	TIMP3	TP	In synovial fibroblasts from human exhibiting rheumatoid arthritis, TIMP3 protein decreases activation of NFkB complex(es). Activation of NFkB complex(es) is involved in GATA3 mRNA expression (IPA®).
6	HMGCS1	PIK3Cb	TP	Long distance regulation (Fig. S3)
7	CEBPb	STAT3	TP	Regulation of CEBPb by STAT3 is shown in Fig. S3
8	CREB2	CEBPb	TP	Regulation of CEBPb by CREB2 is shown in Fig. S3
9	CEBPb	SREBF1	TP	Regulation of CEBPb by SREBF1 is shown in Fig. S3
10	RXRa	SREBF1	TP	Analysis of the mouse SREBF1 gene promoter revealed an RXR/LXR DNA-binding site that is essential for SREBF1 transcriptional regulation (IPA®).
11	GATA3	TGFB2	TP	TGFB protein(s) decrease(s) expression of GATA3 protein in vitro (IPA®).
12	GATA3	SREBF1	TP	Regulation of CEBPb by SREBF1 is shown in Fig. S3. A regulatory interaction between CEBPb and TGFB2 is explained above for 4. A regulatory interaction between TGFB2 and GATA3 is explained above for 11. It is a long linear distance interaction.
13	CEBPb	NR1D1	TP	NR1D1 regulates the expression of CEBPa, which can regulate PPARg through a feedback loop, which can in turn regulate CEBPb also through a feedback loop. All these interactions are shown in Fig. S3.
14	CREB2	TGFB2	TP	CREB2 regulates CEBPb as shown in Fig. S3. Regulation of TGFB2 by CEBPb is explained above for 4. It is a linear long distance interaction.
15	STAT3	TGFB2	TP	STAT3 regulates CEBPb as shown in Fig. S3. Regulation of TGFB2 by CEBPb is explained above for 4. It is a linear long distance interaction.

16	CREB2	STAT3	TP	Activating transcription factor 4 (ATF4/CREB2) mediates hyperglycemia-induced endothelial inflammation and retinal vascular leakage through activation of STAT3 in a mouse model of type 1 diabetes (IPA®).
17	GATA3	TLE3	TP	TLE3 regulates expression of CEBPa, which can then regulate CEBPa (due to feedback loop), which can then feedback to the expression of CEBPb. Regulation of TGFB2 by CEBPb is explained above for 4. TGFB2 regulates GATA3 as explained above for 12. Therefore, a regulatory relationship between GATA3 and TLE3 can be inferred (IPA®).
18	CHD7	GATA3	TP	CHD7 regulates the expression of PPARg (Fig. S3). PPARg can in turn regulate CEBPb through a feedback loop. Regulation of TGFB2 by CEBPb is explained above for 4. TGFB2 regulates GATA3 as explained above for 11. Therefore, a regulatory relationship between GATA3 and CHD7 can be inferred (IPA®).
19	GATA3	RXRa	TP	RXRa regulates the expression of PPARg (Fig. S3), which then can be expected to interact with GATA3 using the same reasoning as used for 18. Therefore, a regulatory relationship between GATA3 and RXRa can be inferred.
20	GATA3	ERK1	TP	ERK1 regulates the expression of CEBPa (Fig. S3), which shares a regulatory feedback loop with PPARg. The relationship between PPARg and GATA3 can be inferred as for 19. Therefore, a regulatory relationship between GATA3 and ERK1 can be inferred.
21	NCOR1	NR1D1	TP	In C2C12 cells, the receptor interacting domain of RIP13 [NCOR1] decreases repression of a promoter fragment derived from the human Rev-ErbAalpha [NR1D1] gene that is increased by RVR [NR1D2] protein (IPA®).
22	NR1D1	TGFB2	TP	NR1D1 regulates CEBPa expression, which can regulate PPARg through a feedback loop, which can in turn regulate CEBPb also through a feedback loop (Fig. S3). The regulatory relationship between CEBPb and TGFB2 can be reasoned as for 4.
23	PIK3Cd	SETDB1	FP	
24	ERK1	RXRa	FP	
25	CHD7	TLE3	FP	
26	CREB2	SREBF1	FP	
27	SREBF1	STAT3	FP	
28	TCF7	TGFB2	FP	
29	RXRa	SIRT2	FP	
30	PIK3Cd	RXRa	FP	

31	PIK3Cd	SIRT2	FP	
32	EPAC	TLE3	FP	
33	CHD7	EPAC	FP	
34	GATA3	EPAC	FP	
35	GATA3	LRP6	FP	
36	LRP6	SREBF1	FP	
37	PIK3Cd	TLE3	FP	
38	CHD7	PIK3Cd	FP	
39	GATA3	SIRT2	FP	
40	NR1D1	PIK3Cb	FP	
41	NCOR1	PIK3Cb	FP	
42	CREB2	NR1D1	FP	
43	EPAC	RXRa	FP	
44	EPAC	SIRT2	FP	
45	LRP6	NCOR1	FP	
