# Supplementary material for: Correction: Biologically anchored knowledge expansion approach uncovers KLF4 as a novel insulin signaling regulator
Source: PLoS One. 2018 Nov 7;13(11):e0207325. doi: 10.1371/journal.pone.0207325 (PMC6221346; doi:10.1371/journal.pone.0207325)
Supplement: S2 Table — L0 represents genes that were differentially expressed between DW16 and DC16 adipocytes. L1 represents genes in L0 for which expression profiles significantly correlated with expression of insulin signaling pathway genes (Lpath) in adipocytes using data for all four conditions DC8, DW8, DC16 and DW16 (marked L1 in table). (PDF) [file pone.0207325.s001.pdf]

S2 Table. L_0 and L_1 Genes

L_0 Genes			
**Probe Set ID**	**Gene Symbol**	**Gene Title**	**L_1 Genes**
1452925_a_at	MARCH5	membrane-associated ring finger (C3HC4) 5	L1
1438678_at	1500011K16Rik	RIKEN cDNA 1500011K16 gene	
1428732_at	1700008J07Rik	RIKEN cDNA 1700008J07 gene	L1
1453294_at	1700012B15Rik	RIKEN cDNA 1700012B15 gene	L1
1441871_at	1810044D09Rik	RIKEN cDNA 1810044D09 gene	
1429115_at	2010003O02Rik	RIKEN cDNA 2010003O02 gene	L1
1432607_at	2610012C04Rik	RIKEN cDNA 2610012C04 gene	L1
1453745_at	2700038G22Rik	RIKEN cDNA 2700038G22 gene	L1
1436235_x_at	4732471D19Rik	RIKEN cDNA 4732471D19 gene	
1427326_at	4732471D19Rik	RIKEN cDNA 4732471D19 gene	L1
1431580_at	4833421K07Rik	RIKEN cDNA 4833421K07 gene	
1433142_at	4921504P05Rik	RIKEN cDNA 4921504P05 gene	L1
1429880_at	4921531C22Rik	RIKEN cDNA 4921531C22 gene	
1453390_at	4930428F12Rik	RIKEN cDNA 4930428F12 gene	L1
1433197_at	4930445B03Rik	RIKEN cDNA 4930445B03 gene	L1
1433082_at	4930448K20Rik	glyceraldehyde-3-phosphate dehydrogenase pseudogene	
1431634_at	4930455C13Rik	RIKEN cDNA 4930455C13 gene	L1
1430175_at	4930588G05Rik	RIKEN cDNA 4930588G05 gene	
1432626_at	5730507A11Rik	RIKEN cDNA 5730507A11 gene	L1
1430646_at	5830407P18Rik	RIKEN cDNA 5830407P18 gene	L1
1433685_a_at	6430706D22Rik	RIKEN cDNA 6430706D22 gene	L1
1432680_at	9130009M17Rik	RIKEN cDNA 9130009M17 gene	L1
1438299_at	9230108I15Rik	RIKEN cDNA 9230108I15 gene	
1439874_at	9330102E08Rik	RIKEN cDNA 9330102E08 gene	L1
1454592_at	9430012M22Rik	RIKEN cDNA 9430012M22 gene	
1456758_at	9930017N22Rik	RIKEN cDNA 9930017N22 gene	L1
1457517_at	A730046G19Rik	RIKEN cDNA A730046G19 gene	
1439110_at	A930012O16Rik	RIKEN cDNA A930012O16 gene	L1
1436942_at	A930035D04Rik	RIKEN cDNA A930035D04 gene	
1444070_at	AA914427	EST AA914427	
1452233_at	ABCC1	ATP-binding cassette, sub-family C (CFTR/MRP), member 1	L1
1439835_x_at	ABCD2	ATP-binding cassette, sub-family D (ALD), member 2	
1416014_at	ABCE1	ATP-binding cassette, sub-family E (OABP), member 1	L1

1452236_at	ABCF1	ATP-binding cassette, sub-family F (GCN20), member 1	
1423570_at	ABCG1	ATP-binding cassette, sub-family G (WHITE), member 1	
1427959_at	ABHD10	abhydrolase domain containing 10	L1
1418661_at	ABHD2	abhydrolase domain containing 2	
1417946_at	ABHD3	abhydrolase domain containing 3	L1
1455313_at	ABLIM2	actin binding LIM protein family, member 2	L1
1427595_at	ACACA	acetyl-CoA carboxylase alpha	
1453206_at	ACAD9	acyl-CoA dehydrogenase family, member 9	
1423644_at	ACO1	aconitase 1, soluble	L1
1431609_a_at	ACP5	acid phosphatase 5, tartrate resistant	
1448445_at	ACP6	acid phosphatase 6, lysophosphatidic	L1
1460316_at	ACSL1	acyl-CoA synthetase long-chain family member 1	L1
1427735_a_at	ACTA1	actin, alpha 1, skeletal muscle	
1416569_at	ACTL6A	actin-like 6A	L1
1423449_a_at	ACTN4	actinin, alpha 4	L1
1417157_at	ACTR10	actin-related protein 10 homolog (S. cerevisiae)	L1
1422561_at	ADAMTS5	ADAM metallopeptidase with thrombospondin type 1 motif, 5	
1433556_at	ADAP1	ArfGAP with dual PH domains 1	L1
1439613_at	ADCY10	adenylate cyclase 10 (soluble)	
1419811_at	ADCY9	adenylate cyclase 9	L1
1434454_at	ADCY9	adenylate cyclase 9	L1
1420953_at	ADD1	adducin 1 (alpha)	L1
1441094_at	Adh6b	alcohol dehydrogenase 6B (class V)	
1434329_s_at	ADIPOR2	adiponectin receptor 2	L1
1416319_at	ADK	adenosine kinase	L1
1450214_at	ADORA2B	adenosine A2b receptor	L1
1451992_at	ADRBK1	adrenergic, beta, receptor kinase 1	L1
1418372_at	ADSL	adenylosuccinate lyase	L1
1454727_at	AFAP1L1	actin filament associated protein 1-like 1	L1

1426923_at	AGFG1	ArfGAP with FG repeats 1	L1
1452237_at	AGFG1	ArfGAP with FG repeats 1	L1
1435591_at	AI426330	expressed sequence AI426330	L1
1442197_at	AI480624	expressed sequence AI480624	
1456399_at	AI842136	expressed sequence AI842136	L1
1443923_at	AKAP13	A kinase (PRKA) anchor protein 13	L1
1447912_x_at	AKAP9	A kinase (PRKA) anchor protein (yotiao) 9	L1
1448894_at	AKR1B10	aldo-keto reductase family 1, member B10 (aldose reductase)	L1
1428158_at	AKT1S1	AKT1 substrate 1 (proline-rich)	L1
1426300_at	ALCAM	activated leukocyte cell adhesion molecule	
1418844_at	ALG9	asparagine-linked glycosylation 9, alpha-1,2-mannosyltransferase homolog (S. cerevisiae)	
1434660_at	ALKBH1	alkB, alkylation repair homolog 1 (E. coli)	L1
1428904_at	AMMECR1L	AMME chromosomal region gene 1-like	L1
1422573_at	AMPD3	adenosine monophosphate deaminase 3	L1
1415707_at	ANAPC2	anaphase promoting complex subunit 2	
1448008_at	ANKHD1-EIF4EBP3	ANKHD1-EIF4EBP3 readthrough	L1
1435778_at	ANKRD11	ankyrin repeat domain 11	L1
1436967_at	ANKRD11	ankyrin repeat domain 11	L1
1437633_at	ANKRD11	ankyrin repeat domain 11	L1
1456110_at	ANKRD11	ankyrin repeat domain 11	L1
1443867_at	ANKRD12	ankyrin repeat domain 12	L1
1428239_at	ANKRD16	ankyrin repeat domain 16	L1
1444016_at	ANXA1	annexin A1	L1
1416307_at	AP1M1	adaptor-related protein complex 1, mu 1 subunit	L1
1426719_at	APBB2	amyloid beta (A4) precursor protein-binding, family B, member 2	L1
1424980_s_at	APH1A	anterior pharynx defective 1 homolog A (C. elegans)	L1

1421887_a_at	APLP2	amyloid beta (A4) precursor-like protein 2	L1
1423739_x_at	APLP2	amyloid beta (A4) precursor-like protein 2	L1
1416371_at	APOD	apolipoprotein D	
1452800_a_at	APOO	apolipoprotein O	L1
1429248_at	APOPT1	apoptogenic 1, mitochondrial	L1
1416203_at	AQP1	aquaporin 1 (Colton blood group)	L1
1418687_at	ARC	activity-regulated cytoskeleton-associated protein	L1
1421134_at	AREG/AREGB	amphiregulin	L1
1423052_at	ARF4	ADP-ribosylation factor 4	L1
1423053_at	ARF4	ADP-ribosylation factor 4	L1
1426534_a_at	ARFGAP3	ADP-ribosylation factor GTPase activating protein 3	L1
1424240_at	ARFIP2	ADP-ribosylation factor interacting protein 2	L1
1451309_at	ARHGAP1	Rho GTPase activating protein 1	L1
1431133_at	ARHGAP18	Rho GTPase activating protein 18	L1
1423194_at	ARHGAP5	Rho GTPase activating protein 5	L1
1419457_at	ARHGEF28	Rho guanine nucleotide exchange factor (GEF) 28	L1
1436725_at	ARHGEF39	Rho guanine nucleotide exchange factor (GEF) 39	
1436768_x_at	ARHGEF40	Rho guanine nucleotide exchange factor (GEF) 40	
1455810_a_at	ARHGEF40	Rho guanine nucleotide exchange factor (GEF) 40	
1431024_a_at	ARID4B	AT rich interactive domain 4B (RBP1-like)	
1451847_s_at	ARID4B	AT rich interactive domain 4B (RBP1-like)	L1
1420973_at	ARID5B	AT rich interactive domain 5B (MRF1-like)	L1
1417331_a_at	ARL6	ADP-ribosylation factor-like 6	L1
1425914_a_at	ARMCX1	armadillo repeat containing, X-linked 1	
1416226_at	ARPC1B	actin related protein 2/3 complex, subunit 1B, 41kDa	L1
1425572_a_at	ASAP1	ArfGAP with SH3 domain, ankyrin repeat and PH domain 1	

1425573_a_at	ASAP1	ArfGAP with SH3 domain, ankyrin repeat and PH domain 1	
1417361_at	ASB3/GPR75-ASB3	ankyrin repeat and SOCS box containing 3	L1
1437086_at	ASCL1	achaete-scute complex homolog 1 (Drosophila)	
1425274_at	ASPH	aspartate beta-hydroxylase	
1450058_at	ASPH	aspartate beta-hydroxylase	
1435077_at	ASXL1	additional sex combs like 1 (Drosophila)	L1
1446838_at	ATAD1	ATPase family, AAA domain containing 1	
1429396_at	ATG16L2	autophagy related 16-like 2 (S. cerevisiae)	L1
1428260_at	ATL1	atlastin GTPase 1	L1
1447851_x_at	ATP10A	ATPase, class V, type 10A	L1
1456388_at	ATP11A	ATPase, class VI, type 11A	
1427481_a_at	ATP1A3	ATPase, Na+/K+ transporting, alpha 3 polypeptide	
1416551_at	ATP2A2	ATPase, Ca++ transporting, cardiac muscle, slow twitch 2	
1427251_at	ATP2A2	ATPase, Ca++ transporting, cardiac muscle, slow twitch 2	L1
1452363_a_at	ATP2A2	ATPase, Ca++ transporting, cardiac muscle, slow twitch 2	L1
1416391_at	ATP5J2-PTCD1/PTCD1	pentatricopeptide repeat domain 1	
1420947_at	ATRX	alpha thalassemia/mental retardation syndrome X-linked	
1420946_at	ATRX	alpha thalassemia/mental retardation syndrome X-linked	L1
1422576_at	ATXN10	ataxin 10	L1
1419866_s_at	ATXN2	ataxin 2	L1
1460653_at	ATXN2	ataxin 2	L1
1439497_at	ATXN7L1	ataxin 7-like 1	L1
1455492_at	B330016D10Rik	RIKEN cDNA B330016D10 gene	
1423084_at	B3GALT2	UDP-Gal:betaGlcNAc beta 1,3-galactosyltransferase, polypeptide 2	
1425934_a_at	B4GALT4	UDP-Gal:betaGlcNAc beta 1,4-galactosyltransferase, polypeptide 4	L1

1421824_at	BACE1	beta-site APP-cleaving enzyme 1	L1
1425656_a_at	BAIAP2	BAI1-associated protein 2	L1
1451539_at	BAIAP2L1	BAI1-associated protein 2-like 1	
1423753_at	BAMBI	BMP and activin membrane-bound inhibitor homolog (Xenopus laevis)	L1
1426354_at	BAP1	BRCA1 associated protein-1 (ubiquitin carboxy-terminal hydrolase)	L1
1433599_at	BAZ1A	bromodomain adjacent to zinc finger domain, 1A	L1
1440805_at	BAZ1B	bromodomain adjacent to zinc finger domain, 1B	
1454684_at	BBS7	Bardet-Biedl syndrome 7	L1
1439388_s_at	BCAR1	breast cancer anti-estrogen resistance 1	L1
1450622_at	BCAR1	breast cancer anti-estrogen resistance 1	L1
1415936_at	BCAR3	breast cancer anti-estrogen resistance 3	
1418971_x_at	BCL10	B-cell CLL/lymphoma 10	
1418970_a_at	BCL10	B-cell CLL/lymphoma 10	L1
1418972_at	BCL10	B-cell CLL/lymphoma 10	L1
1424406_at	BCL2L13	BCL2-like 13 (apoptosis facilitator)	L1
1436023_at	BCLAF1	BCL2-associated transcription factor 1	L1
1454172_at	BCORL1	BCL6 corepressor-like 1	
1430549_at	BET1L	blocked early in transport 1 homolog (S. cerevisiae)-like	
1427488_a_at	BIRC6	baculoviral IAP repeat containing 6	
1420631_a_at	BLCAP	bladder cancer associated protein	L1
1426238_at	BMP1	bone morphogenetic protein 1	L1
1421103_at	BMP2K	BMP2 inducible kinase	L1
1422912_at	BMP4	bone morphogenetic protein 4	L1
1419616_at	BMPR2	bone morphogenetic protein receptor, type II (serine/threonine kinase)	
1428669_at	Bmyc	brain expressed myelocytomatosis oncogene	

1426869_at	BOC	Boc homolog (mouse)	L1
1460005_at	BOD1L1	biorientation of chromosomes in cell division 1-like 1	L1
1417040_a_at	BOK	BCL2-related ovarian killer	L1
1418378_at	BPIFB2	BPI fold containing family B, member 2	
1427311_at	BPTF	bromodomain PHD finger transcription factor	L1
1438771_at	BRD1	bromodomain containing 1	L1
1427192_a_at	BRD8	bromodomain containing 8	L1
1451137_a_at	BRD8	bromodomain containing 8	L1
1426312_at	BRE	brain and reproductive organ-expressed (TNFRSF1A modulator)	L1
1435153_at	BTBD6	BTB (POZ) domain containing 6	L1
1432910_at	BTBD7	BTB (POZ) domain containing 7	
1431230_a_at	BTBD9	BTB (POZ) domain containing 9	L1
1449007_at	BTG3	BTG family, member 3	L1
1428632_at	C11orf10	chromosome 11 open reading frame 10	L1
1439859_at	C12orf5	chromosome 12 open reading frame 5	L1
1430095_at	C12orf56	chromosome 12 open reading frame 56	
1451303_at	C14orf102	chromosome 14 open reading frame 102	L1
1416917_at	C14orf119	chromosome 14 open reading frame 119	L1
1428450_at	C15orf38	chromosome 15 open reading frame 38	L1
1426347_at	C15orf44	chromosome 15 open reading frame 44	L1
1426828_at	C16orf7	chromosome 16 open reading frame 7	L1
1452896_at	C16orf80	chromosome 16 open reading frame 80	L1
1420548_a_at	C16orf88	chromosome 16 open reading frame 88	L1
1428809_at	C17orf58	chromosome 17 open reading frame 58	L1
1416886_at	C1D	C1D nuclear receptor corepressor	L1

1419174_at	C1orf52	chromosome 1 open reading frame 52	L1
1416051_at	C2	complement component 2	L1
1457664_x_at	C2	complement component 2	L1
1438038_at	C20orf194	chromosome 20 open reading frame 194	L1
1424724_a_at	C21orf91	chromosome 21 open reading frame 91	L1
1428085_at	C2orf43	chromosome 2 open reading frame 43	L1
1437439_at	C5orf42	chromosome 5 open reading frame 42	L1
1423306_at	C5orf62	chromosome 5 open reading frame 62	L1
1428217_at	C6orf120	chromosome 6 open reading frame 120	
1453818_a_at	C6orf70	chromosome 6 open reading frame 70	L1
1458879_at	C76798	expressed sequence C76798	
1429451_at	C8orf37	chromosome 8 open reading frame 37	L1
1423879_at	C9orf40	chromosome 9 open reading frame 40	
1452307_at	CABLES2	Cdk5 and Abl enzyme substrate 2	L1
1436031_at	CACHD1	cache domain containing 1	
1452829_at	CAD	carbamoyl-phosphate synthetase 2, aspartate transcarbamylase, and dihydroorotase	L1
1427763_a_at	CAMK2D	calcium/calmodulin-dependent protein kinase II delta	L1
1455751_at	CAND1	cullin-associated and neddylation-dissociated 1	L1
1427944_at	CAPRIN2	caprin family member 2	
1452038_at	CAPZA1	capping protein (actin filament) muscle Z-line, alpha 1	L1
1455886_at	CBL	Cbl proto-oncogene, E3 ubiquitin protein ligase	L1
1451531_at	CBWD1	COBW domain containing 1	L1
1421933_at	CBX5	chromobox homolog 5	
1451442_at	CCDC104	coiled-coil domain containing 104	L1

1432094_a_at	CCDC132	coiled-coil domain containing 132	L1
1428261_at	CCDC134	coiled-coil domain containing 134	
1452721_a_at	CCDC53	coiled-coil domain containing 53	L1
1424187_at	CCDC80	coiled-coil domain containing 80	
1427138_at	CCDC88C	coiled-coil domain containing 88C	L1
1428505_at	CCDC90B	coiled-coil domain containing 90B	L1
1417789_at	CCL11	chemokine (C-C motif) ligand 11	L1
1420380_at	CCL13	chemokine (C-C motif) ligand 13	
1423622_a_at	CCNL1	cyclin L1	L1
1432195_s_at	CCNL2	cyclin L2	L1
1428413_at	CCNY	cyclin Y	L1
1433554_at	CCZ1/CCZ1B	CCZ1 vacuolar protein trafficking and biogenesis associated homolog B (S. cerevisiae)	L1
1422105_at	CD3E	CD3e molecule, epsilon (CD3-TCR complex)	L1
1419554_at	CD47	CD47 molecule	L1
1449507_a_at	CD47	CD47 molecule	L1
1456046_at	CD93	CD93 molecule	
1456746_a_at	Cd99l2	CD99 antigen-like 2	L1
1456747_x_at	Cd99l2	CD99 antigen-like 2	L1
1425556_at	CDK12	cyclin-dependent kinase 12	
1455567_at	CDK12	cyclin-dependent kinase 12	L1
1415956_a_at	CDK16	cyclin-dependent kinase 16	L1
1426885_a_at	CDK2AP1	cyclin-dependent kinase 2 associated protein 1	L1
1450674_at	CDK5	cyclin-dependent kinase 5	L1
1457625_s_at	CDKL2	cyclin-dependent kinase-like 2 (CDC2-related kinase)	
1417649_at	CDKN1C	cyclin-dependent kinase inhibitor 1C (p57, Kip2)	
1418982_at	CEBPA	CCAAT/enhancer binding protein (C/EBP), alpha	L1
1427413_a_at	CELF1	CUGBP, Elav-like family member 1	
1429326_at	CENPL	centromere protein L	L1
1452470_at	CEP350	centrosomal protein 350kDa	
1437209_at	CEP76	centrosomal protein 76kDa	L1

1429670_a_at	CEP97	centrosomal protein 97kDa	
1448052_at	CGNL1	cingulin-like 1	
1438476_a_at	CHD4	chromodomain helicase DNA binding protein 4	
1437745_at	CHD7	chromodomain helicase DNA binding protein 7	L1
1455435_s_at	CHDH	choline dehydrogenase	
1451896_a_at	CHERP	calcium homeostasis endoplasmic reticulum protein	L1
1454023_a_at	CHPF	chondroitin polymerizing factor	L1
1452170_at	CHPF2	chondroitin polymerizing factor 2	L1
1426146_a_at	CHPT1	choline phosphotransferase 1	L1
1426866_at	CHST14	carbohydrate (N-acetylgalactosamine 4-0) sulfotransferase 14	
1417091_at	CHUK	conserved helix-loop-helix ubiquitous kinase	L1
1430992_s_at	CISD2	CDGSH iron sulfur domain 2	L1
1448724_at	CISH	cytokine inducible SH2-containing protein	L1
1437271_at	CLCF1	cardiotrophin-like cytokine factor 1	L1
1452702_at	CLCN7	chloride channel, voltage-sensitive 7	L1
1438467_at	CLEC10A	C-type lectin domain family 10, member A	L1
1456318_at	CLEC1A	C-type lectin domain family 1, member A	L1
1448250_at	CLMP	CXADR-like membrane protein	L1
1421861_at	CLSTN1	calsyntenin 1	L1
1441165_s_at	CLSTN2	calsyntenin 2	
1452597_at	CMC2	COX assembly mitochondrial protein 2 homolog (S. cerevisiae)	L1
1427980_at	CMIP	c-Maf inducing protein	L1
1451114_at	CMTM6	CKLF-like MARVEL transmembrane domain containing 6	L1
1436436_at	CNIH4	cornichon homolog 4 (Drosophila)	
1450981_at	CNN2	calponin 2	L1

1425480_at	CNOT6L	CCR4-NOT transcription complex, subunit 6-like	L1
1430519_a_at	CNOT7	CCR4-NOT transcription complex, subunit 7	L1
1421292_a_at	COBRA1	cofactor of BRCA1	L1
1433774_x_at	COG1	component of oligomeric golgi complex 1	L1
1449053_s_at	COG1	component of oligomeric golgi complex 1	L1
1416692_at	COIL	coilin	L1
1448755_at	COL15A1	collagen, type XV, alpha 1	L1
1424051_at	COL4A2	collagen, type IV, alpha 2	
1419703_at	COL5A3	collagen, type V, alpha 3	L1
1454642_a_at	COMMD3	COMM domain containing 3	L1
1449694_s_at	COMMD5	COMM domain containing 5	
1423301_at	COPB1	coatomer protein complex, subunit beta 1	L1
1416163_at	COPS4	COP9 constitutive photomorphogenic homolog subunit 4 (Arabidopsis)	L1
1423245_at	COPS7A	COP9 constitutive photomorphogenic homolog subunit 7A (Arabidopsis)	L1
1415934_at	COPS8	COP9 constitutive photomorphogenic homolog subunit 8 (Arabidopsis)	L1
1449660_s_at	CORO1C	coronin, actin binding protein, 1C	L1
1426693_x_at	COX15	COX15 homolog, cytochrome c oxidase assembly protein (yeast)	L1
1418709_at	COX7A1	cytochrome c oxidase subunit VIIa polypeptide 1 (muscle)	
1449218_at	Cox8b	cytochrome c oxidase, subunit VIIIb	
1417496_at	CP	ceruloplasmin (ferroxidase)	
1433439_at	CPNE1	copine I	L1
1423964_at	CPSF3L	cleavage and polyadenylation specific factor 3-like	L1
1448901_at	CPXM1	carboxypeptidase X (M14 family), member 1	L1
1456042_s_at	CRAMP1L	Crm, cramped-like (Drosophila)	
1431930_x_at	CRLS1	cardiolipin synthase 1	L1

1448592_at	CRTAP	cartilage associated protein	L1
1424660_s_at	CRTC2	CREB regulated transcription coactivator 2	L1
1429373_x_at	CRTC2	CREB regulated transcription coactivator 2	L1
1449932_at	CSNK1D	casein kinase 1, delta	L1
1460646_at	CSNK2A2	casein kinase 2, alpha prime polypeptide	L1
1422140_at	Csprs (includes others)	component of Sp100-rs	
1415702_a_at	CTBP1	C-terminal binding protein 1	L1
1421315_s_at	CTTN	cortactin	L1
1427620_at	CUL9	cullin 9	
1426455_at	CWC27	CWC27 spliceosome-associated protein homolog (S. cerevisiae)	
1427321_s_at	CXADR	coxsackie virus and adenovirus receptor	L1
1448823_at	Cxcl12	chemokine (C-X-C motif) ligand 12	
1448859_at	CXCL13	chemokine (C-X-C motif) ligand 13	L1
1417767_at	CYB5B	cytochrome b5 type B (outer mitochondrial membrane)	L1
1424048_a_at	CYB5R1	cytochrome b5 reductase 1	L1
1426307_at	CYB5R4	cytochrome b5 reductase 4	L1
1449406_at	CYHR1	cysteine/histidine-rich 1	L1
1422217_a_at	CYP1A1	cytochrome P450, family 1, subfamily A, polypeptide 1	L1
1460011_at	CYP26B1	cytochrome P450, family 26, subfamily B, polypeptide 1	
1425645_s_at	CYP2B6	cytochrome P450, family 2, subfamily B, polypeptide 6	L1
1419040_at	Cyp2d22	cytochrome P450, family 2, subfamily d, polypeptide 22	L1
1458126_at	D030041H20Rik	RIKEN cDNA D030041H20 gene	
1447526_at	D5Ertd255e	DNA segment, Chr 5, ERATO Doi 255, expressed	
1433558_at	DAB2IP	DAB2 interacting protein	L1
1417937_at	DACT1	dapper, antagonist of beta-catenin, homolog 1 (Xenopus laevis)	

1426778_at	DAG1	dystroglycan 1 (dystrophin-associated glycoprotein 1)	L1
1423790_at	DAP	death-associated protein	L1
1438727_at	DCAF10	DDB1 and CUL4 associated factor 10	L1
1430059_at	DCAF12	DDB1 and CUL4 associated factor 12	L1
1424777_at	DCAF4	DDB1 and CUL4 associated factor 4	L1
1420526_at	DCBLD2	discoidin, CUB and LCCL domain containing 2	L1
1433606_at	DCP1A	DCP1 decapping enzyme homolog A (S. cerevisiae)	L1
1422521_at	DCTN1	dynactin 1	L1
1420862_at	DCTN4	dynactin 4 (p62)	L1
1429094_at	DDI2	DNA-damage inducible 1 homolog 2 (S. cerevisiae)	
1416071_at	DDX18	DEAD (Asp-Glu-Ala-Asp) box polypeptide 18	L1
1416751_a_at	DDX20	DEAD (Asp-Glu-Ala-Asp) box polypeptide 20	L1
1423643_at	DDX39A	DEAD (Asp-Glu-Ala-Asp) box polypeptide 39A	L1
1423814_at	DDX41	DEAD (Asp-Glu-Ala-Asp) box polypeptide 41	L1
1447789_x_at	DDX6	DEAD (Asp-Glu-Ala-Asp) box helicase 6	L1
1451777_at	DDX60	DEAD (Asp-Glu-Ala-Asp) box polypeptide 60	L1
1423345_at	DEGS1	delta(4)-desaturase, sphingolipid 1	L1
1430406_at	DENND1B	DENN/MADD domain containing 1B	L1
1428622_at	DEPTOR	DEP domain containing MTOR-interacting protein	L1
1443579_s_at	DEPTOR	DEP domain containing MTOR-interacting protein	L1
1451348_at	DEPTOR	DEP domain containing MTOR-interacting protein	L1
1433563_s_at	DERL1	derlin 1	L1

1437051_at	DFFB	DNA fragmentation factor, 40kDa, beta polypeptide (caspase-activated DNase)	
1426738_at	DGKZ	diacylglycerol kinase, zeta	L1
1434004_at	DHPS	deoxyhypusine synthase	L1
1416140_a_at	DHX30	DEAH (Asp-Glu-Ala-His) box polypeptide 30	L1
1452693_at	DHX35	DEAH (Asp-Glu-Ala-His) box polypeptide 35	L1
1420769_at	DHX58	DEXH (Asp-Glu-X-His) box polypeptide 58	
1454654_at	DIRC2	disrupted in renal carcinoma 2	L1
1430161_at	DLST	dihydrolipoamide S-succinyltransferase (E2 component of 2-oxo-glutarate complex)	
1437775_at	DLST	dihydrolipoamide S-succinyltransferase (E2 component of 2-oxo-glutarate complex)	L1
1421544_at	DNAH11	dynein, axonemal, heavy chain 11	L1
1416756_at	DNAJB1	DnaJ (Hsp40) homolog, subfamily B, member 1	L1
1447055_at	DNAJC11	DnaJ (Hsp40) homolog, subfamily C, member 11	L1
1445337_at	DNAJC13	DnaJ (Hsp40) homolog, subfamily C, member 13	L1
1420220_x_at	DNAJC21	DnaJ (Hsp40) homolog, subfamily C, member 21	L1
1451389_at	DNAJC24	DnaJ (Hsp40) homolog, subfamily C, member 24	L1
1435117_a_at	DNAJC27	DnaJ (Hsp40) homolog, subfamily C, member 27	
1425035_s_at	DNMT3L	DNA (cytosine-5-)-methyltransferase 3-like	
1427881_at	DNTTIP2	deoxynucleotidyltransferase, terminal, interacting protein 2	L1
1427240_at	DOCK6	dedicator of cytokinesis 6	L1
1426739_at	DONSON	downstream neighbor of SON	L1

1448549_a_at	DPAGT1	dolichyl-phosphate (UDP-N-acetylglucosamine) N-acetylglucosaminephosphotransferase 1 (GlcNAc-1-P transferase)	L1
1428656_at	DROSHA	drosha, ribonuclease type III	L1
1421276_a_at	DST	dystonin	L1
1417124_at	DSTN	destrin (actin depolymerizing factor)	L1
1438096_a_at	DTYMK	deoxythymidylate kinase (thymidylate kinase)	
1424066_at	DUS3L	dihydrouridine synthase 3-like (S. cerevisiae)	L1
1459805_x_at	DUS3L	dihydrouridine synthase 3-like (S. cerevisiae)	L1
1450698_at	DUSP2	dual specificity phosphatase 2	L1
1428834_at	DUSP4	dual specificity phosphatase 4	L1
1450978_at	DVL1	dishevelled, dsh homolog 1 (Drosophila)	L1
1434723_at	E130307A14Rik	RIKEN cDNA E130307A14 gene	
1417444_at	E2F5	E2F transcription factor 5, p130-binding	L1
1433555_at	EAF1	ELL associated factor 1	L1
1416302_at	EBF1	early B-cell factor 1	L1
1455741_a_at	ECE1	endothelin converting enzyme 1	L1
1419638_at	EFNB2	ephrin-B2	L1
1416131_s_at	EFR3A	EFR3 homolog A (S. cerevisiae)	L1
1416557_a_at	EFTUD2	elongation factor Tu GTP binding domain containing 2	L1
1438835_a_at	EFTUD2	elongation factor Tu GTP binding domain containing 2	L1
1434647_at	EGFLAM	EGF-like, fibronectin type III and laminin G domains	L1
1418649_at	EGLN3	egl nine homolog 3 (C. elegans)	
1449852_a_at	EHD4	EH-domain containing 4	L1
1460692_at	EHMT2	euchromatic histone-lysine N-methyltransferase 2	L1
1429350_at	EID3	EP300 interacting inhibitor of differentiation 3	L1
1422005_at	EIF2AK2	eukaryotic translation initiation factor 2-alpha kinase 2	
1441063_at	EIF2C3	eukaryotic translation initiation factor 2C, 3	

1455575_at	EIF4EBP2	eukaryotic translation initiation factor 4E binding protein 2	L1
1426833_at	EIF4G3	eukaryotic translation initiation factor 4 gamma, 3	L1
1451521_x_at	EIF4H	eukaryotic translation initiation factor 4H	L1
1434605_at	EIF5B	eukaryotic translation initiation factor 5B	
1428045_a_at	ELF2	E74-like factor 2 (ets domain transcription factor)	
1456098_a_at	ELMO2	engulfment and cell motility 2	L1
1434489_at	ELMO3	engulfment and cell motility 3	
1415774_at	ELP2	elongator acetyltransferase complex subunit 2	L1
1448882_at	EMC6	ER membrane protein complex subunit 6	L1
1435264_at	EMILIN2	elastin microfibril interfacer 2	L1
1423705_at	ENOPH1	enolase-phosphatase 1	L1
1441630_at	EP400	E1A binding protein p400	
1426860_at	EP400	E1A binding protein p400	L1
1447349_s_at	EP400	E1A binding protein p400	L1
1459619_at	EPB41L2	erythrocyte membrane protein band 4.1-like 2	L1
1419062_at	EPB41L3	erythrocyte membrane protein band 4.1-like 3	
1421815_at	EPDR1	ependymin related protein 1 (zebrafish)	L1
1428419_at	EPG5	ectopic P-granules autophagy protein 5 homolog (C. elegans)	
1421151_a_at	EPHA2	EPH receptor A2	
1452158_at	EPRS	glutamyl-prolyl-tRNA synthetase	L1
1441604_at	ESD	esterase D	L1
1451142_at	EXOC1	exocyst complex component 1	L1
1422684_a_at	EXOC4	exocyst complex component 4	
1460679_at	EXOSC4	exosome component 4	L1
1418290_a_at	EZH1	enhancer of zeste homolog 1 (Drosophila)	
1437308_s_at	F2R	coagulation factor II (thrombin) receptor	L1
1426893_at	FAM102A	family with sequence similarity 102, member A	L1

1436229_at	FAM126B	family with sequence similarity 126, member B	L1
1455793_at	FAM149A	family with sequence similarity 149, member A	L1
1434269_at	FAM149B1	family with sequence similarity 149, member B1	L1
1435686_at	FAM149B1	family with sequence similarity 149, member B1	L1
1453284_at	FAM151B	family with sequence similarity 151, member B	L1
1417402_at	Fam165b	family with sequence similarity 165, member B	L1
1419170_at	FAM174A	family with sequence similarity 174, member A	L1
1417688_at	FAM20C	family with sequence similarity 20, member C	
1436150_at	FAM219A	family with sequence similarity 219, member A	
1433588_at	FAM21A/FAM21C	family with sequence similarity 21, member C	L1
1454644_at	FAM40A	family with sequence similarity 40, member A	L1
1443747_at	FAM48A	family with sequence similarity 48, member A	L1
1428874_at	FAM96B	family with sequence similarity 96, member B	L1
1452280_at	FARP1	FERM, RhoGEF (ARHGEF) and pleckstrin domain protein 1 (chondrocyte-derived)	L1
1460251_at	FAS	Fas (TNF receptor superfamily, member 6)	L1
1460635_at	FASTK	Fas-activated serine/threonine kinase	L1
1459749_s_at	FAT4	FAT tumor suppressor homolog 4 (Drosophila)	
1452860_at	FBXL17	F-box and leucine-rich repeat protein 17	L1
1418419_at	FBXL20	F-box and leucine-rich repeat protein 20	L1
1448747_at	FBXO32	F-box protein 32	
1427121_at	FBXO4	F-box protein 4	L1
1418323_at	FEM1B	fem-1 homolog b (C. elegans)	L1

1455337_at	FGD4	FYVE, RhoGEF and PH domain containing 4	
1439959_at	FGF11	fibroblast growth factor 11	
1424050_s_at	FGFR1	fibroblast growth factor receptor 1	
1451912_a_at	FGFRL1	fibroblast growth factor receptor-like 1	L1
1416044_at	FLII	flightless I homolog (Drosophila)	L1
1448189_a_at	FLII	flightless I homolog (Drosophila)	L1
1442107_at	FLNB	filamin B, beta	
1417544_a_at	FLOT2	flotillin 2	L1
1434414_at	FOXRED2	FAD-dependent oxidoreductase domain containing 2	
1423681_at	FTSJD2	FtsJ methyltransferase domain containing 2	L1
1437544_at	FUBP1	far upstream element (FUSE) binding protein 1	
1419761_a_at	GABPB1	GA binding protein transcription factor, beta subunit 1	
1440868_at	GABPB2	GA binding protein transcription factor, beta subunit 2	
1418194_at	GALNT10	UDP-N-acetyl-alpha-D-galactosamine:polypeptide N-acetylgalactosaminyltransferase 10 (GalNAc-T10)	L1
1422558_at	GAMT	guanidinoacetate N-methyltransferase	
1415787_at	GANAB	glucosidase, alpha; neutral AB	L1
1436385_at	GANC	glucosidase, alpha; neutral C	L1
1425599_a_at	GATAD1	GATA zinc finger domain containing 1	L1
1423992_at	GATAD2A	GATA zinc finger domain containing 2A	L1
1419484_a_at	GBAS	glioblastoma amplified sequence	
1420654_a_at	GBE1	glucan (1,4-alpha-), branching enzyme 1	L1
1418627_at	GCLM	glutamate-cysteine ligase, modifier subunit	L1
1439050_at	GCLM	glutamate-cysteine ligase, modifier subunit	L1
1455180_at	GCOM1	GRINL1A complex locus 1	L1

1423554_at	GGCX	gamma-glutamyl carboxylase	L1
1419595_a_at	GGH	gamma-glutamyl hydrolase (conjugase, folylpolygammaglutamyl hydrolase)	L1
1419502_at	GHDC	GH3 domain containing	L1
1428713_s_at	GINS2	GINS complex subunit 2 (Psf2 homolog)	
1447787_x_at	GJC1	gap junction protein, gamma 1, 45kDa	L1
1430385_a_at	GLB1L	galactosidase, beta 1-like	L1
1433728_at	GLB1L2	galactosidase, beta 1-like 2	
1451972_at	GLCCI1	glucocorticoid induced transcript 1	L1
1452033_at	GLE1	GLE1 RNA export mediator homolog (yeast)	L1
1428270_at	GLT8D1	glycosyltransferase 8 domain containing 1	L1
1419443_at	Gm10094/Sap18	Sin3-associated polypeptide 18	L1
1436265_at	Gm10419	predicted gene 10419	L1
1430285_at	Gm14057	Ppp1r14c pseudogene	
1456547_at	Gm19313	predicted gene, 19313	L1
1441233_at	Gm19858	predicted gene, 19858	L1
1420304_x_at	Gm19974	predicted gene, 19974	L1
1460021_at	Gm6658	predicted gene 6658	
1446470_at	Gm9799	predicted gene 9799	L1
1447246_at	Gm9873	predicted gene 9873	L1
1417069_a_at	GMFB	glia maturation factor, beta	
1434254_at	GNA11	guanine nucleotide binding protein (G protein), alpha 11 (Gq class)	L1
1426579_at	GNL2	guanine nucleotide binding protein-like 2 (nucleolar)	L1
1437566_at	GNL2	guanine nucleotide binding protein-like 2 (nucleolar)	L1
1423157_at	GNPNAT1	glucosamine-phosphate N-acetyltransferase 1	L1
1436526_at	GNPTG	N-acetylglucosamine-1-phosphate transferase, gamma subunit	L1
1424385_at	GON4L	gon-4-like (C. elegans)	L1
1458365_at	GORAB	golgin, RAB6-interacting	L1

1451671_at	GORASP1	golgi reassembly stacking protein 1, 65kDa	L1
1450970_at	GOT1	glutamic-oxaloacetic transaminase 1, soluble (aspartate aminotransferase 1)	L1
1437417_s_at	GPC6	glypican 6	L1
1434344_at	GPKOW	G patch domain and KOW motifs	L1
1418379_s_at	GPR124	G protein-coupled receptor 124	L1
1417245_at	GPR180	G protein-coupled receptor 180	L1
1415699_a_at	GPS1	G protein pathway suppressor 1	L1
1441894_s_at	GRASP	GRP1 (general receptor for phosphoinositides 1)-associated scaffold protein	
1424030_at	GRHL1	grainyhead-like 1 (Drosophila)	
1427046_at	GRHL2	grainyhead-like 2 (Drosophila)	L1
1420563_at	GRIA3	glutamate receptor, ionotropic, AMPA 3	
1451672_at	GRK6	G protein-coupled receptor kinase 6	L1
1433457_s_at	GRSF1	G-rich RNA sequence binding factor 1	L1
1439931_at	GSK3B	glycogen synthase kinase 3 beta	
1434568_at	GTF3C6	general transcription factor IIIC, polypeptide 6, alpha 35kDa	L1
1416690_at	GTPBP2	GTP binding protein 2	L1
1423878_at	GYPC	glycophorin C (Gerbich blood group)	L1
1424771_at	H2AFJ	H2A histone family, member J	L1
1451229_at	HDAC11	histone deacetylase 11	L1
1448928_at	HDAC6	histone deacetylase 6	L1
1449615_s_at	HDLBP	high density lipoprotein binding protein	L1
1435013_at	HEATR2	HEAT repeat containing 2	L1
1437970_at	HEATR2	HEAT repeat containing 2	L1
1424141_at	HECTD1	HECT domain containing E3 ubiquitin protein ligase 1	L1
1433524_at	HECTD4	HECT domain containing E3 ubiquitin protein ligase 4	
1434060_at	HERC1	HECT and RLD domain containing E3 ubiquitin protein ligase family member 1	L1

1444228_s_at	HERC2	HECT and RLD domain containing E3 ubiquitin protein ligase 2	
1434844_at	HEXDC	hexosaminidase (glycosyl hydrolase family 20, catalytic domain) containing	
1448359_a_at	Higd1a	HIG1 domain family, member 1A	L1
1448512_at	HILS1	histone linker H1 domain, spermatid-specific 1, pseudogene	L1
1434557_at	HIP1	huntingtin interacting protein 1	L1
1429566_a_at	HIPK2	homeodomain interacting protein kinase 2	L1
1456022_at	HIPK2	homeodomain interacting protein kinase 2	L1
1419191_at	HIPK3	homeodomain interacting protein kinase 3	L1
1422948_s_at	Hist2h4 (includes others)	histone cluster 2, H4	L1
1458802_at	HIVEP3	human immunodeficiency virus type I enhancer binding protein 3	
1417637_a_at	HMG20B	high mobility group 20B	L1
1448239_at	HMOX1	heme oxygenase (decycling) 1	L1
1452030_a_at	HNRNPR	heterogeneous nuclear ribonucleoprotein R	L1
1419350_at	HOOK2	hook homolog 2 (Drosophila)	
1439196_at	HOOK3	hook homolog 3 (Drosophila)	L1
1427362_x_at	HOXC6	homeobox C6	L1
1450647_at	HPS3	Hermansky-Pudlak syndrome 3	
1434677_at	HPS5	Hermansky-Pudlak syndrome 5	L1
1449038_at	HSD11B1	hydroxysteroid (11-beta) dehydrogenase 1	
1424622_at	HSF1	heat shock transcription factor 1	L1
1434051_s_at	HSPA12A	heat shock 70kDa protein 12A	L1
1458385_at	HSPA4L	heat shock 70kDa protein 4-like	
1447824_x_at	HSPA5	heat shock 70kDa protein 5 (glucose-regulated protein, 78kDa)	L1
1427029_at	HTRA3	HtrA serine peptidase 3	L1
1415703_at	HUWE1	HECT, UBA and WWE domain containing 1, E3 ubiquitin protein ligase	L1

1443183_at	HUWE1	HECT, UBA and WWE domain containing 1, E3 ubiquitin protein ligase	L1
1452154_at	IARS	isoleucyl-tRNA synthetase	L1
1423259_at	ID4	inhibitor of DNA binding 4, dominant negative helix-loop-helix protein	
1441271_at	IDH3B	isocitrate dehydrogenase 3 (NAD+) beta	L1
1454628_at	IFFO1	intermediate filament family orphan 1	L1
1451462_a_at	IFNAR2	interferon (alpha, beta and omega) receptor 2	L1
1424648_at	IFT27	intraflagellar transport 27 homolog (Chlamydomonas)	L1
1434299_x_at	IFT27	intraflagellar transport 27 homolog (Chlamydomonas)	L1
1439764_s_at	IGF2BP2	insulin-like growth factor 2 mRNA binding protein 2	
1440979_at	IGF2R	insulin-like growth factor 2 receptor	L1
1422826_at	IGFALS	insulin-like growth factor binding protein, acid labile subunit	L1
1458268_s_at	IGFBP3	insulin-like growth factor binding protein 3	L1
1437804_at	IGFBP7	insulin-like growth factor binding protein 7	
1423584_at	IGFBP7	insulin-like growth factor binding protein 7	L1
1425763_x_at	IGHA1	immunoglobulin heavy constant alpha 1	
1427329_a_at	IGHM	immunoglobulin heavy constant mu	L1
1427351_s_at	IGHM	immunoglobulin heavy constant mu	L1
1424305_at	IGJ	immunoglobulin J polypeptide, linker protein for immunoglobulin alpha and mu polypeptides	
1452557_a_at	Igk	immunoglobulin kappa chain complex	

1429065_at	IKBIP	IKBKB interacting protein	L1
1418219_at	IL15	interleukin 15	L1
1448681_at	IL15RA	interleukin 15 receptor, alpha	
1435714_x_at	IL17D	interleukin 17D	L1
1420904_at	IL17RA	interleukin 17 receptor A	L1
1420905_at	IL17RA	interleukin 17 receptor A	L1
1449942_a_at	ILK	integrin-linked kinase	L1
1454658_at	ILVBL	ilvB (bacterial acetolactate synthase)-like	L1
1423229_at	INPP5E	inositol polyphosphate-5-phosphatase, 72 kDa	L1
1442100_at	INPP5F	inositol polyphosphate-5-phosphatase F	L1
1460394_a_at	INPPL1	inositol polyphosphate phosphatase-like 1	L1
1426943_at	INTS1	integrator complex subunit 1	L1
1448668_a_at	IRAK1	interleukin-1 receptor-associated kinase 1	L1
1435040_at	IRAK3	interleukin-1 receptor-associated kinase 3	L1
1430316_at	ISPD	isoprenoid synthase domain containing	
1423837_at	IST1	increased sodium tolerance 1 homolog (yeast)	L1
1423838_s_at	IST1	increased sodium tolerance 1 homolog (yeast)	L1
1415977_at	ISYNA1	inositol-3-phosphate synthase 1	L1
1456498_at	ITGA4	integrin, alpha 4 (antigen CD49D, alpha 4 subunit of VLA-4 receptor)	
1450029_s_at	ITGA9	integrin, alpha 9	L1
1417279_at	ITPR1	inositol 1,4,5-trisphosphate receptor, type 1	L1
1427287_s_at	ITPR2	inositol 1,4,5-trisphosphate receptor, type 2	L1
1425899_a_at	ITSN1	intersectin 1 (SH3 domain protein)	L1
1433805_at	JAK1	Janus kinase 1	L1
1460577_at	JMY	junction mediating and regulatory protein, p53 cofactor	L1
1433742_at	KANK1	KN motif and ankyrin repeat domains 1	L1

1438400_at	KANSL3	KAT8 regulatory NSL complex subunit 3	L1
1433433_at	KAT7	K(lysine) acetyltransferase 7	L1
1423179_at	KCNB1	potassium voltage-gated channel, Shab-related subfamily, member 1	L1
1418142_at	KCNJ8	potassium inwardly-rectifying channel, subfamily J, member 8	L1
1449158_at	KCNK2	potassium channel, subfamily K, member 2	
1425341_at	KCNK3	potassium channel, subfamily K, member 3	L1
1440355_at	Kctd12b	potassium channel tetramerisation domain containing 12b	L1
1435525_at	KCTD17	potassium channel tetramerisation domain containing 17	L1
1448302_at	KCTD20	potassium channel tetramerisation domain containing 20	L1
1436811_at	KCTD3	potassium channel tetramerisation domain containing 3	L1
1439691_at	KIAA0232	KIAA0232	
1454721_at	KIAA0317	KIAA0317	L1
1427978_at	KIAA0494	KIAA0494	L1
1427319_at	KIAA1033	KIAA1033	
1457678_at	KIAA1468	KIAA1468	
1424913_at	KIAA1737	KIAA1737	L1
1447853_x_at	KIF13A	kinesin family member 13A	
1423995_at	KIF1B	kinesin family member 1B	L1
1417395_at	KLF4	Kruppel-like factor 4 (gut)	L1
1418280_at	KLF6	Kruppel-like factor 6	
1460678_at	KLHDC2	kelch domain containing 2	L1
1455910_at	KLHDC3	kelch domain containing 3	
1428081_at	KLHL21	kelch-like 21 (Drosophila)	L1
1426481_at	KLHL22	kelch-like 22 (Drosophila)	L1
1417975_at	KPNA4	karyopherin alpha 4 (importin alpha 3)	
1416925_at	KPNB1	karyopherin (importin) beta 1	L1

1435472_at	KREMEN1	kringle containing transmembrane protein 1	L1
1428084_at	KRR1	KRR1, small subunit (SSU) processome component, homolog (yeast)	L1
1427352_at	KRT79	keratin 79	
1460185_at	KRT85	keratin 85	L1
1416106_at	KTI12	KTI12 homolog, chromatin associated (S. cerevisiae)	L1
1427073_at	LACE1	lactation elevated 1	L1
1424808_at	LAMA4	laminin, alpha 4	
1416513_at	LAMB2	laminin, beta 2 (laminin S)	L1
1448718_at	LAMTOR1	late endosomal/lysosomal adaptor, MAPK and MTOR activator 1	
1428310_at	LARP7	La ribonucleoprotein domain family, member 7	L1
1456309_x_at	LASP1	LIM and SH3 protein 1	
1460173_at	LASP1	LIM and SH3 protein 1	
1417044_at	LCMT1	leucine carboxyl methyltransferase 1	L1
1452752_at	LEPRE1	leucine proline-enriched proteoglycan (leprecan) 1	
1421217_a_at	LGALS9	lectin, galactoside-binding, soluble, 9	L1
1448883_at	LGMN	legumain	L1
1425094_a_at	LHX6	LIM homeobox 6	L1
1421207_at	LIF	leukemia inhibitory factor	L1
1425107_a_at	LIFR	leukemia inhibitory factor receptor alpha	L1
1435727_s_at	LIMA1	LIM domain and actin binding 1	
1450629_at	LIMA1	LIM domain and actin binding 1	
1422499_at	LIMA1	LIM domain and actin binding 1	L1
1416869_x_at	LIME1	Lck interacting transmembrane adaptor 1	L1
1448500_a_at	LIME1	Lck interacting transmembrane adaptor 1	L1
1448531_at	LMNB2	lamin B2	L1
1420981_a_at	LMO4	LIM domain only 4	L1
1438432_at	LNPEP	leucyl/cystinyl aminopeptidase	
1446154_at	LOC100047123	uncharacterized LOC100047123	L1
1455465_at	LOC100505262	uncharacterized LOC100505262	

1427820_at	LOC100861696	uncharacterized LOC100861696	
1447975_a_at	LOC545261	uncharacterized LOC545261	L1
1442430_at	LOC646498	uncharacterized LOC646498	L1
1460178_at	LONP2	lon peptidase 2, peroxisomal	L1
1448606_at	LPAR1	lysophosphatidic acid receptor 1	
1431056_a_at	LPL	lipoprotein lipase	L1
1454756_at	LRCH3	leucine-rich repeats and calponin homology (CH) domain containing 3	L1
1416836_at	LRP10	low density lipoprotein receptor-related protein 10	L1
1433536_at	LRP11	low density lipoprotein receptor-related protein 11	L1
1451022_at	LRP6	low density lipoprotein receptor-related protein 6	
1424353_at	LRPPRC	leucine-rich pentatricopeptide repeat containing	L1
1435648_at	LRRN4	leucine rich repeat neuronal 4	L1
1429176_at	LRSAM1	leucine rich repeat and sterile alpha motif containing 1	L1
1425548_a_at	Lst1	leukocyte specific transcript 1	L1
1426813_at	LTV1	LTV1 homolog (S. cerevisiae)	L1
1452708_a_at	LUC7L	LUC7-like (S. cerevisiae)	
1427087_at	LUC7L2	LUC7-like 2 (S. cerevisiae)	
1428384_at	LURAP1L	leucine rich adaptor protein 1-like	L1
1423547_at	Lyz1/Lyz2	lysozyme 2	L1
1450927_at	LZTR1	leucine-zipper-like transcription regulator 1	L1
1423031_at	MAEA	macrophage erythroblast attacher	L1
1451716_at	MAFB	v-maf musculoaponeurotic fibrosarcoma oncogene homolog B (avian)	L1
1426306_a_at	MAGED2	melanoma antigen family D, 2	L1
1422431_at	MAGEE1	melanoma antigen family E, 1	L1
1454855_at	MAGI2	membrane associated guanylate kinase, WW and PDZ domain containing 2	L1
1418188_a_at	MALAT1	metastasis associated lung adenocarcinoma transcript 1 (non-protein coding)	

1418189_s_at	MALAT1	metastasis associated lung adenocarcinoma transcript 1 (non-protein coding)	
1452378_at	MALAT1	metastasis associated lung adenocarcinoma transcript 1 (non-protein coding)	
1438403_s_at	MALAT1	metastasis associated lung adenocarcinoma transcript 1 (non-protein coding)	L1
1417110_at	MAN1A1	mannosidase, alpha, class 1A, member 1	
1417111_at	MAN1A1	mannosidase, alpha, class 1A, member 1	L1
1456534_at	MAN1A2	mannosidase, alpha, class 1A, member 2	
1428024_at	MAP1LC3B	microtubule-associated protein 1 light chain 3 beta	L1
1451714_a_at	MAP2K3	mitogen-activated protein kinase kinase 3	L1
1425512_at	MAP2K7	mitogen-activated protein kinase kinase 7	L1
1439467_at	MAP4	microtubule-associated protein 4	
1422615_at	MAP4K4	mitogen-activated protein kinase kinase kinase kinase 4	L1
1422550_a_at	MAP6	microtubule-associated protein 6	
1457316_at	MAP6	microtubule-associated protein 6	
1425679_a_at	MAPK8IP1	mitogen-activated protein kinase 8 interacting protein 1	
1425975_a_at	MAPK8IP3	mitogen-activated protein kinase 8 interacting protein 3	L1
1426648_at	MAPKAPK2	mitogen-activated protein kinase-activated protein kinase 2	L1
1437494_at	MAPKAPK3	mitogen-activated protein kinase-activated protein kinase 3	L1
1417016_at	MAPKAPK5	mitogen-activated protein kinase-activated protein kinase 5	L1
1422765_at	MAPRE1	microtubule-associated protein, RP/EB family, member 1	

1428820_at	MAPRE1	microtubule-associated protein, RP/EB family, member 1	
1442553_at	MAPRE2	microtubule-associated protein, RP/EB family, member 2	L1
1419442_at	MATN2	matrilin 2	L1
1440299_at	MB21D1	Mab-21 domain containing 1	
1441388_at	MBD2	methyl-CpG binding domain protein 2	L1
1437704_at	MBLAC2	metallo-beta-lactamase domain containing 2	L1
1457924_at	MBNL1	muscleblind-like splicing regulator 1	L1
1448711_at	MCM3AP	minichromosome maintenance complex component 3 associated protein	L1
1455117_at	MCM9	minichromosome maintenance complex component 9	L1
1424348_at	MCMBP	minichromosome maintenance complex binding protein	L1
1416671_a_at	MCOLN1	mucolipin 1	L1
1416535_at	MCRS1	microspherule protein 1	L1
1451058_at	Mcts2	malignant T cell amplified sequence 2	L1
1458223_at	MDM4	Mdm4 p53 binding protein homolog (mouse)	L1
1426572_at	ME2	malic enzyme 2, NAD(+)-dependent, mitochondrial	L1
1425585_at	MED12	mediator complex subunit 12	L1
1436904_at	MED13	mediator complex subunit 13	
1448435_at	MED15	mediator complex subunit 15	L1
1425473_at	MED17	mediator complex subunit 17	L1
1417129_a_at	MEIS2	Meis homeobox 2	L1
1428582_at	METTL10	methyltransferase like 10	L1
1452950_at	METTL15	methyltransferase like 15	L1
1434749_at	METTL25	methyltransferase like 25	
1417710_at	METTL9	methyltransferase like 9	L1
1451572_a_at	MFF	mitochondrial fission factor	L1
1452335_at	MFSD8	major facilitator superfamily domain containing 8	L1
1452037_at	MGAT2	mannosyl (alpha-1,6-)-glycoprotein beta-1,2-N-acetylglucosaminyltransferase	L1

1431331_at	MGLL	monoglyceride lipase	
1454645_at	MGRN1	mahogunin ring finger 1, E3 ubiquitin protein ligase	L1
1459984_at	MIA3	melanoma inhibitory activity family, member 3	
1438726_at	MICAL2	microtubule associated monoxygenase, calponin and LIM domain containing 2	L1
1452916_at	MLL4	myeloid/lymphoid or mixed-lineage leukemia 4	L1
1441456_at	MMP24	matrix metallopeptidase 24 (membrane-inserted)	L1
1448291_at	MMP9	matrix metallopeptidase 9 (gelatinase B, 92kDa gelatinase, 92kDa type IV collagenase)	L1
1455099_at	MOGAT2	monoacylglycerol O-acyltransferase 2	
1449476_at	MOK	MOK protein kinase	
1454158_at	MPP7	membrane protein, palmitoylated 7 (MAGUK p55 subfamily member 7)	
1451858_at	MRGPRX3	MAS-related GPR, member X3	L1
1450866_a_at	MRPL17	mitochondrial ribosomal protein L17	L1
1416948_at	MRPL23	mitochondrial ribosomal protein L23	
1427158_at	MRPS30	mitochondrial ribosomal protein S30	L1
1452111_at	MRPS35	mitochondrial ribosomal protein S35	L1
1421230_a_at	MSI2	musashi homolog 2 (Drosophila)	L1
1434481_at	Msl1	male-specific lethal 1 homolog (Drosophila)	L1
1429601_x_at	MTHFD2L	methylenetetrahydrofolate dehydrogenase (NADP+ dependent) 2-like	
1429600_at	MTHFD2L	methylenetetrahydrofolate dehydrogenase (NADP+ dependent) 2-like	L1
1419387_s_at	MUC13	mucin 13, cell surface associated	L1
1422574_at	MXD4	MAX dimerization protein 4	
1434379_at	MXD4	MAX dimerization protein 4	L1

1434274_at	MYCBP2	MYC binding protein 2, E3 ubiquitin protein ligase	
1450651_at	MYO10	myosin X	L1
1451183_at	MYO19	myosin XIX	L1
1420426_at	MYO7B	myosin VIIB	L1
1436307_at	MYO9A	myosin IXA	L1
1417707_at	N4BP2L1	NEDD4 binding protein 2-like 1	
1452914_at	N4BP2L1	NEDD4 binding protein 2-like 1	
1433766_at	NAA25	N(alpha)-acetyltransferase 25, NatB auxiliary subunit	L1
1415721_a_at	NAA60	N(alpha)-acetyltransferase 60, NatF catalytic subunit	L1
1448781_at	NAB1	NGFI-A binding protein 1 (EGR1 binding protein 1)	L1
1417153_at	NACC2	NACC family member 2, BEN and BTB (POZ) domain containing	L1
1429582_at	NACC2	NACC family member 2, BEN and BTB (POZ) domain containing	L1
1430359_a_at	NADSYN1	NAD synthetase 1	L1
1448746_at	NBN	nibrin	L1
1423699_at	NCAPH2	non-SMC condensin II complex, subunit H2	L1
1444086_at	NCKAP5	NCK-associated protein 5	L1
1418594_a_at	NCOA1	nuclear receptor coactivator 1	L1
1435234_at	NCOA2	nuclear receptor coactivator 2	
1415923_at	NDN	necdin homolog (mouse)	L1
1450976_at	NDRG1	N-myc downstream regulated 1	L1
1417664_a_at	NDRG3	NDRG family member 3	L1
1425850_a_at	NEK6	NIMA (never in mitosis gene a)-related kinase 6	
1438999_a_at	NFAT5	nuclear factor of activated T-cells 5, tonicity-responsive	
1457117_at	NFE2L2	nuclear factor (erythroid-derived 2)-like 2	L1
1427680_a_at	NFIB	nuclear factor I/B	L1
1423493_a_at	NFIX	nuclear factor I/X (CCAAT-binding transcription factor)	L1
1436363_a_at	NFIX	nuclear factor I/X (CCAAT-binding transcription factor)	L1
1436364_x_at	NFIX	nuclear factor I/X (CCAAT-binding transcription factor)	L1

1427705_a_at	NFKB1	nuclear factor of kappa light polypeptide gene enhancer in B-cells 1	
1446718_at	NFKBIB	nuclear factor of kappa light polypeptide gene enhancer in B-cells inhibitor, beta	L1
1434753_at	NFRKB	nuclear factor related to kappaB binding protein	L1
1423516_a_at	NID2	nidogen 2 (osteonidogen)	
1453345_at	NIPAL1	NIPA-like domain containing 1	
1442103_at	NIPBL	Nipped-B homolog (Drosophila)	
1423249_at	NKTR	natural killer-tumor recognition sequence	
1424981_at	NLN	neurolysin (metallopeptidase M3 family)	L1
1425646_at	NMRK1	nicotinamide riboside kinase 1	
1418222_at	NOA1	nitric oxide associated 1	L1
1423211_at	NOP10	NOP10 ribonucleoprotein homolog (yeast)	
1423210_a_at	NOP10	NOP10 ribonucleoprotein homolog (yeast)	L1
1421964_at	NOTCH3	notch 3	L1
1423086_at	NPC1	Niemann-Pick disease, type C1	L1
1427191_at	NPR2	natriuretic peptide receptor B/guanylate cyclase B (atrionatriuretic peptide receptor B)	L1
1433548_at	NPRL3	nitrogen permease regulator-like 3 (S. cerevisiae)	L1
1426464_at	NR1D1	nuclear receptor subfamily 1, group D, member 1	
1418605_at	NR2C1	nuclear receptor subfamily 2, group C, member 1	L1
1420410_at	NR5A2	nuclear receptor subfamily 5, group A, member 2	
1417985_at	NRARP	NOTCH-regulated ankyrin repeat protein	L1
1423864_at	NRBP1	nuclear receptor binding protein 1	L1
1418469_at	NRIP1	nuclear receptor interacting protein 1	

1449089_at	NRIP1	nuclear receptor interacting protein 1	L1
1423132_a_at	NSA2	NSA2 ribosome biogenesis homolog (S. cerevisiae)	
1422456_at	NSF	N-ethylmaleimide-sensitive factor	L1
1447705_at	NSL1	NSL1, MIND kinetochore complex component, homolog (S. cerevisiae)	
1416412_at	NSMAF	neutral sphingomyelinase (N-SMase) activation associated factor	L1
1428213_at	NSMCE4A	non-SMC element 4 homolog A (S. cerevisiae)	L1
1418905_at	NUBP1	nucleotide binding protein 1	L1
1416903_at	NUCB1	nucleobindin 1	L1
1444952_a_at	NUCKS1	nuclear casein kinase and cyclin-dependent kinase substrate 1	L1
1442799_x_at	NUDCD3	NudC domain containing 3	L1
1437213_at	NUDT21	nudix (nucleoside diphosphate linked moiety X)-type motif 21	L1
1455966_s_at	NUDT21	nudix (nucleoside diphosphate linked moiety X)-type motif 21	L1
1460358_s_at	NUDT22	nudix (nucleoside diphosphate linked moiety X)-type motif 22	L1
1423787_at	NUP133	nucleoporin 133kDa	L1
1426447_at	NUP35	nucleoporin 35kDa	
1416073_a_at	NUP85	nucleoporin 85kDa	L1
1434220_at	Nup98	nucleoporin 98	L1
1416452_at	OAT	ornithine aminotransferase	L1
1426764_at	OAZ2	ornithine decarboxylase antizyme 2	L1
1438396_at	OCRL	oculocerebrorenal syndrome of Lowe	
1451888_a_at	ODZ4	odz, odd Oz/ten-m homolog 4 (Drosophila)	
1434530_at	ODZ4	odz, odd Oz/ten-m homolog 4 (Drosophila)	L1
1455993_at	ODZ4	odz, odd Oz/ten-m homolog 4 (Drosophila)	L1
1426562_a_at	OLFM1	olfactomedin 1	L1
1432098_a_at	Olfr701	olfactory receptor 701	

1419534_at	OLR1	oxidized low density lipoprotein (lectin-like) receptor 1	L1
1435679_at	OPTN	optineurin	L1
1418227_at	ORC2	origin recognition complex, subunit 2	L1
1453804_a_at	ORC4	origin recognition complex, subunit 4	L1
1428355_at	OSBP2	oxysterol binding protein 2	L1
1434223_at	OSCP1	organic solute carrier partner 1	
1428557_a_at	OSGEPL1	O-sialoglycoprotein endopeptidase-like 1	L1
1426000_at	OXTR	oxytocin receptor	
1419853_a_at	P2RX7	purinergic receptor P2X, ligand-gated ion channel, 7	L1
1426519_at	P4HA1	prolyl 4-hydroxylase, alpha polypeptide I	
1437465_a_at	P4HB	prolyl 4-hydroxylase, beta polypeptide	L1
1435372_a_at	PA2G4	proliferation-associated 2G4, 38kDa	L1
1435762_at	PACS1	phosphofurin acidic cluster sorting protein 1	L1
1434255_at	PACS2	phosphofurin acidic cluster sorting protein 2	L1
1456403_at	PAG1	phosphoprotein associated with glycosphingolipid microdomains 1	
1430875_a_at	PAK1IP1	PAK1 interacting protein 1	
1427228_at	PALLD	palladin, cytoskeletal associated protein	
1423967_at	PALM	paralemmin	L1
1435004_at	PANK4	pantothenate kinase 4	L1
1415890_at	PAPSS1	3'-phosphoadenosine 5'-phosphosulfate synthase 1	L1
1425422_a_at	PARN	poly(A)-specific ribonuclease	L1
1451969_s_at	PARP3	poly (ADP-ribose) polymerase family, member 3	L1
1416818_at	PARVA	parvin, alpha	L1
1423302_a_at	PAXIP1	PAX interacting (with transcription-activation domain) protein 1	L1

1423303_at	PAXIP1	PAX interacting (with transcription-activation domain) protein 1	L1
1436920_at	PCDH17	protocadherin 17	L1
1437360_at	PCDH19	protocadherin 19	L1
1448933_at	PCDHB16	protocadherin beta 16	L1
1419048_at	PCNX	pecanex homolog (Drosophila)	L1
1434020_at	PDAP1	PDGFA associated protein 1	L1
1423534_at	PDCD2	programmed cell death 2	L1
1426844_a_at	PDCD2L	programmed cell death 2-like	L1
1426845_at	PDCD2L	programmed cell death 2-like	L1
1433761_at	PDE4DIP	phosphodiesterase 4D interacting protein	L1
1425279_at	PDIK1L	PDLIM1 interacting kinase 1 like	L1
1441285_at	PDP2	pyruvate dehyrogenase phosphatase catalytic subunit 2	L1
1431893_a_at	PDSS1	prenyl (decaprenyl) diphosphate synthase, subunit 1	L1
1452705_at	PDXDC1	pyridoxal-dependent decarboxylase domain containing 1	L1
1421287_a_at	PECAM1	platelet/endothelial cell adhesion molecule 1	L1
1453207_at	PET100	PET100 homolog (S. cerevisiae)	
1456646_at	PEX10	peroxisomal biogenesis factor 10	L1
1417442_a_at	PEX3	peroxisomal biogenesis factor 3	L1
1454044_a_at	PEX3	peroxisomal biogenesis factor 3	L1
1422091_at	PFKFB2	6-phosphofructo-2-kinase/fructose-2,6-biphosphatase 2	L1
1416432_at	PFKFB3	6-phosphofructo-2-kinase/fructose-2,6-biphosphatase 3	L1
1426554_a_at	PGAM1	phosphoglycerate mutase 1 (brain)	L1
1438774_s_at	PGM2L1	phosphoglucomutase 2-like 1	
1456606_a_at	PHACTR1	phosphatase and actin regulator 1	L1
1423212_at	PHC1	polyhomeotic homolog 1 (Drosophila)	L1
1452179_at	PHF17	PHD finger protein 17	L1
1421069_at	PHF2	PHD finger protein 2	

1457264_at	PHF20L1	PHD finger protein 20-like 1	
1417837_at	PHLDA2	pleckstrin homology-like domain, family A, member 2	
1437115_at	PHRF1	PHD and ring finger domains 1	L1
1451115_at	PIAS3	protein inhibitor of activated STAT, 3	L1
1428488_at	PIGK	phosphatidylinositol glycan anchor biosynthesis, class K	L1
1433429_at	PIGS	phosphatidylinositol glycan anchor biosynthesis, class S	L1
1437956_at	PIK3R6	phosphoinositide-3-kinase, regulatory subunit 6	L1
1416387_at	PIP4K2C	phosphatidylinositol-5-phosphate 4-kinase, type II, gamma	L1
1426449_a_at	PJA1	praja ring finger 1, E3 ubiquitin protein ligase	L1
1420259_at	PKP2	plakophilin 2	L1
1450533_a_at	PLAGL1	pleiomorphic adenoma gene-like 1	
1448786_at	PLBD1	phospholipase B domain containing 1	L1
1416675_s_at	PLCD1	phospholipase C, delta 1	L1
1448432_at	PLCD1	phospholipase C, delta 1	L1
1416013_at	PLD3	phospholipase D family, member 3	L1
1460341_at	PLEKHB2	pleckstrin homology domain containing, family B (evectins) member 2	L1
1418595_at	PLIN4	perilipin 4	L1
1415901_at	PLOD3	procollagen-lysine, 2-oxoglutarate 5-dioxygenase 3	L1
1418912_at	PLXDC2	plexin domain containing 2	L1
1424661_at	PMPCA	peptidase (mitochondrial processing) alpha	
1429709_at	PMPCB	peptidase (mitochondrial processing) beta	L1
1453185_at	PNISR	PNN-interacting serine/arginine-rich protein	
1429537_at	PNISR	PNN-interacting serine/arginine-rich protein	L1

1423325_at	PNN	pinin, desmosome associated protein	L1
1450735_at	PNO1	partner of NOB1 homolog (S. cerevisiae)	L1
1444288_at	PNPT1	polyribonucleotide nucleotidyltransferase 1	L1
1419265_at	POC5	POC5 centriolar protein homolog (Chlamydomonas)	L1
1416573_at	POFUT2	protein O-fucosyltransferase 2	L1
1448384_at	POFUT2	protein O-fucosyltransferase 2	L1
1449352_at	POLL	polymerase (DNA directed), lambda	L1
1456066_a_at	POLR1A	polymerase (RNA) I polypeptide A, 194kDa	L1
1415754_at	POLR2F	polymerase (RNA) II (DNA directed) polypeptide F	
1453256_at	POLR3C	polymerase (RNA) III (DNA directed) polypeptide C (62kD)	L1
1450686_at	PON2	paraoxonase 2	L1
1451210_at	PPAP2C	phosphatidic acid phosphatase type 2C	L1
1428154_s_at	PPAPDC1B	phosphatidic acid phosphatase type 2 domain containing 1B	L1
1417801_a_at	PPFIBP2	PTPRF interacting protein, binding protein 2 (liprin beta 2)	L1
1416498_at	PPIC	peptidylprolyl isomerase C (cyclophilin C)	L1
1434475_at	PPIG	peptidylprolyl isomerase G (cyclophilin G)	
1460584_at	PPIP5K1	diphosphoinositol pentakisphosphate kinase 1	L1
1453171_s_at	PPM1A	protein phosphatase, Mg2+/Mn2+ dependent, 1A	L1
1454934_at	PPM1F	protein phosphatase, Mg2+/Mn2+ dependent, 1F	L1
1435699_at	PPM1L	protein phosphatase, Mg2+/Mn2+ dependent, 1L	
1434786_at	PPP1R12B	protein phosphatase 1, regulatory subunit 12B	L1
1454848_at	PPP1R12C	protein phosphatase 1, regulatory subunit 12C	L1

1436716_at	PPP1R14B	protein phosphatase 1, regulatory (inhibitor) subunit 14B	
1436366_at	PPP1R15B	protein phosphatase 1, regulatory subunit 15B	
1426798_a_at	PPP1R15B	protein phosphatase 1, regulatory subunit 15B	L1
1417341_a_at	Ppp1r2	protein phosphatase 1, regulatory (inhibitor) subunit 2	L1
1442024_at	PPP1R3E	protein phosphatase 1, regulatory subunit 3E	L1
1437730_at	PPP2R2A	protein phosphatase 2, regulatory subunit B, alpha	L1
1428463_a_at	PPP2R5E	protein phosphatase 2, regulatory subunit B', epsilon isoform	L1
1424115_at	PPP5C	protein phosphatase 5, catalytic subunit	L1
1424116_x_at	PPP5C	protein phosphatase 5, catalytic subunit	L1
1451242_a_at	PPP5C	protein phosphatase 5, catalytic subunit	L1
1422468_at	PPT1	palmitoyl-protein thioesterase 1	L1
1439160_at	PRAMEF7/PRAMEF8	PRAME family member 8	
1452208_at	PRDM4	PR domain containing 4	L1
1436962_at	PRDM6	PR domain containing 6	
1416167_at	PRDX4	peroxiredoxin 4	L1
1455667_at	PREB	prolactin regulatory element binding	L1
1424119_at	PRKAB1	protein kinase, AMP-activated, beta 1 non-catalytic subunit	L1
1428230_at	PRKD3	protein kinase D3	L1
1449876_at	PRKG1	protein kinase, cGMP-dependent, type I	
1448923_at	PRKRA	protein kinase, interferon-inducible double stranded RNA dependent activator	L1
1427439_s_at	PRMT5	protein arginine methyltransferase 5	
1420664_s_at	PROCR	protein C receptor, endothelial	L1
1455524_at	PROSER1	proline and serine rich 1	L1

1460633_at	PRPF19	PRP19/PSO4 pre-mRNA processing factor 19 homolog (S. cerevisiae)	L1
1422453_at	PRPF8	PRP8 pre-mRNA processing factor 8 homolog (S. cerevisiae)	L1
1416052_at	PRPS1	phosphoribosyl pyrophosphate synthetase 1	L1
1460349_at	PRR14	proline rich 14	L1
1432613_at	PRR15	proline rich 15	L1
1424058_at	PRRC1	proline-rich coiled-coil 1	L1
1429433_at	PRRC2C	proline-rich coiled-coil 2C	
1431484_at	PRRG1	proline rich Gla (G-carboxyglutamic acid) 1	L1
1441707_at	PSMA3	proteasome (prosome, macropain) subunit, alpha type, 3	
1416291_at	PSMC4	proteasome (prosome, macropain) 26S subunit, ATPase, 4	L1
1423697_at	PSMD6	proteasome (prosome, macropain) 26S subunit, non-ATPase, 6	L1
1415673_at	PSPH	phosphoserine phosphatase	L1
1428853_at	PTCH1	patched 1	
1422655_at	PTCH2	patched 2	
1460010_a_at	PTDSS2	phosphatidylserine synthase 2	L1
1449449_at	PTGES	prostaglandin E synthase	L1
1449450_at	PTGES	prostaglandin E synthase	L1
1417092_at	PTH1R	parathyroid hormone 1 receptor	L1
1434653_at	PTK2B	PTK2B protein tyrosine kinase 2 beta	L1
1418181_at	PTP4A3	protein tyrosine phosphatase type IVA, member 3	L1
1433823_at	PTPDC1	protein tyrosine phosphatase domain containing 1	L1
1427699_a_at	PTPN11	protein tyrosine phosphatase, non-receptor type 11	
1417140_a_at	PTPN2	protein tyrosine phosphatase, non-receptor type 2	L1
1427486_at	PTPRB	protein tyrosine phosphatase, receptor type, B	L1

1420841_at	PTPRF	protein tyrosine phosphatase, receptor type, F	
1420843_at	PTPRF	protein tyrosine phosphatase, receptor type, F	L1
1434360_s_at	PTPRG	protein tyrosine phosphatase, receptor type, G	L1
1423117_at	PUM1	pumilio homolog 1 (Drosophila)	L1
1428800_a_at	PUS7L	pseudouridylate synthase 7 homolog (S. cerevisiae)-like	
1448673_at	PVRL3	poliovirus receptor-related 3	L1
1451253_at	PXK	PX domain containing serine/threonine kinase	L1
1417741_at	PYGL	phosphorylase, glycogen, liver	L1
1423712_a_at	QARS	glutaminyl-tRNA synthetase	L1
1449518_at	QPCTL	glutaminyl-peptide cyclotransferase-like	L1
1452736_at	R3HCC1	R3H domain and coiled-coil containing 1	
1438905_x_at	R3HDM4	R3H domain containing 4	L1
1438022_at	RAB11FIP3	RAB11 family interacting protein 3 (class II)	L1
1419946_s_at	RAB2A	RAB2A, member RAS oncogene family	
1433922_at	RAB35	RAB35, member RAS oncogene family	L1
1417700_at	RAB38	RAB38, member RAS oncogene family	
1418341_at	RAB4A	RAB4A, member RAS oncogene family	L1
1425602_a_at	RABEP2	rabaptin, RAB GTPase binding effector protein 2	L1
1456822_at	RAD23B	RAD23 homolog B (S. cerevisiae)	
1416602_a_at	RAD52	RAD52 homolog (S. cerevisiae)	L1
1417248_at	RALBP1	ralA binding protein 1	L1
1443877_a_at	RAPGEF6	Rap guanine nucleotide exchange factor (GEF) 6	L1
1428538_s_at	RARRES2	retinoic acid receptor responder (tazarotene induced) 2	
1423619_at	RASD1	RAS, dexamethasone-induced 1	L1
1438713_at	RASSF8	Ras association (RalGDS/AF-6) domain family (N-terminal) member 8	

1426487_a_at	RBBP6	retinoblastoma binding protein 6	L1
1416174_at	RBBP9	retinoblastoma binding protein 9	L1
1425166_at	RBL1	retinoblastoma-like 1 (p107)	
1418146_a_at	RBL2	retinoblastoma-like 2 (p130)	L1
1425981_a_at	RBL2	retinoblastoma-like 2 (p130)	L1
1423741_at	RBM10	RNA binding motif protein 10	L1
1425522_at	RBM25	RNA binding motif protein 25	L1
1455430_at	RBM33	RNA binding motif protein 33	L1
1426671_a_at	RBM39	RNA binding motif protein 39	L1
1418703_at	RBMS1	RNA binding motif, single stranded interacting protein 1	L1
1429359_s_at	RBPMS	RNA binding protein with multiple splicing	
1444765_at	RBPMS	RNA binding protein with multiple splicing	
1425652_s_at	RBPMS	RNA binding protein with multiple splicing	L1
1434027_at	RCAN3	RCAN family member 3	
1416390_at	RCBTB2	regulator of chromosome condensation (RCC1) and BTB (POZ) domain containing protein 2	
1416730_at	RCL1	RNA terminal phosphate cyclase-like 1	L1
1422449_s_at	RCN2	reticulocalbin 2, EF-hand calcium binding domain	L1
1434336_s_at	RCOR1	REST corepressor 1	L1
1421235_s_at	RECQL5	RecQ protein-like 5	L1
1433509_s_at	REEP1	receptor accessory protein 1	
1430128_a_at	REEP6	receptor accessory protein 6	L1
1417856_at	RELB	v-rel reticuloendotheliosis viral oncogene homolog B	L1
1416216_at	REPS1	RALBP1 associated Eps domain containing 1	L1
1454670_at	RERE	arginine-glutamic acid dipeptide (RE) repeats	L1
1424321_at	RFC4	replication factor C (activator 1) 4, 37kDa	L1
1434826_at	RFESD	Rieske (Fe-S) domain containing	L1
1418003_at	RGCC	regulator of cell cycle	L1
1417466_at	RGS5	regulator of G-protein signaling 5	L1
1438163_x_at	RHBDD2	rhomboid domain containing 2	L1

1424819_a_at	RIC8A	resistance to inhibitors of cholinesterase 8 homolog A (C. elegans)	L1
1450449_a_at	RILPL1	Rab interacting lysosomal protein-like 1	L1
1422650_a_at	RIOK3	RIO kinase 3 (yeast)	L1
1460670_at	RIOK3	RIO kinase 3 (yeast)	L1
1439273_at	RIPK1	receptor (TNFRSF)-interacting serine-threonine kinase 1	L1
1450173_at	RIPK2	receptor-interacting serine-threonine kinase 2	L1
1428710_at	RIT1	Ras-like without CAAX 1	L1
1423102_a_at	RNF10	ring finger protein 10	L1
1428415_at	RNF113A2	ring finger protein 113A2	
1419368_a_at	RNF138	ring finger protein 138, E3 ubiquitin protein ligase	L1
1429425_at	RNF139	ring finger protein 139	
1429321_at	RNF149	ring finger protein 149	L1
1455586_at	RNF168	ring finger protein 168, E3 ubiquitin protein ligase	
1432478_a_at	RNF19B	ring finger protein 19B	L1
1427273_at	RNF214	ring finger protein 214	L1
1436305_at	RNF217	ring finger protein 217	L1
1439447_x_at	RPL37A	ribosomal protein L37a	L1
1451077_at	RPL5	ribosomal protein L5	L1
1418896_a_at	RPN2	ribophorin II	L1
1434563_at	RPS6KC1	ribosomal protein S6 kinase, 52kDa, polypeptide 1	L1
1434619_at	RPTOR	regulatory associated protein of MTOR, complex 1	L1
1422562_at	RRAD	Ras-related associated with diabetes	
1434741_at	RREB1	ras responsive element binding protein 1	
1436058_at	RSAD2	radical S-adenosyl methionine domain containing 2	
1432579_at	RSPH3	radial spoke 3 homolog (Chlamydomonas)	L1
1451640_a_at	RSRC2	arginine/serine-rich coiled-coil 2	L1
1424095_at	RTCA	RNA 3'-terminal phosphate cyclase	
1439573_at	RTN4RL2	reticulon 4 receptor-like 2	

1426791_at	RUSC2	RUN and SH3 domain containing 2	L1
1416585_at	RUVBL1	RuvB-like 1 (E. coli)	L1
1454773_at	RXRA	retinoid X receptor, alpha	L1
1437173_at	S1PR3	sphingosine-1-phosphate receptor 3	L1
1438900_at	SACM1L	SAC1 suppressor of actin mutations 1-like (yeast)	
1440185_x_at	SAE1	SUMO1 activating enzyme subunit 1	L1
1415718_at	SAP30L	SAP30-like	L1
1448426_at	SARDH	sarcosine dehydrogenase	L1
1416007_at	SATB1	SATB homeobox 1	L1
1427132_at	SBF2	SET binding factor 2	L1
1443721_x_at	SBNO2	strawberry notch homolog 2 (Drosophila)	L1
1458308_at	SBNO2	strawberry notch homolog 2 (Drosophila)	L1
1459999_at	SCAF4	SR-related CTD-associated factor 4	L1
1438419_at	SCAF8	SR-related CTD-associated factor 8	
1433520_at	SCAP	SREBF chaperone	L1
1434740_at	SCARF2	scavenger receptor class F, member 2	L1
1426510_at	SCCPDH	saccharopine dehydrogenase (putative)	L1
1415824_at	SCD2	stearoyl-Coenzyme A desaturase 2	
1428335_a_at	SCFD1	sec1 family domain containing 1	L1
1441192_at	SCLY	selenocysteine lyase	L1
1436804_s_at	SCYL1	SCY1-like 1 (S. cerevisiae)	L1
1451051_a_at	SCYL1	SCY1-like 1 (S. cerevisiae)	L1
1428918_at	SCYL3	SCY1-like 3 (S. cerevisiae)	
1415943_at	SDC1	syndecan 1	L1
1443832_s_at	SDPR	serum deprivation response	L1
1416241_at	SEC13	SEC13 homolog (S. cerevisiae)	L1
1416059_at	SEC23B	Sec23 homolog B (S. cerevisiae)	L1
1438121_at	SEC24A	SEC24 family, member A (S. cerevisiae)	L1
1453014_a_at	SEC31A	SEC31 homolog A (S. cerevisiae)	L1
1424926_at	SEC63	SEC63 homolog (S. cerevisiae)	

1456120_at	SECISBP2L	SECIS binding protein 2-like	
1425188_s_at	SEL1L	sel-1 suppressor of lin-12-like (C. elegans)	L1
1430692_a_at	SEL1L	sel-1 suppressor of lin-12-like (C. elegans)	L1
1420696_at	SEMA3C	sema domain, immunoglobulin domain (Ig), short basic domain, secreted, (semaphorin) 3C	L1
1453055_at	SEMA6D	sema domain, transmembrane domain (TM), and cytoplasmic domain, (semaphorin) 6D	L1
1454877_at	SERTAD4	SERTA domain containing 4	
1428555_at	SETD2	SET domain containing 2	L1
1423750_a_at	SF1	splicing factor 1	L1
1456352_a_at	SF3B2	splicing factor 3b, subunit 2, 145kDa	L1
1439058_at	SFPQ	splicing factor proline/glutamine-rich	L1
1438620_x_at	SFRP1	secreted frizzled-related protein 1	
1424003_at	SGK196	protein kinase-like protein SgK196	L1
1415892_at	SGPL1	sphingosine-1-phosphate lyase 1	L1
1437977_at	SGTB	small glutamine-rich tetratricopeptide repeat (TPR)-containing, beta	
1433455_at	SH2B3	SH2B adaptor protein 3	L1
1415886_at	SH2D3C	SH2 domain containing 3C	L1
1421922_at	SH3BP5	SH3-domain binding protein 5 (BTK-associated)	L1
1416338_at	SH3GL1	SH3-domain GRB2-like 1	L1
1432269_a_at	SH3KBP1	SH3-domain kinase binding protein 1	L1
1448171_at	SIAH2	siah E3 ubiquitin protein ligase 2	L1
1438442_at	SIKE1	suppressor of IKBKE 1	L1
1428916_s_at	SIRT5	sirtuin 5	L1
1422584_at	SKIV2L	superkiller viralicidic activity 2-like (S. cerevisiae)	L1
1460168_at	SLBP	stem-loop binding protein	L1
1458918_at	SLC12A8	solute carrier family 12 (potassium/chloride transporters), member 8	L1

1427722_at	SLC15A5	solute carrier family 15, member 5	
1440569_at	SLC16A10	solute carrier family 16, member 10 (aromatic amino acid transporter)	L1
1418446_at	SLC16A2	solute carrier family 16, member 2 (thyroid hormone transporter)	
1417884_at	SLC16A6	solute carrier family 16, member 6 (monocarboxylic acid transporter 7)	L1
1457302_at	SLC20A2	solute carrier family 20 (phosphate transporter), member 2	
1424308_at	SLC24A3	solute carrier family 24 (sodium/potassium/calcium exchanger), member 3	L1
1428440_at	SLC25A12	solute carrier family 25 (aspartate/glutamate carrier), member 12	L1
1451489_at	SLC25A35	solute carrier family 25, member 35	
1430542_a_at	SLC25A5	solute carrier family 25 (mitochondrial carrier; adenine nucleotide translocator), member 5	L1
1416316_at	SLC27A2	solute carrier family 27 (fatty acid transporter), member 2	
1450639_at	SLC28A2	solute carrier family 28 (sodium-coupled nucleoside transporter), member 2	L1
1426599_a_at	SLC2A1	solute carrier family 2 (facilitated glucose transporter), member 1	
1426600_at	SLC2A1	solute carrier family 2 (facilitated glucose transporter), member 1	L1
1419434_at	SLC2A10	solute carrier family 2 (facilitated glucose transporter), member 10	
1416639_at	SLC2A5	solute carrier family 2 (facilitated glucose/fructose transporter), member 5	

1450697_at	SLC30A7	solute carrier family 30 (zinc transporter), member 7	
1448769_at	SLC35B1	solute carrier family 35, member B1	L1
1416548_at	SLC35B4	solute carrier family 35, member B4	L1
1416549_at	SLC35B4	solute carrier family 35, member B4	L1
1427294_a_at	SLC38A10	solute carrier family 38, member 10	L1
1427295_at	SLC38A10	solute carrier family 38, member 10	L1
1417881_at	SLC39A3	solute carrier family 39 (zinc transporter), member 3	L1
1417061_at	SLC40A1	solute carrier family 40 (iron-regulated transporter), member 1	
1425439_a_at	SLC41A3	solute carrier family 41, member 3	L1
1423865_at	SLC44A1	solute carrier family 44, member 1	L1
1433645_at	SLC44A1	solute carrier family 44, member 1	L1
1450409_a_at	SLC48A1	solute carrier family 48 (heme transporter), member 1	L1
1450410_a_at	SLC48A1	solute carrier family 48 (heme transporter), member 1	L1
1426432_a_at	SLC4A4	solute carrier family 4, sodium bicarbonate cotransporter, member 4	
1460541_at	SLC7A6	solute carrier family 7 (amino acid transporter light chain, y+L system), member 6	L1
1440962_at	SLC8A3	solute carrier family 8 (sodium/calcium exchanger), member 3	L1
1433933_s_at	SLCO2B1	solute carrier organic anion transporter family, member 2B1	L1
1451655_at	SLFN13	schlafen family member 13	L1
1456288_at	SLFN5	schlafen family member 5	
1419092_a_at	SLK	STE20-like kinase	
1437707_at	SLMO1	slowmo homolog 1 (Drosophila)	

1456683_at	SLTM	SAFB-like, transcription modulator	L1
1459843_s_at	SMAD1	SMAD family member 1	
1420634_a_at	SMAD2	SMAD family member 2	L1
1422485_at	SMAD4	SMAD family member 4	L1
1426804_at	SMARCA4	SWI/SNF related, matrix associated, actin dependent regulator of chromatin, subfamily a, member 4	L1
1416045_a_at	SMARCB1	SWI/SNF related, matrix associated, actin dependent regulator of chromatin, subfamily b, member 1	L1
1418467_at	SMARCD3	SWI/SNF related, matrix associated, actin dependent regulator of chromatin, subfamily d, member 3	L1
1417832_at	SMC1A	structural maintenance of chromosomes 1A	L1
1429658_a_at	SMC2	structural maintenance of chromosomes 2	L1
1417736_at	SMC6	structural maintenance of chromosomes 6	
1429017_at	SMCR8	Smith-Magenis syndrome chromosome region, candidate 8	L1
1457262_at	SMG1	smg-1 homolog, phosphatidylinositol 3-kinase-related kinase (C. elegans)	
1427048_at	SMO	smoothened, frizzled family receptor	
1427049_s_at	SMO	smoothened, frizzled family receptor	
1448321_at	SMOC1	SPARC related modular calcium binding 1	L1
1415935_at	SMOC2	SPARC related modular calcium binding 2	L1
1448621_a_at	SMPD1	sphingomyelin phosphodiesterase 1, acid lysosomal	L1
1428395_at	SMURF1	SMAD specific E3 ubiquitin protein ligase 1	L1

1424760_a_at	SMYD2	SET and MYND domain containing 2	L1
1423745_at	SNAP47	synaptosomal-associated protein, 47kDa	L1
1417788_at	SNCG	synuclein, gamma (breast cancer-specific protein 1)	
1447993_a_at	Snhg10	small nucleolar RNA host gene (non-protein coding) 10	L1
1452789_at	SNN	stannin	L1
1430041_at	SNRNP35	small nuclear ribonucleoprotein 35kDa (U11/U12)	
1452680_at	SNRPD2	small nuclear ribonucleoprotein D2 polypeptide 16.5kDa	
1420371_at	SNTB2	syntrophin, beta 2 (dystrophin-associated protein A1, 59kDa, basic component 2)	
1424276_at	SNX16	sorting nexin 16	L1
1433614_at	SNX27	sorting nexin family member 27	L1
1425148_a_at	SNX6	sorting nexin 6	L1
1423076_at	SNX9	sorting nexin 9	L1
1417695_a_at	SOAT1	sterol O-acyltransferase 1	L1
1416576_at	SOCS3	suppressor of cytokine signaling 3	L1
1455634_at	SON	SON DNA binding protein	
1419358_at	SORCS2	sortilin-related VPS10 domain containing receptor 2	
1437676_at	SPAG9	sperm associated antigen 9	L1
1416114_at	SPARCL1	SPARC-like 1 (hevin)	L1
1428300_at	SPECC1L	sperm antigen with calponin homology and coiled-coil domains 1-like	L1
1451036_at	SPG21	spastic paraplegia 21 (autosomal recessive, Mast syndrome)	L1
1454962_at	SPIRE1	spire homolog 1 (Drosophila)	L1
1420150_at	SPSB1	splA/ryanodine receptor domain and SOCS box containing 1	L1
1428472_at	SPSB1	splA/ryanodine receptor domain and SOCS box containing 1	L1
1423574_s_at	SRD5A3	steroid 5 alpha-reductase 3	L1
1456691_s_at	SRD5A3	steroid 5 alpha-reductase 3	L1

1418256_at	SRF	serum response factor (c-fos serum response element-binding transcription factor)	L1
1456355_s_at	SRRD	SRR1 domain containing	
1452439_s_at	SRSF2	serine/arginine-rich splicing factor 2	L1
1447898_s_at	SRSF6	serine/arginine-rich splicing factor 6	L1
1419942_at	SRXN1	sulfiredoxin 1	
1425351_at	SRXN1	sulfiredoxin 1	L1
1426875_s_at	SRXN1	sulfiredoxin 1	L1
1451680_at	SRXN1	sulfiredoxin 1	L1
1419360_a_at	SS18	synovial sarcoma translocation, chromosome 18	L1
1438253_at	SSH1	slingshot homolog 1 (Drosophila)	L1
1421915_a_at	ST3GAL3	ST3 beta-galactoside alpha-2,3-sialyltransferase 3	L1
1450406_a_at	ST3GAL3	ST3 beta-galactoside alpha-2,3-sialyltransferase 3	L1
1431962_a_at	STAMBP	STAM binding protein	L1
1452604_at	STARD13	StAR-related lipid transfer (START) domain containing 13	L1
1421469_a_at	STAT5A	signal transducer and activator of transcription 5A	L1
1422102_a_at	STAT5B	signal transducer and activator of transcription 5B	L1
1448320_at	STIM1	stromal interaction molecule 1	L1
1426343_at	STT3B	STT3, subunit of the oligosaccharyltransferase complex, homolog B (S. cerevisiae)	L1
1416580_a_at	STUB1	STIP1 homology and U-box containing protein 1, E3 ubiquitin protein ligase	L1
1423038_at	STX6	syntaxin 6	L1
1431646_a_at	STX6	syntaxin 6	L1
1427441_a_at	SUCLG2	succinate-CoA ligase, GDP-forming, beta subunit	L1
1450024_at	SUFU	suppressor of fused homolog (Drosophila)	
1447602_x_at	SULF2	sulfatase 2	

1415782_at	SUMO2	SMT3 suppressor of mif two 3 homolog 2 (yeast)	L1
1426666_a_at	SUN1	Sad1 and UNC84 domain containing 1	L1
1416213_x_at	SURF4	surfeit 4	L1
1436797_a_at	SURF4	surfeit 4	L1
1428654_at	SWT1	SWT1 RNA endoribonuclease homolog (S. cerevisiae)	L1
1460230_at	SYN2	synapsin II	
1427982_s_at	SYNE2	spectrin repeat containing, nuclear envelope 2	
1438585_at	SYNE2	spectrin repeat containing, nuclear envelope 2	
1419289_a_at	SYNGR1	synaptogyrin 1	
1436334_at	SYNJ1	synaptojanin 1	L1
1452344_at	SYNJ2	synaptojanin 2	
1441927_at	SYT7	synaptotagmin VII	L1
1428070_at	SYVN1	synovial apoptosis inhibitor 1, synoviolin	L1
1443609_s_at	SYVN1	synovial apoptosis inhibitor 1, synoviolin	L1
1425745_a_at	TACC2	transforming, acidic coiled-coil containing protein 2	L1
1417467_a_at	TADA3	transcriptional adaptor 3	L1
1447417_at	TAF1	TAF1 RNA polymerase II, TATA box binding protein (TBP)-associated factor, 250kDa	
1431345_a_at	TAF1B	TATA box binding protein (TBP)-associated factor, RNA polymerase I, B, 63kDa	L1
1452438_s_at	TAF4	TAF4 RNA polymerase II, TATA box binding protein (TBP)-associated factor, 135kDa	L1
1422778_at	TAF9	TAF9 RNA polymerase II, TATA box binding protein (TBP)-associated factor, 32kDa	L1
1452714_at	TANC1	tetratricopeptide repeat, ankyrin repeat and coiled-coil containing 1	L1
1416062_at	TBC1D15	TBC1 domain family, member 15	
1423659_a_at	TBC1D17	TBC1 domain family, member 17	L1
1426899_at	TBC1D23	TBC1 domain family, member 23	L1

1428508_at	TBC1D2B	TBC1 domain family, member 2B	L1
1428598_at	TBC1D7	TBC1 domain family, member 7	L1
1428596_at	TBC1D9B	TBC1 domain family, member 9B (with GRAM domain)	L1
1428282_at	TBCE	tubulin folding cofactor E	L1
1445571_at	TBK1	TANK-binding kinase 1	L1
1429974_at	TBX18	T-box 18	L1
1422545_at	TBX2	T-box 2	L1
1424531_a_at	TCEA3	transcription elongation factor A (SII), 3	L1
1434117_at	TCEB3	transcription elongation factor B (SIII), polypeptide 3 (110kDa, elongin A)	L1
1417447_at	TCF21	transcription factor 21	L1
1437204_a_at	TCF25	transcription factor 25 (basic helix-loop-helix)	L1
1437205_at	TCF25	transcription factor 25 (basic helix-loop-helix)	L1
1424068_at	TCTA	T-cell leukemia translocation altered	L1
1452264_at	TENC1	tensin like C1 domain containing phosphatase (tensin 2)	L1
1455651_at	TERF2	telomeric repeat binding factor 2	L1
1426049_a_at	TERF2IP	telomeric repeat binding factor 2, interacting protein	L1
1450662_at	TESK1	testis-specific kinase 1	L1
1448224_at	TFAM	transcription factor A, mitochondrial	L1
1448977_at	TFAP2C	transcription factor AP-2 gamma (activating enhancer binding protein 2 gamma)	L1
1438053_at	TFG	TRK-fused gene	L1
1448976_at	TFIP11	tuftelin interacting protein 11	L1
1459994_x_at	TFR2	transferrin receptor 2	
1415871_at	TGFBI	transforming growth factor, beta-induced, 68kDa	L1
1420895_at	TGFBR1	transforming growth factor, beta receptor 1	L1
1421355_at	TGM3	transglutaminase 3 (E polypeptide, protein-glutamine-gamma-glutamyltransferase)	L1
1416428_at	THAP11	THAP domain containing 11	L1

1424052_at	THAP4	THAP domain containing 4	L1
1425244_a_at	THEG	theg spermatid protein	L1
1426997_at	THRA	thyroid hormone receptor, alpha	
1427408_a_at	THRAP3	thyroid hormone receptor associated protein 3	L1
1438769_a_at	THYN1	thymocyte nuclear protein 1	L1
1419089_at	TIMP3	TIMP metallopeptidase inhibitor 3	L1
1451587_a_at	TIPRL	TIP41, TOR signaling pathway regulator-like (S. cerevisiae)	L1
1434033_at	TLE1	transducin-like enhancer of split 1 (E(sp1) homolog, Drosophila)	
1448402_at	TLN1	talin 1	
1435700_at	TLN2	talin 2	
1426254_at	TM2D1	TM2 domain containing 1	L1
1439454_x_at	TM2D2	TM2 domain containing 2	L1
1451134_a_at	TM2D2	TM2 domain containing 2	L1
1439925_at	TM4SF1	transmembrane 4 L six family member 1	L1
1423204_at	TM9SF4	transmembrane 9 superfamily protein member 4	L1
1423205_at	TM9SF4	transmembrane 9 superfamily protein member 4	L1
1426711_at	TMCO3	transmembrane and coiled-coil domains 3	L1
1435883_at	TMCO7	transmembrane and coiled-coil domains 7	L1
1426649_at	TMEFF1	transmembrane protein with EGF-like and two follistatin-like domains 1	L1
1451315_at	TMEM101	transmembrane protein 101	
1452290_at	TMEM106B	transmembrane protein 106B	L1
1427324_at	TMEM120B	transmembrane protein 120B	L1
1415715_at	TMEM129	transmembrane protein 129	L1
1421993_a_at	TMEM134	transmembrane protein 134	L1
1433017_at	TMEM159	transmembrane protein 159	L1
1415741_at	TMEM165	transmembrane protein 165	
1459997_s_at	TMEM17	transmembrane protein 17	L1
1452134_at	TMEM175	transmembrane protein 175	L1
1451341_s_at	TMEM189	transmembrane protein 189	L1
1423707_at	TMEM50B	transmembrane protein 50B	L1
1429337_at	TMEM87B	transmembrane protein 87B	L1

1417874_at	TMEM9B	TMEM9 domain family, member B	L1
1438556_a_at	TMOD3	tropomodulin 3 (ubiquitous)	L1
1448147_at	TNFRSF19	tumor necrosis factor receptor superfamily, member 19	L1
1442590_at	TNFRSF22/TNFRSF23	tumor necrosis factor receptor superfamily, member 22	L1
1448298_at	TNK2	tyrosine kinase, non-receptor, 2	L1
1453124_at	TNPO3	transportin 3	L1
1431776_at	TOB2	transducer of ERBB2, 2	L1
1426084_a_at	TOR1AIP1	torsin A interacting protein 1	L1
1423783_at	TOR2A	torsin family 2, member A	L1
1426538_a_at	TP53	tumor protein p53	
1433937_at	TP53BP2	tumor protein p53 binding protein, 2	L1
1428189_at	TPGS2	tubulin polyglutamylase complex subunit 2	L1
1449997_at	TPM3	tropomyosin 3	
1433883_at	TPM4	tropomyosin 4	
1448313_at	TPP1	tripeptidyl peptidase I	L1
1426949_s_at	TPR	translocated promoter region, nuclear basket protein	L1
1456651_a_at	TPR	translocated promoter region, nuclear basket protein	L1
1429117_at	TRADD	TNFRSF1A-associated via death domain	L1
1446940_at	TRAF6	TNF receptor-associated factor 6, E3 ubiquitin protein ligase	L1
1424320_a_at	TRAF7	TNF receptor-associated factor 7, E3 ubiquitin protein ligase	L1
1428178_s_at	TRAPPC6B	trafficking protein particle complex 6B	L1
1424162_at	TRIM29	tripartite motif containing 29	L1
1427476_a_at	TRIM32	tripartite motif containing 32	L1
1433745_at	TRIO	trio Rho guanine nucleotide exchange factor	L1
1457492_at	TRIO	trio Rho guanine nucleotide exchange factor	L1
1423899_at	TRIP12	thyroid hormone receptor interactor 12	L1
1451158_at	TRIP12	thyroid hormone receptor interactor 12	

1449041_a_at	TRIP6	thyroid hormone receptor interactor 6	L1
1424333_at	TRMT10C	tRNA methyltransferase 10 homolog C (S. cerevisiae)	L1
1426016_a_at	TRO	trophinin	L1
1457164_at	TRPA1	transient receptor potential cation channel, subfamily A, member 1	
1460720_at	TRPC4AP	transient receptor potential cation channel, subfamily C, member 4 associated protein	L1
1452615_s_at	TRPT1	tRNA phosphotransferase 1	L1
1428281_at	TRUB1	TruB pseudouridine (psi) synthase homolog 1 (E. coli)	L1
1452105_a_at	TSC2	tuberous sclerosis 2	L1
1416009_at	TSPAN3	tetraspanin 3	L1
1416994_at	TTC1	tetratricopeptide repeat domain 1	L1
1435456_at	TTC28	tetratricopeptide repeat domain 28	L1
1416484_at	TTC3	tetratricopeptide repeat domain 3	L1
1427446_s_at	TTN	titin	
1416311_s_at	TUBA3E	tubulin, alpha 3e	L1
1455868_a_at	TUBGCP2	tubulin, gamma complex associated protein 2	L1
1426707_at	TUBGCP3	tubulin, gamma complex associated protein 3	L1
1440282_at	TULP4	tubby like protein 4	
1421662_a_at	TUSC3	tumor suppressor candidate 3	L1
1420873_at	TWF1	twinfilin, actin-binding protein, homolog 1 (Drosophila)	
1418733_at	TWIST1	twist homolog 1 (Drosophila)	
1448782_at	TXNDC11	thioredoxin domain containing 11	L1
1424486_a_at	TXNRD1	thioredoxin reductase 1	L1
1429971_at	TXNRD2	thioredoxin reductase 2	
1417306_at	TYK2	tyrosine kinase 2	
1434300_at	U2SURP	U2 snRNP-associated SURP domain containing	
1448116_at	UBA1	ubiquitin-like modifier activating enzyme 1	L1

1416280_at	UBA2	ubiquitin-like modifier activating enzyme 2	L1
1424281_at	UBAP2	ubiquitin associated protein 2	L1
1429475_at	UBASH3B	ubiquitin associated and SH3 domain containing B	L1
1419762_at	UBD	ubiquitin D	
1448772_at	UBE2A	ubiquitin-conjugating enzyme E2A	L1
1416477_at	UBE2D2	ubiquitin-conjugating enzyme E2D 2	L1
1453189_at	UBE2I	ubiquitin-conjugating enzyme E2I	
1435384_at	UBE2N	ubiquitin-conjugating enzyme E2N	
1425206_a_at	UBE3A	ubiquitin protein ligase E3A	
1416848_at	UBL5	ubiquitin-like 5	
1454668_at	UBR4	ubiquitin protein ligase E3 component n-recognin 4	L1
1444327_at	UBR5	ubiquitin protein ligase E3 component n-recognin 5	
1460304_a_at	UBTF	upstream binding transcription factor, RNA polymerase I	
1449855_s_at	UCHL3	ubiquitin carboxyl-terminal esterase L3 (ubiquitin thiolesterase)	L1
1448604_at	UCK2	uridine-cytidine kinase 2	L1
1447046_at	UCP2	uncoupling protein 2 (mitochondrial, proton carrier)	L1
1426261_s_at	UGT1A6	UDP glucuronosyltransferase 1 family, polypeptide A6	L1
1448370_at	ULK1	unc-51-like kinase 1 (C. elegans)	L1
1415866_at	UNC45A	unc-45 homolog A (C. elegans)	L1
1429844_at	UPK3BL	uroplakin 3B-like	L1
1454841_at	URB1	URB1 ribosome biogenesis 1 homolog (S. cerevisiae)	L1
1430494_at	URGCP	upregulator of cell proliferation	
1433498_at	URGCP	upregulator of cell proliferation	L1
1450892_a_at	USP4	ubiquitin specific peptidase 4 (proto-oncogene)	L1
1435901_at	USP40	ubiquitin specific peptidase 40	L1
1424056_at	USP48	ubiquitin specific peptidase 48	L1

1429937_at	UTP23	UTP23, small subunit (SSU) processome component, homolog (yeast)	L1
1426892_at	UTRN	utrophin	
1420624_a_at	VAMP8	vesicle-associated membrane protein 8 (endobrevin)	
1427894_at	VASN	vasorin	
1419417_at	VEGFC	vascular endothelial growth factor C	L1
1440739_at	VEGFC	vascular endothelial growth factor C	L1
1417900_a_at	VLDLR	very low density lipoprotein receptor	
1440146_at	VPS13A	vacuolar protein sorting 13 homolog A (S. cerevisiae)	
1416254_a_at	VPS16	vacuolar protein sorting 16 homolog (S. cerevisiae)	L1
1421050_at	VPS25	vacuolar protein sorting 25 homolog (S. cerevisiae)	L1
1427933_at	VPS33B	vacuolar protein sorting 33 homolog B (yeast)	
1415784_at	VPS35	vacuolar protein sorting 35 homolog (S. cerevisiae)	
1429363_at	VPS37A	vacuolar protein sorting 37 homolog A (S. cerevisiae)	L1
1417510_at	VPS4A	vacuolar protein sorting 4 homolog A (S. cerevisiae)	L1
1454825_at	VPS51	vacuolar protein sorting 51 homolog (S. cerevisiae)	L1
1447894_x_at	VPS52	vacuolar protein sorting 52 homolog (S. cerevisiae)	L1
1442699_at	VPS54	vacuolar protein sorting 54 homolog (S. cerevisiae)	
1456810_at	VPS54	vacuolar protein sorting 54 homolog (S. cerevisiae)	
1455050_at	VSTM4	V-set and transmembrane domain containing 4	
1420484_a_at	VTN	vitronectin	L1
1455098_a_at	VTN	vitronectin	
1415799_at	WBP11	WW domain binding protein 11	L1
1430987_s_at	WBP11	WW domain binding protein 11	L1

1427456_at	WDFY3	WD repeat and FYVE domain containing 3	
1423054_at	WDR1	WD repeat domain 1	L1
1451728_at	WDR13	WD repeat domain 13	L1
1453554_a_at	WDR33	WD repeat domain 33	L1
1429174_at	WDR34	WD repeat domain 34	L1
1415770_at	WDR6	WD repeat domain 6	L1
1455940_x_at	WDR6	WD repeat domain 6	L1
1444689_at	WDR67	WD repeat domain 67	
1434793_at	WDR78	WD repeat domain 78	
1426767_at	WDR90	WD repeat domain 90	
1424917_a_at	WIPI1	WD repeat domain, phosphoinositide interacting 1	
1419015_at	WISP2	WNT1 inducible signaling pathway protein 2	
1454805_at	WTAP	Wilms tumor 1 associated protein	L1
1448145_at	WWP2	WW domain containing E3 ubiquitin protein ligase 2	L1
1448278_at	XAB2	XPA binding protein 2	L1
1420011_s_at	XBP1	X-box binding protein 1	L1
1420012_at	XBP1	X-box binding protein 1	
1420886_a_at	XBP1	X-box binding protein 1	L1
1456088_at	XIAP	X-linked inhibitor of apoptosis	L1
1439411_a_at	XPO7	exportin 7	L1
1426993_at	XPR1	xenotropic and polytropic retrovirus receptor 1	L1
1453950_a_at	XRCC2	X-ray repair complementing defective repair in Chinese hamster cells 2	L1
1430134_a_at	YARS2	tyrosyl-tRNA synthetase 2, mitochondrial	L1
1453414_at	YPEL2	yippee-like 2 (Drosophila)	
1427638_at	ZBTB16	zinc finger and BTB domain containing 16	
1416224_at	ZBTB17	zinc finger and BTB domain containing 17	L1
1448705_at	ZBTB22	zinc finger and BTB domain containing 22	L1
1426361_at	ZC3H11A	zinc finger CCCH-type containing 11A	L1

1430568_at	ZC3H13	zinc finger CCCH-type containing 13	L1
1427876_at	ZC3H15	zinc finger CCCH-type containing 15	L1
1428682_at	ZC3H6	zinc finger CCCH-type containing 6	
1437616_x_at	ZDHHC14	zinc finger, DHHC-type containing 14	L1
1421115_a_at	ZDHHC16	zinc finger, DHHC-type containing 16	L1
1452274_at	ZFAND3	zinc finger, AN1-type domain 3	L1
1422031_a_at	ZFAND6	zinc finger, AN1-type domain 6	
1420944_at	ZFP185	zinc finger protein 185	L1
1440890_a_at	ZFP809	zinc finger protein 809	
1449126_at	ZFP90	zinc finger protein 90 homolog (mouse)	
1441348_at	ZFP955A/ZFP955B	zinc finger protein 955B	
1424892_at	ZKSCAN5	zinc finger with KRAB and SCAN domains 5	L1
1445543_at	ZMYM5	zinc finger, MYM-type 5	
1426532_at	ZMYND11	zinc finger, MYND-type containing 11	
1429415_at	ZMYND8	zinc finger, MYND-type containing 8	L1
1453034_at	ZNF251	zinc finger protein 251	
1419165_at	ZNF260	zinc finger protein 260	L1
1452045_at	ZNF281	zinc finger protein 281	L1
1451151_s_at	ZNF410	zinc finger protein 410	
1449510_at	ZNF467	zinc finger protein 467	L1
1434088_at	ZNF496	zinc finger protein 496	L1
1423836_at	ZNF503	zinc finger protein 503	L1
1437483_at	ZNF513	zinc finger protein 513	L1
1438079_at	ZNF627	zinc finger protein 627	L1
1419984_s_at	ZNF644	zinc finger protein 644	L1
1435481_at	ZNF653	zinc finger protein 653	L1
1429217_at	ZNF655	zinc finger protein 655	L1
1451323_at	ZNF7	zinc finger protein 7	
1438026_at	ZNF778	zinc finger protein 778	L1
1416370_at	ZSCAN21	zinc finger and SCAN domain containing 21	L1
1417240_at	ZYX	zyxin	L1
1421653_a_at			

1421754_at			
1422426_at			L1
1425421_at			L1
1425424_at			L1
1426113_x_at			L1
1427437_at			
1427455_x_at			
1427653_at			
1427809_at			L1
1428301_at			
1429381_x_at			
1430308_at			L1
1430404_at			
1430945_at			L1
1435150_at			L1
1437073_x_at			
1438049_at			L1
1438068_at			
1438257_at			
1438370_x_at			L1
1438783_at			
1440078_at			L1
1440360_at			L1
1440494_at			L1
1440742_at			L1
1441147_at			
1441247_at			
1441386_at			
1441505_at			
1441627_at			
1441718_at			L1
1442217_at			
1442463_at			
1442505_at			L1
1442535_at			
1442992_at			
1443007_at			L1
1443010_at			
1443241_at			L1
1443249_at			L1
1443293_at			L1

1443302_at			
1443325_at			L1
1444192_at			
1444490_at			
1444750_at			
1444856_at			
1444920_at			L1
1445144_at			
1445461_at			L1
1445518_at			L1
1445579_at			
1445666_at			L1
1445807_at			
1445935_at			L1
1445958_at			L1
1446040_at			L1
1446411_at			L1
1446530_at			
1446593_at			L1
1446598_at			
1446850_at			L1
1446914_at			
1447064_at			L1
1447147_at			
1447156_at			
1447179_at			L1
1447316_at			L1
1448018_at			L1
1452405_x_at			L1
1452463_x_at			
1452731_x_at			
1454002_at			L1
1455569_at			L1
1455930_at			
1456453_at			
1456677_at			L1
1456787_at			L1
1456880_at			L1
1457141_at			
1457173_at			L1
1457348_at			L1

1457483_at			
1457750_at			
1457859_at			
1457934_at			L1
1458065_at			
1458257_at			L1
1458288_at			L1
1458457_at			L1
1458589_at			L1
1458703_at			
1458850_at			L1
1458980_at			L1
1459358_at			L1
1459733_at			
1459968_at			L1
1460117_at			
1460133_at			L1
